# Revisiting Proteasome Inhibitors: Molecular Underpinnings of Their Development, Mechanisms of Resistance and Strategies to Overcome Anti-Cancer Drug Resistance

**DOI:** 10.3390/molecules27072201

**Published:** 2022-03-28

**Authors:** Carlota Leonardo-Sousa, Andreia Neves Carvalho, Romina A. Guedes, Pedro M. P. Fernandes, Natália Aniceto, Jorge A. R. Salvador, Maria João Gama, Rita C. Guedes

**Affiliations:** 1 Research Institute for Medicines (iMed.ULisboa), Faculty of Pharmacy, Universidade de Lisboa, 1649-003 Lisboa, Portugal; carlota.leonardo.sousa@gmail.com (C.L.-S.); amcarvalho@ff.ulisboa.pt (A.N.C.); rpaguedes@gmail.com (R.A.G.); pedrompf92@gmail.com (P.M.P.F.); nataliaaniceto@ff.ul.pt (N.A.); 2 Center for Neuroscience and Cell Biology (CNC), Center for Innovative Biomedicine and Biotechnology (CIBB), Laboratory of Pharmaceutical Chemistry, Faculty of Pharmacy, University of Coimbra, 3004-504 Coimbra, Portugal

**Keywords:** ubiquitin–proteasome pathway, proteasome inhibitors, mechanisms of resistance, innate resistance, acquired resistance, multiple myeloma, cancer

## Abstract

Proteasome inhibitors have shown relevant clinical activity in several hematological malignancies, namely in multiple myeloma and mantle cell lymphoma, improving patient outcomes such as survival and quality of life, when compared with other therapies. However, initial response to the therapy is a challenge as most patients show an innate resistance to proteasome inhibitors, and those that respond to the therapy usually develop late relapses suggesting the development of acquired resistance. The mechanisms of resistance to proteasome inhibition are still controversial and scarce in the literature. In this review, we discuss the development of proteasome inhibitors and the mechanisms of innate and acquired resistance to their activity—a major challenge in preclinical and clinical therapeutics. An improved understanding of these mechanisms is crucial to guiding the design of new and more effective drugs to tackle these devastating diseases. In addition, we provide a comprehensive overview of proteasome inhibitors used in combination with other chemotherapeutic agents, as this is a key strategy to combat resistance.

## 1. Introduction

The quality and quantity of proteins within a cell must be tightly regulated according to cellular needs or physiological demand. The ubiquitin–proteasome pathway (UPP) is critical for the maintenance of intracellular protein homeostasis in physiological conditions, as well as during adaptive stress responses, and is responsible for the regulation of a wide variety of signaling pathways [1]. Accordingly, impairment of the UPP has been associated with several pathological conditions that include neoplastic disorders [2]. Cancer cells are characterized by the loss of cell cycle checkpoint control and are often subjected to elevated levels of stress because of hyperactivation of oncogenic signaling and/or adverse microenvironmental conditions. Therefore, transformed cells rely to a great extent on the correct function of UPP for survival and proliferation [2].

Just after the discovery of the UPP and its relevance to protein and cellular homeostasis, preclinical studies on the putative role of proteasome inhibitors as critical agents for modulating cancer cell death have begun [3]. The proteasome was identified and validated as a pivotal target in protein quality control and turnover, cell-cycle regulation, cell differentiation, and apoptosis. Since then, three proteasome inhibitors have been approved by the US Food and Drug Administration Agency (FDA) and the European Medicine Agency (EMA), Velcade^®^ (bortezomib), Kyprolis^®^ (carfilzomib), and Ninlaro^®^ (ixazomib), as new drugs to treat multiple myeloma (MM) and mantle-cell lymphoma (MCL) [4,5,6,7,8,9].

**Figure 1:**
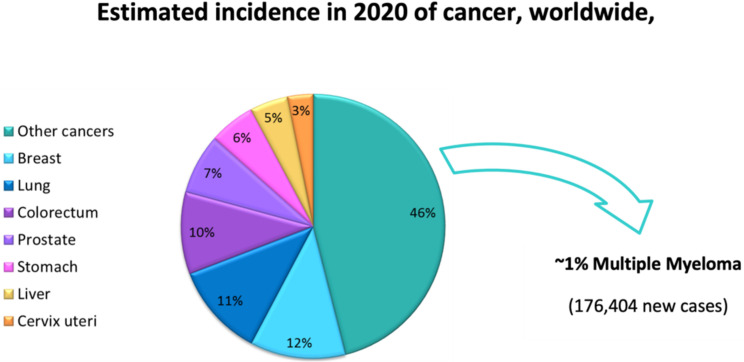
Estimated new cases of cancer in 2020, worldwide, both sexes and all ages. MM represents 1% of all types of cancer. Adapted from: Ferlay, J.; Ervik, M.; Lam, F.; Colombet, M.; Mery, L.; Piñeros, M.; Znaor, A.; Soerjomataram, I.; Bray, F. Global Cancer Observatory: Cancer Today; Lyon, France, 2020.

**Figure 2:**
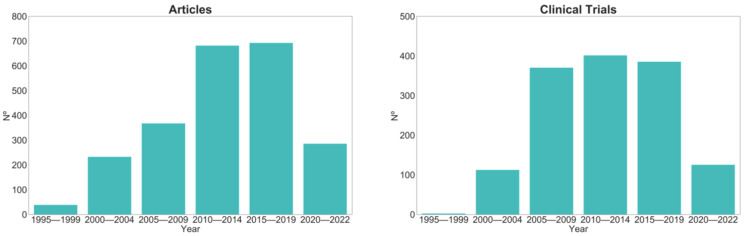
Articles published from 1995 reported on PubMed, entering the search terms “proteasome inhibitors” and “resistance” in all fields, and clinical trials whose intervention/treatment includes PIs (bortezomib, carfilzomib, ixazomib, marizomib, delanzomib or oprozomib) starting from 1995. Adapted from: ClinicalTrials.gov (https://clinicaltrials.gov/ct2/home accessed on 22 February 2022) and PubMed (https://pubmed.ncbi.nlm.nih.gov/ accessed on 22 February 2022).

MM is the second most frequent hematological malignancy with an age-adjusted incidence of approximately 7.1 per 100,000 persons per year in the USA (~1.8% of all cancers), based on 2014–2018 cases [10] and approximately 2.9 per 100,000 persons in Europe, based on 2020 cases [11] (Figure 1). This malignancy is described as an expansion of dysfunctional terminal differentiated plasma cells in the bone marrow. MM cells show strong bone marrow dependence, extensive somatic hypermutation of immunoglobulin genes and absence of IgM expression [12]. Therefore, MM is characterized by aberrant proliferation of bone marrow plasma cells that commonly produce a high amount of monoclonal immunoglobulin, leading to functional impairments in different organs, namely anemia, bone disease, renal dysfunction and hypercalcemia [13]. MM is a very heterogeneous disease with median survival ranging from 2 to 10 years and is characterized by remission periods alternating with relapse/progression phases, finally leading to refractory disease [13]. The improved understanding of the mechanisms involved in MM has led to more effective therapeutic strategies such as proteasome inhibitors (PIs), namely bortezomib, carfilzomib, and ixazomib, and immunomodulatory drugs (IMIDs), including thalidomide, lenalidomide, and pomalidomide, that allowed extension of the median overall patient survival to over 8 years [13,14] (Figure 2). These two classes of drugs revolutionized the treatment of MM due to their strong synergistic action. Despite this improvement in first-line therapy, almost all patients eventually relapse, the outcome progressively worsens, and the disease is still generally considered incurable.

MCL is a rare but aggressive disease, with a poor prognosis and limited survival, resulting from a malignant transformation of a B lymphocyte in the outer edge of a lymph node follicle (the mantle zone). MCL represents 3% to 10% of all newly diagnosed non-Hodgkin lymphoma (NHL) cases with an incidence of approximately 1 per 100,000 persons in the USA. This NHL is molecularly characterized by the chromosomal translocation t(11;14)(q13;q32) that results in a constitutional overexpression of the cell cycle regulator protein cyclin D1 with consequent cell cycle dysregulation [15]. This translocation is the initial event of the lymphomagenesis, but tumor cells can accumulate additional alterations that will ultimately produce the aggressive phenotype in disease progression. An increasing number of biologically targeted therapies are improving MCL treatment options in both first-line and relapsed conditions, namely the proteasome inhibitor bortezomib (Velcade^®^), the mechanistic target of rapamycin (mTOR) inhibitor temsirolimus (Torisel^®^), lenalidomide and ibrutinib, the four drugs currently licensed for MCL [15]. However, despite the recent advances in therapy, relapses are still frequent and associated with a poor prognosis. Generally, the disease is characterized by rapid relapses and poor long-term outcomes due to the development of resistance [16].

Both MM and MCL are aggressive diseases largely regarded as incurable, mostly due to the development of resistance. The results obtained in clinical trials with PIs grant them the status of promising therapeutics to be further investigated in a permanent search for new chemical entities able to offset the upregulation of the proteasome encountered in these diseases. MM patients showed considerably improved outcomes with the use of both first- and second-generation PIs that elicited deep initial responses in these patients. Additionally, primary resistance is also a drawback to the use of PIs (as demonstrated also for solid tumors) where, regardless of the promising pre-clinical data obtained, clinical data have been shown to be disappointing. Thus, this reinforces the importance of understanding drug resistance mechanisms associated with PIs, to acquire novel insights critical to further maximize the effectiveness of this class of drugs and improve therapies. Herein, we present a brief overview of the classes of PIs developed so far and critically discuss the advances and challenges related to the use of PIs in the clinic.

## 2. Ubiquitin–Proteasome Pathway (UPP)

The dynamic state of intracellular proteins is maintained by a perfect equilibrium between protein synthesis and protein degradation. The UPP is the primary proteolytic pathway responsible for the degradation of short-lived proteins, providing the specificity and temporal control needed for fine-tuning the steady-state levels of many regulatory proteins [17,18]. Therefore, in addition to mediating the degradation of damaged and misfolded intracellular proteins, through the regulation of protein turnover, the UPP also regulates the function of several proteins, including transcription factors, many of which are critical in the determination of cell fate [19,20]. The UPP plays a crucial role in numerous cellular functions including regulation of cell cycle and division, DNA damage repair, membrane trafficking, cellular stress response, intracellular signaling and apoptosis [17,18,20,21].

**Figure 3:**
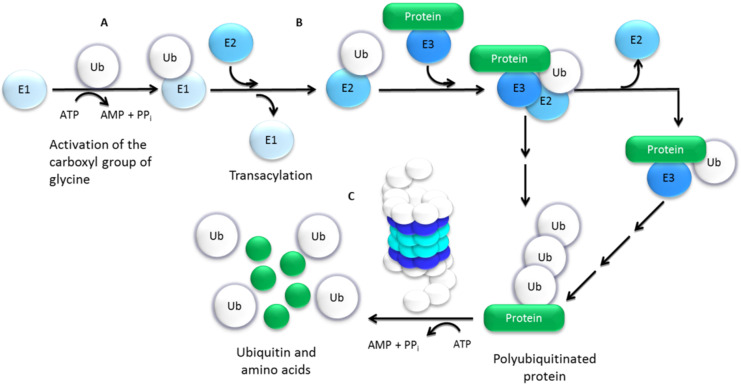
(**A**) Ubiquitin–proteasome pathway of protein degradation. In the ubiquitination process, there is activation of the carboxyl group of glycine found at the C-terminal residues of ubiquitin (Ub), catalyzed by E1 (formation of a thiol-ester bond between E1 and ubiquitin), with the hydrolysis of ATP to AMP and with the release of one PPi molecule. (**B**) After activated ubiquitin is transferred, by transacylation, to the thiol group of the enzyme E2. Then E3 recognizes the protein to be degraded and facilitates E2 to transfer the ubiquitin to the protein, with the formation of an isopeptide covalent bond between the C-terminal glycine residues of ubiquitin and a lysine residue of the protein. (**C**) From multiple cycles of ubiquitination, a polyubiquitinated protein is obtained. In the degradation process, the polyubiquitinated proteins are unfolded and recognized by the 26S proteasome, with ATP hydrolysis to AMP.

The degradation of proteins by the UPP is a sequential process involving an initial step of ubiquitin (Ub) conjugation to the protein substrate followed by the degradation of the polyubiquitinated protein through the 26S proteasome complex, with the release of free Ub, mediated by deubiquitinating enzymes (DUBs) (Figure 3) [17,18,20,21]. Ub conjugation, or ubiquitination, is a post-translational modification that consists of the covalent attachment of one (monoubiquitination) or several (polyubiquitination) Ub molecules to a protein and depends on the concerted, successive action of three types of enzymes: the ubiquitin-activating enzyme (E1), the ubiquitin-conjugating enzymes (E2) and the ubiquitin-protein ligases (E3) [17,21]. Polyubiquitination generally serves as a recognition signal for proteolytic degradation by the 26S proteasome. The 26S proteasome is a large (~2.5 MDa) multimeric protease complex, generally conserved in eukaryotes, both structurally and functionally. It is formed by a key 20S core particle (CP), which contains the protease subunits, capped on one or both ends by the 19S regulatory particles (RP), that regulate the proteolytic function of the protease core [17,22]. PIs typically target the 20S CP of the proteasome, so this will be expanded in further detail next.

### The 20S Proteasome Core Particle

**Figure 4:**
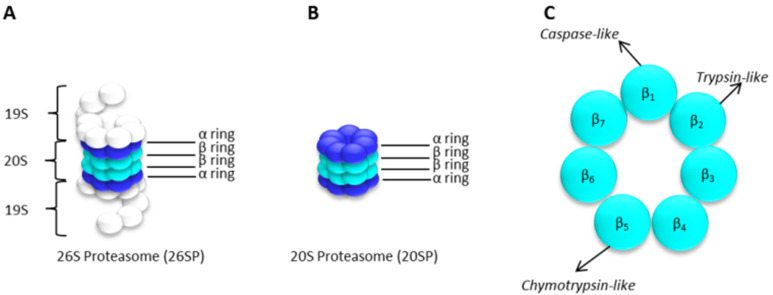
(**A**) 26S proteasome. The 26S proteasome consists of a multimeric protease with 2 19S RP and the 20S CP. (**B**) The 20S CP is constituted by 2 α heptameric rings and 2 β heptameric rings. (**C**) The catalytic subunits are located in 3 distinct β subunits, in both β heptameric rings.

**Figure 5:**
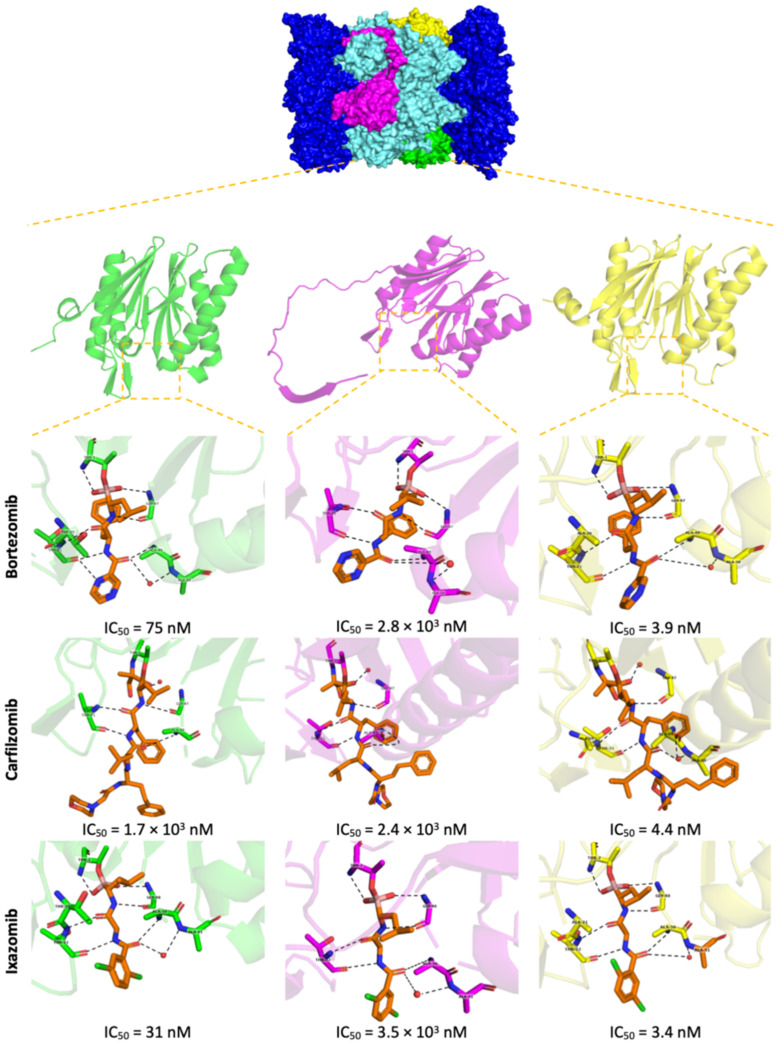
Three β catalytic subunits of 20S CP complexed or not with the three approved inhibitors. The β1, β2, and β5 subunits are colored in green, magenta, and yellow, respectively. The inhibitors are colored in orange; hydrogen bonds are shown as black dashed lines and water molecules are represented as red spheres. These images were generated with the crystallographic structures whose PDB IDs are 4R67, 5LE5, 5LF3, and 5LF7. Source: Report RPT-01200 Amendment 2 and Study TR-0004-171.

The 20S CP is a barrel-shaped structure that corresponds to the catalytic component of the proteolytic machinery of the 26S proteasome. It is formed by four stacked heptameric rings, each ring consisting of seven α- or β-type subunits (Figure 4). The two inner β-rings contain the proteolytic active sites (β_1–7_), facing inward into the proteolytic chamber (the inside of the “barrel”). Three of which—β_1_, β_2_ and β_5_—are endowed with caspase-, trypsin- and chymotrypsin-like activities, respectively [22]. The β5 subunit with chymotrypsin-like activity is responsible for the cleavage of peptide bonds after a hydrophobic residue; the β2 subunit displays trypsin-like activity and cleaves the peptide bonds after a basic residue; and the β1 subunit has caspase-like or post-acidic-like activity cleaving the peptide bonds after an acidic residue [3,22]. The three catalytic subunits contain an N-terminal residue, Thr1, whose hydroxyl group acts as a nucleophile and interacts with the peptides of the proteins to be degraded (Figure 4 and Figure 5) [22,23]. The α-subunits (α_1–7_) in the outer rings of the 20S CP can recognize and direct polyubiquitinated substrates into the proteolytic chamber. One or two 19S RP can be attached to the surface of the outer α-rings of the 20S CP to form the 26S proteasome holoenzyme (Figure 4).

The 19S RP is a 700 kDa ring-shaped complex also called proteasome activator 700 (PA700) and is formed by two substructures, a lid and a base, with multiple subunits, as shown in Figure 4A. The 19S RP recognizes polyubiquitinated proteins and promotes either their ATP-dependent unfolding or the dismantling of ubiquitin chains, catalyzed by proteasome-associated DUBs [2]. The 20S CP generally mediates the cleavage/degradation of polyubiquitinated protein substrates that have been unfolded by the 19S RP into small peptides and amino acids [2,22]. However, in some cases, the 20S CP can act alone through ubiquitin-independent degradation pathways, still being functional towards certain proteins [3,22,24,25].

## 3. Proteasome Inhibitors (PIs) in Cancer Therapy

### 3.1. Aldehydes

**Figure 6:**
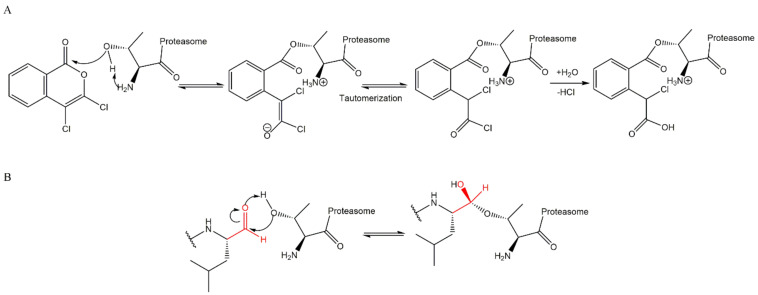
Mechanism of 20S CP inhibition by 3,4-dichloroisocoumarin (**A**) and by a peptide aldehyde (**B**). The hydroxyl group from Thr1 reacts with the carbonyl group of the inhibitor with the formation of a hemiketal, which is similar to a transition state of enzymatic reaction [27,29,30].

3,4-dichloroisocoumarin is a potent irreversible inhibitor of serine proteases and was one of the first compounds demonstrated to inhibit the 20S CP activity by covalently binding to the N-terminal Thr1 of the catalytic subunits. The exact mechanism of interaction between this inhibitor and the proteasome is not fully known, but because it contains a cyclic ester, such as β-lactone, it is suggested that the proteasome inhibition occurs through the formation of a non-hydrolysable acyl (Figure 6A). However, this compound demonstrated high toxicity and low selectivity for 20S CP in in vivo studies and human clinical trials [26,27,28]. Since then, analogs of 3,4-dichloroisocoumarin were synthesized, however, with poor inhibition directed at 20S CP [26].

Later, calpain inhibitors I and II, the first synthetic inhibitors of serine and cysteine proteases, also demonstrated efficiency in reversible inhibition of proteasome’s proteolytic activity. However, these compounds also present the disadvantage of not being selective for the 20S CP. For instance, the calpain I inhibitor ALLN is 25-fold more potent against cathepsin B and calpain than against proteasome [27,28].

This drawback prompted the development of peptide aldehyde inhibitors, known for having a fast cellular uptake and slow binding to the β5 proteasome subunit (Figure 6B). However, these compounds dissociate rapidly from 20S CP and are easily inactivated by oxidation [27,28].

**Figure 7:**
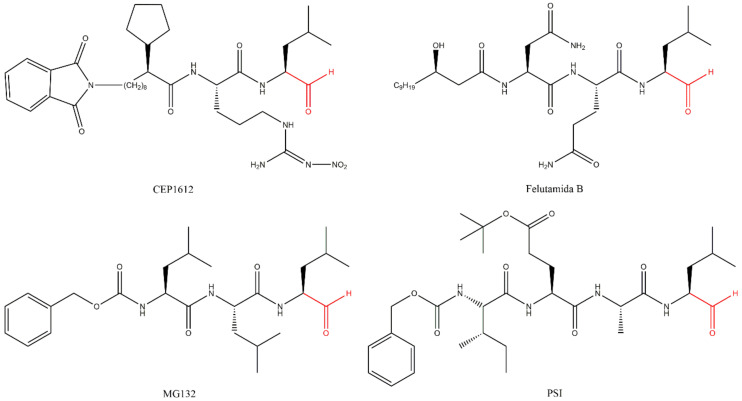
Examples of aldehydes inhibitors of 20S CP [27,31].

The peptide aldehyde inhibitors CEP1612, MG115, MG132 and PSI (Figure 7) are inhibitors of serine and cysteine proteases; however, they also inhibit the 20S CP and have increased selectivity to it when compared to the previously described inhibitors [27]. This has led to their frequent use in pre-clinical studies to evaluate the effects of PIs in several experimental models.

Since aldehyde inhibitors demonstrated a moderate reactivity and were not sufficiently selective to the 20S CP (also inhibiting serine and cysteine proteases), other inhibitor classes were explored [32].

### 3.2. Boronates

Widely used in the synthesis of serine protease inhibitors, the boronic esters and acids were also demonstrated to reversibly inhibit the 20S CP. Consequently, potent and selective di- and tripeptidyl boronic acid inhibitors were developed and shown to be more potent than aldehydes. The boronate inhibitors are also not easily inactivated by oxidation and are more selective to the 20S CP in comparison to common proteases [27,33].

**Figure 8:**
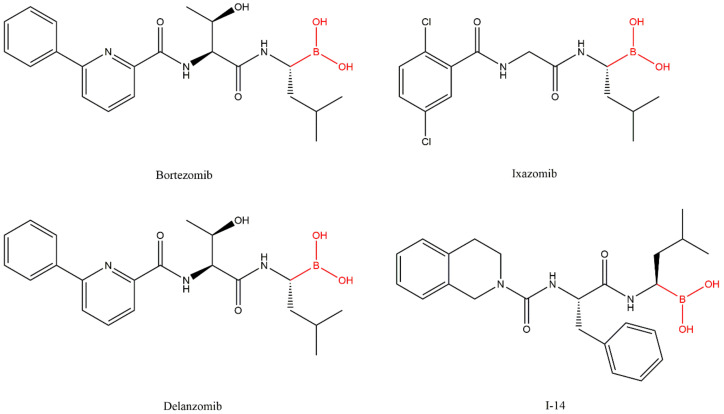
Examples of boronate inhibitors of 20S CP [31,37,38].

**Figure 9:**
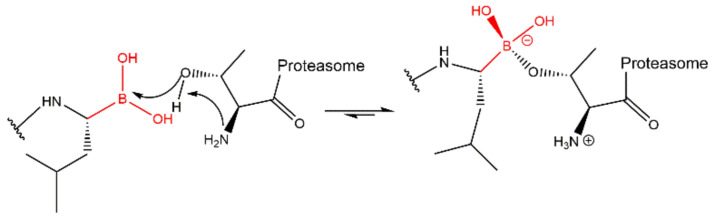
Mechanism of 20S CP inhibition by boronate inhibitors. The hydroxyl group from Thr1 reacts with the boronate from the inhibitor, with the formation of a borate, a tetrahedral boron anion [27,29].

Bortezomib (PS341/MG341) (Figure 8), an analog of the dipeptide boronic acid, was synthesized in 1995 by Myogenics and later acquired by Millennium Pharmaceuticals, Inc. (now acquired by Takeda Pharmaceutical Company Limited) [34,35]. Bortezomib inhibits the β5 subunit of 20S CP reversibly through the presumable formation of a complex between the boronic acid and the Thr1 hydroxyl group which results in the formation of a tetrahedral adduct similar to peptide aldehydes (Figure 9) [27,28]. To a lesser extent, bortezomib also targets the β1 subunit, while the β2 site is left relatively untouched [28]. Bortezomib was the first 20S CP inhibitor approved in 2003 by the FDA for the treatment of MM and, in 2006, for the treatment of MCL in patients who have received at least one prior therapy [6,36]. It received the first authorization by EMA in 2004 and is currently authorized as a monotherapy or in combinatory therapies (with melphalan, prednisone, dexamethasone, thalidomide and pegylated liposomal doxorubicin) for the treatment of MM; combinations with rituximab, cyclophosphamide, doxorubicin, and prednisone can be used for the treatment of MCL in untreated patients who cannot have blood stem-cell transplantation [9].

However, bortezomib shows toxicity related to proteasomal inhibition in non-target tissues (e.g., 28% of the patients have grade 3 thrombocytopenia and induce peripheral neuropathy, of grade 3 in 12%, and any grade in 31% of the patients [39]), limited activity in solid tumors, innate and acquired resistance (being necessary to combine other chemotherapeutic agents to increase the cytotoxicity) and the necessity for subcutaneous or intravenous administration, since it is not orally bioavailable.

In order to overcome these limitations, second-generation inhibitors with improved ADME properties were developed, namely ixazomib and delanzomib [34].

Ixazomib (MLN-9708/2238) (Figure 8) is a reversible inhibitor that binds to proteasome’s β5 subunit. It was approved by the FDA in 2015, received the first authorization in 2016 by the EMA and, in 2017, it was given “conditional approval” (more information about its benefits is still required) for the treatment of MM in combination with lenalidomide and dexamethasone in patients who have received at least one prior therapy [4,7]. It is the first 20S CP inhibitor approved for oral delivery. However, adverse effects such as peripheral neuropathy were also reported. It is administered in the form of a prodrug (ixazomib citrate), and it is rapidly hydrolyzed in the plasma. Compared with bortezomib, this inhibitor displays similar selectivity and potency for the β5 subunit; however, it has a substantially shorter half-life which may improve biodistribution [34,36].

Delanzomib (CEP-18770) (Figure 8) is a 20S CP inhibitor selective to the β5 subunit, with reversible inhibition comparable to bortezomib, which can be administered orally or intravenously. Phase I clinical trials for the treatment of MM, solid tumors and lymphomas, in patients with advanced solid tumors and MM, and results published in 2013 show that this inhibitor has a favorable safety profile with less neurotoxicity compared with bortezomib [40,41]. In 2016, a phase I/II study was conducted to determine the maximum tolerated dose of delanzomib and the efficacy and safety in patients with relapsed and refractory MM. The authors observed that the disappointing efficacy does not warrant the introduction of delanzomib for the treatment of MM [42,43]. In 2016, a phase I/II study with the objective of determining the maximum tolerated dose of delanzomib in combination with lenalidomide and dexamethasone in patients with relapsed or refractory MM was carried out. However, the study was terminated [44].

Because some inhibitors such as syringoline, α’,β’-epoxyketone and vinyl sulfone (discussed below) exhibit urea in their structure, a class of peptide boronic acid inhibitors containing urea were synthesized. It was found that the inhibitor I-14 (Figure 8) showed excellent in vitro and in vivo antitumor activities, with relatively low toxicity and with appropriate pharmacologic properties. Compared with bortezomib, this compound demonstrated higher potency in inhibition of the β5 subunit of the 20S CP and better pharmacokinetic profile in in vivo assays with mice (it is metabolically more stable than bortezomib).

### 3.3. α’,β’-Epoxyketones

**Figure 10:**
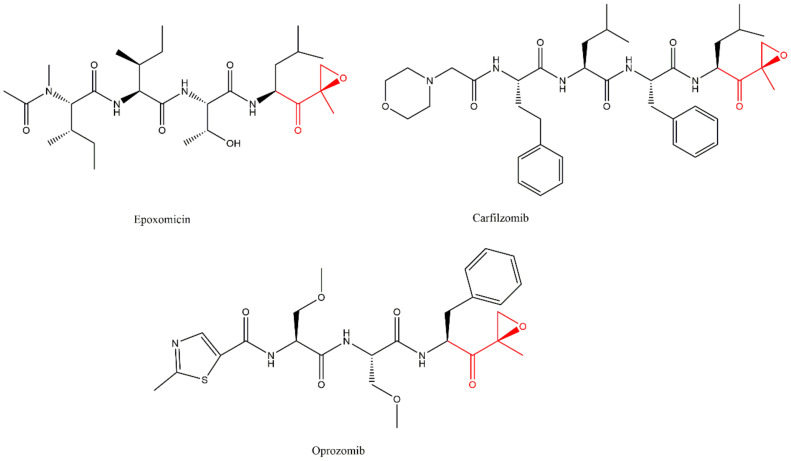
Examples of α’,β’-epoxyketone inhibitors of 20S CP [27,31,53].

In the search for antitumor agents with specific activity against B16 murine melanoma, the natural α’,β’-epoxyketone eponomycin from *Streptomyces hygroscopicus* and epoxomicin (Figure 10) from the actinomycete strain Q996-17 were identified. These compounds demonstrated antitumor activity by inhibiting 20S CP [28].

Carfilzomib (PR-171) (Figure 10) is an α’,β’-epoxyketone inhibitor which was approved by the FDA in 2012 and received the first authorization by the EMA in 2015, currently being authorized for the treatment of MM together with the lenalidomide plus dexamethasone or with dexamethasone alone or daratumumab plus dexamethasone in patients who have received at least one previous treatment [5,8]. According to the FDA, it can also be used in monotherapy for the treatment of MM in patients who have received one or more lines of therapy.

When compared to bortezomib, carfilzomib exhibits equal potency and greater selectivity to the β5 subunit. Although carfilzomib also requires intravenous administration, it presents lower neurotoxicity, most likely due to the higher selectivity to the β5 subunit [34,36].

Oprozomib (ONX-0912, PR-047) (Figure 10), another α’,β’-epoxyketone inhibitor, is orally bioavailable and exhibits similar potency to carfilzomib in cytotoxicity assays. It resembles the in vitro anti-tumor activity to carfilzomib (cancer cell lines and primary cells), and it enhances the anti-myeloma activity of bortezomib [34,36]. Two clinical trials have been completed for this compound: a phase I study to evaluate the safety and tolerability of oprozomib in patients with advanced refractory or recurrent solid tumors; a phase Ib/II study to evaluate the combination therapy of oprozomib with melphalan and prednisone in transplant-ineligible patients with newly diagnosed MM [45,46]. Five clinical trials were terminated due to the identification that the safety profile and pharmacokinetic characteristics of the formulation used in all oprozomib studies required further optimization: two studies of phase Ib/II to determine the maximum tolerated dose, activity and safety of oprozomib in patients with hematologic malignancies and only in relapsed and/or refractory MM; a phase I study to evaluate the effect of food on the pharmacokinetics of oprozomib, the drug–drug interaction of oprozomib with midazolam, and the safety and tolerability of oprozomib in patients with advanced malignancies; and two phase Ib/II and Ib/III studies to evaluate combinatorial therapies of oprozomib with other chemotherapeutic drugs (dexamethasone; lenalidomide; cyclophosphamide; pomalidomide) in patients with MM [47,48,49,50,51]. Currently, a phase I clinical trial is ongoing (but not yet recruiting participants) to evaluate the safety, tolerability, pharmacokinetics, and efficacy of two formulations of oprozomib (immediate release and gastro-retentive formulations) plus pomalidomide and dexamethasone in patients with relapsed/refractory MM [52].

**Figure 11:**
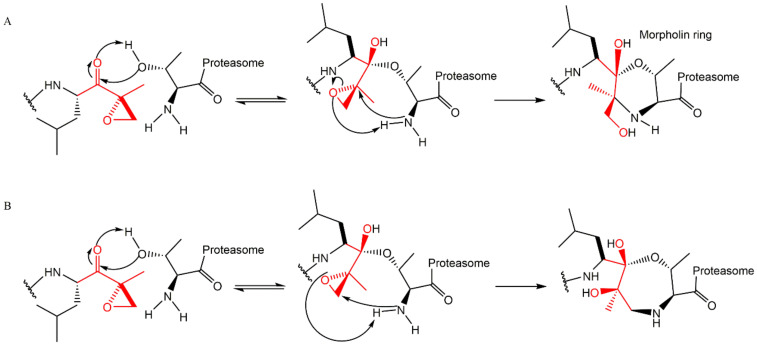
Mechanism of proteasome inhibition by α’,β’-epoxyketone inhibitors. The hydroxyl and amine groups from Thr1 react, respectively, with the carbonyl group and α-carbon or β-carbon of epoxide group from inhibitor, promoting the formation of a 6-membered ring (**A**) and 7-membered ring (**B**), respectively [27,29,57].

This inhibitor class binds covalently and irreversibly to 20S CP through the interaction of the hydroxyl and the amide groups from Thr1 (Figure 11) where the N-terminal amine attacks the epoxide α-carbon, yielding a 6-membered ring (Figure 11A) [27,29,54,55,56]. However, high-resolution crystallography of human 20S CP in complex with oprozomib, dihydroeponemycin (epoxomicin analog), and epoxomicin performed by Schrader et al. [57] suggests that the inhibition reaction yields a 7-membered ring product through a nucleophilic attack by the N-terminal amine of the epoxide β carbon (Figure 11B). This mechanism is different from other classes and characterizes the α’,β’-epoxyketone inhibitors as more selective against the proteasome (because other proteases, which are common targets for many 20S CP inhibitors, do not contain a nucleophilic amino terminal residue) [27,54].

### 3.4. Non-Covalent Macrocyclics

**Figure 12:**
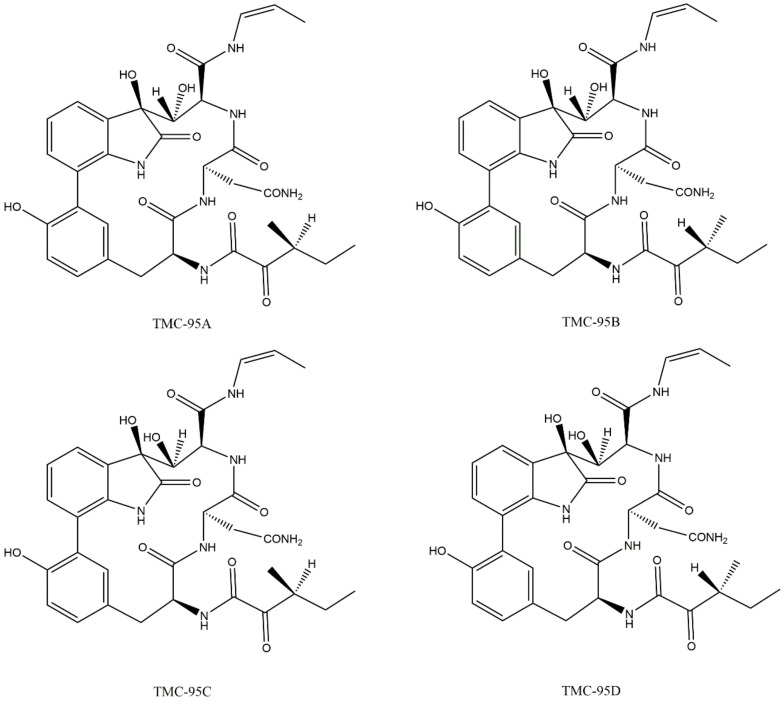
Examples of macrocyclic inhibitors of 20S CP [59].

In 2000, several 20S CP inhibitors from *Apiospora montagnei Sacc*. TC 1093 (TMC-95 A to D) were isolated. In spite of the fact that these inhibitors exhibit uncommon macrocyclic characteristics, they demonstrated a capacity to inhibit the β5 subunit of 20S CP and did not inhibit other proteases (such as calpain II, cathepsin L, and trypsin) [58,59]. Groll et al. [60] described crystal structures with the TMC-95A inhibitor bound to yeast 20S CP, where the inhibitor is bound non-covalently to all proteolytic active β-subunits, without modifying their N-terminal threonines, binding through a tight network of hydrogen bonds which connects the ligand with the β subunits [27,28,60]. Figure 12 illustrates the chemical structure of the inhibitors TMC-95 A to D.

### 3.5. α-Ketoaldehydes and α-Ketoamides

**Figure 13:**
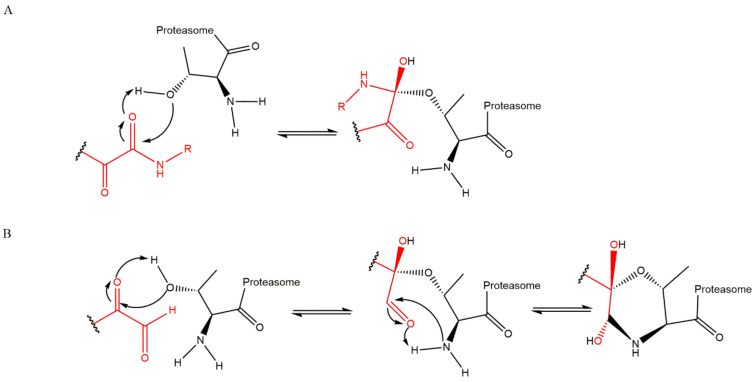
(**A**) Mechanism of proteasome inhibition by α-ketoamide inhibitor, where the hydroxyl from Thr1 reacts with ketoamide group from inhibitor, with the formation of a hemiketal. (**B**) Mechanism of proteasome inhibition by α-ketoaldehyde inhibitor, where the hydroxyl and amine groups from Thr1 react, respectively, with ketone and aldehyde groups from inhibitor, promoting the formation of a 6-membered ring [29,30,55].

**Figure 14:**
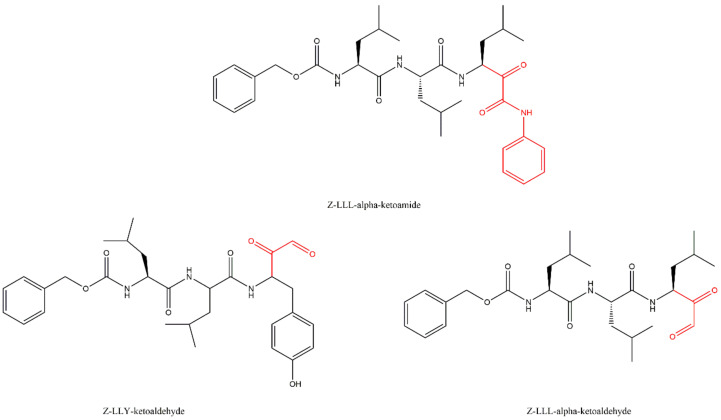
Examples of α-ketoaldehyde (Z-LLL-α-ketoamide) and α-ketoamide (Z-LLY-ketoaldehyde and Z-LLL-α-ketoaldehyde) inhibitors. Adapted from: [56,57].

The α-ketoaldehyde and α-ketoamide peptides are covalent reversible inhibitors of the 20S CP which have been widely ignored for a long time because their benefits were not as evident as other classes of inhibitors. However, a study by Stein et al. [56] concluded that α-ketoamides are the most potent reversible PIs. These compounds may be able to penetrate deeper into solid tissue, thereby making them promising candidates for a range of tumor subtypes broader than those targeted by bortezomib and carfilzomib. These compounds may also have applications as autoimmune disorder therapies. The mechanism of proteasome inhibition by α-ketoamides involves the formation of a reversible hemiketal (Figure 13A). The mechanism of proteasome inhibition by α-ketoaldehydes resembles the mechanism of inhibition by α’,β’-epoxyketones due to the interaction of the inhibitor with the hydroxyl and the amide groups from Thr1 of the 20S CP, leading to the formation of a 6-membered ring (Figure 13B). Like α’,β’-epoxyketones, peptide α-ketoaldehydes also have major selectivity to the 20S CP. They show *K*_i_ values more than 1000-fold higher in the inhibition of serine proteases, which is the case for chymotrypsin and subtilisin (for example), compared to aldehyde peptides because those serine proteases lack the amino terminal nucleophilic residue as part of their active sites. However, α-ketoaldehydes are reversible inhibitors and are thus less potent than α’,β’-epoxyketone, β-lactone and boronate inhibitor classes [27,29]. Figure 14 illustrates examples of α-ketoaldehyde and α-ketoamide inhibitors.

### 3.6. Peptide Vinyl Derivatives

**Figure 15:**
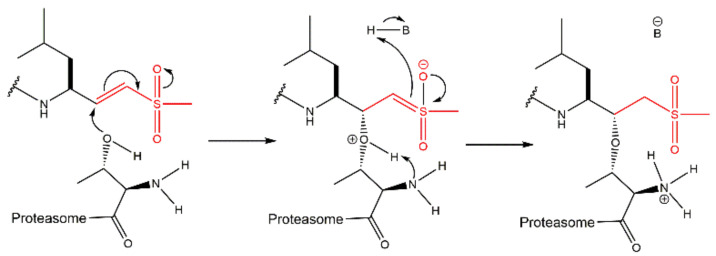
Mechanism of proteasome inhibition by a peptide vinyl derivate inhibitor, in the example, with sulfone as an electron withdrawing group and which function as Michael acceptor of the hydroxyl group from Thr1 [27,29].

The vinyl peptide derivatives have electron withdrawing groups (sulfone or ester) in the C-terminal which behave as Michael acceptors of the catalytic Thr1 hydroxyl group, promoting the formation of a covalent irreversible bond (Figure 15) [61].

**Figure 16:**
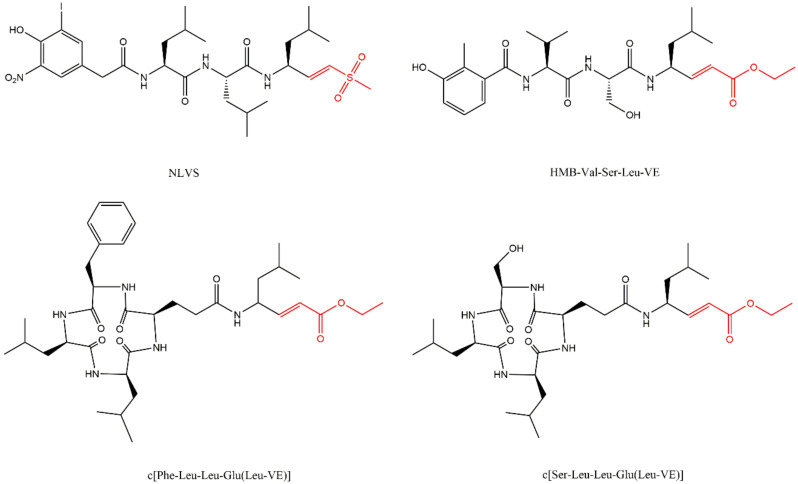
Example of vinyl sulfone inhibitor (NLVS) and vinyl esters inhibitors, 2 cyclics (c[Phe-Leu-Leu-Glu(Leu-VE)] and c[Ser-Leu-Leu-Glu(Leu-VE)], which present more selectivity for β5 and β1 subunits, respectively) [33,64,68,69].

The peptide vinyl sulfones were first described by Nazif and Bogyo [62] and are characterized by their lower reactivity when compared to aldehydes. The compounds AdaAhx_3_-Leu-Leu-Leu-VS, NIP-Leu-Leu-Asn-VS, and NLVS (NIP-Leu-Leu-Leu-vinyl-sulfone) are three examples of peptide vinyl sulfones (Figure 16).

Since they are easier to synthesize than other irreversible PIs, and can be coupled with radioisotopes and fluorescent probes, there is interest in their use as probes to evaluate proteasome activity for in vitro studies in different cells and tissues. For this purpose, various vinyl sulfone inhibitors have been synthesized containing tyrosine or a nitrophenyl group in order to facilitate the radioiodation process, obtaining, e.g., [^125^I]NIP-Leu-Leu-Asn-VS, [^125^I]Tyr-Leu-Leu-Leu-VS e Ada-[^125^I]Tyr-Ahx3-Leu-Leu-Leu-VS, which react with all three catalytic β subunits [27,28,63].

Based on the synthesis of an arecoline derivative (1,2,5,6-Tetrahydropyridine-3-carbonyl-Val-Ser-Leu-benzylamide) which inhibits the β2 and β5 subunits, the Tomatis group [64] identified tripeptide vinyl esters as a class of selective inhibitors of proteasome trypsin-like activity. In this class, HMB−Val-Ser-Leu-VE (Figure 16), HMB-Leu-Leu-Leu-VE and Z-Val-Ser-Leu-VE were the most potent inhibitors, from which other vinyl ester pseudotripeptide analogs were developed [64,65,66,67]. Because several of vinyl ester tripeptides synthesized by this group demonstrated conformational similarities with the cyclic inhibitor TMC-95A and because the cyclization restricts the conformation which could provide an increase in the potency and/or selectivity, they cyclized some of their inhibitors. They observed that the cyclization did indeed increase the affinity of the inhibitors to the β1 or β5 subunit (Figure 16) [68,69]. They also synthesized analogs with different functional groups: ketone which demonstrated lower potency than the esters [70], and α,β-unsaturated N-acylpyrrole which demonstrated to be more selective to β1 subunit [71].

### 3.7. β-Lactones

**Figure 17:**
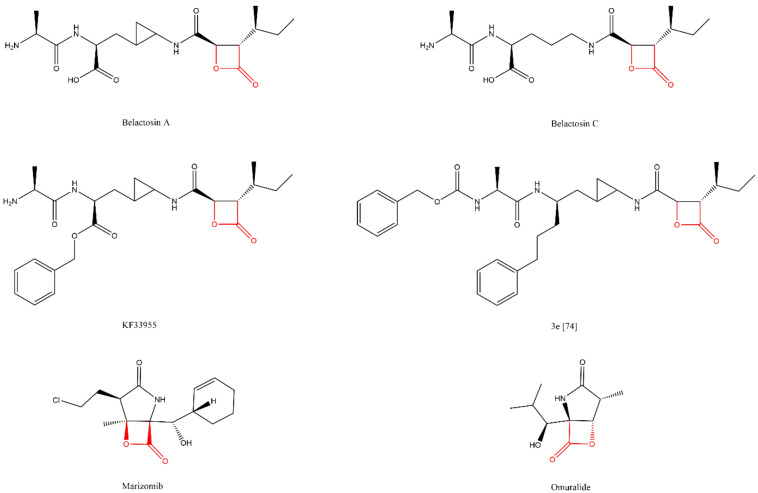
Examples of β-lactone inhibitors of 20S CP [27,36,73,74].

**Figure 18:**
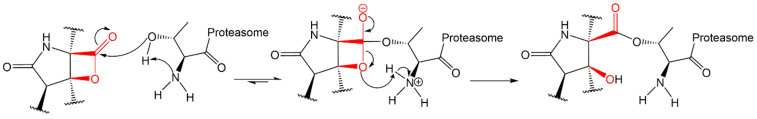
Mechanism of proteasome inhibition by β-lactone. The hydroxyl group from Thr1 reacts with the carbonyl group from the inhibitor, inducing the opening of the ring and acylation of hydroxyl from Thr1 [27,29].

Lactacystin (a metabolite from Streptomyces gram-positive) was the first natural non-peptide-like proteasome inhibitor to be found in nature, and it bears a β-lactone moiety. In vivo, it acts as a prodrug which is hydrolyzed at neutral pH into clasto-lactacystin-β-lactone (also called omuralide) (Figure 17), which can cross the plasma membranes of mammalian cells (whereas the lactacystin form cannot) and it is covalently and irreversibly bound to the β5 subunit’s Thr1, resulting in the opening of the β-lactone ring and acylation of the hydroxyl group in Thr1 (Figure 18). Omuralide does not inhibit various serine and cysteine proteases, except for cathepsin A and cytosolic tripeptidyl peptidase II [27,28,61].

In 2000, belactosin A and C were isolated from *Streptomyces* sp. by Asai et al. [72] (Figure 17). These compounds exhibited antitumor activity attributed to the inhibition of proteasome activity. Additionally, with the goal of increasing the potency of belactosin A, a benzyl group was introduced (KF33955, Figure 17) [73]. Other derivatives were synthesized in 2013 by Kawamura et al. [74], who identified the 3e derivative (Figure 17) as an inhibitor comparable to bortezomib (IC_50_ value of 5.7 nM for the β5 subunit). Belactosin C analogs of the boronate inhibitors class were synthesized with the purpose of developing reversible inhibitors [75]. However, the most potent boronate inhibitor developed exhibited a value of IC_50_ for the β5 subunit of 20S CP, 10-fold than bortezomib’s value (IC_50_ = 280 nM).

Marizomib (also named salinosporamide A or NPI-0052) (Figure 17) is a secondary metabolite of the marine actinomycete *Salinispora tropica*. This is the only non-peptidic proteasome inhibitor for which the Committee for Orphan Medicinal Products has issued a positive opinion regarding the orphan drug designation for the treatment of MM (2014) and for the treatment of glioma (2018), because marizomib crosses the blood-brain barrier [76,77]. According to the Triphase Accelerator Corporation, the orphan drug designation was also granted by the FDA, for the treatment of glioblastoma and MM [78,79,80]. It is an irreversible proteasome inhibitor whose carbonyl group interacts with Thr1′s hydroxyl group (it inhibits the three catalytic subunits of the 20S CP quickly and for a long period of time). Although it is orally bioavailable [36], all related (completed and ongoing) clinical studies have reported the drug administration as intravenous [81,82,83,84,85,86,87,88,89,90,91]. Marizomib was first tested in a phase I clinical trial conducted in patients with advanced solid tumor malignancies or refractory lymphoma whose disease had progressed after standard treatment [91]. Afterwards, two studies of phases I and II were conducted to evaluate the safety, pharmacokinetics and pharmacodynamics of escalating maximum tolerated and recommended doses of marizomib (and low dose dexamethasone) in patients with advanced malignancies including solid tumors, lymphomas, leukemias and MM (one of the studies only analyzed MM). These studies demonstrated that marizomib does not induce severe peripheral neuropathy or hematologic toxicity associated with bortezomib and carfilzomib, and it was verified that it is well-tolerated in heavily pretreated relapsed and/or refractory MM patients [82,84,92]. A phase I clinical trial has been completed to assess marizomib in combination with the histone deacetylase inhibitor vorinostat, in patients with melanoma, non-small cell lung cancer, pancreatic cancer or lymphoma [85]. This study demonstrated that this combination therapy is feasible and well-tolerated. Albeit confirmed responses were not reported, 61% of the evaluable patients reported a stable disease and 39% had decreases in tumor measurements (up to 25%) [93]. In a phase I clinical trial in patients with relapsed/refractory MM, a combination of marizomib, pomalidomide and low-dose dexamethasone demonstrated that this combination is well tolerated and promising in heavily pre-treated patients, including those who were refractory to prior treatment with carfilzomib, bortezomib and/or lenalidomide, and patients with high-risk cytogenetics (17p deletion and/or 4:14 chromosome translocation) [86,94,95]. The safety and preliminary efficacy of marizomib, alone or in combination with bevacizumab, were evaluated in patients with recurrent glioblastoma in a phase I/II clinical trial. This study demonstrated that marizomib is safe, as monotherapy or in combination with bevacizumab, for patients with recurrent glioblastoma. However, it did not show a benefit to patients from the addition of marizomib to bevacizumab. Marizomib was also shown to inhibit the proteolytic activity of all three subunits, with repeated dosing, at all doses assessed [87,96]. The detailed results from a completed phase I clinical trial to evaluate the combination of marizomib with Optune^TM^, temozolomide and radiotherapy in patients with newly diagnosed WHO Grade IV malignant glioma are expected [88,97]. Three studies are ongoing, but not yet recruiting participants: (1) a phase III trial to evaluate marizomib in combination with standard temozolomide-based radiochemotherapy versus standard temozolomide-based radiochemotherapy alone in patients with newly diagnosed glioblastoma; (2) a phase II clinical trial to evaluate nanoparticle albumin-bound rapamycin as a single agent or combined with standard therapies (including marizomib) in bevacizumab-naïve subjects with progressive high grade glioma following prior therapy and subjects with newly diagnosed glioblastoma; and (3) a phase I study to evaluate the safety, tolerability, pharmacokinetic parameters and preliminary efficacy of the drugs marizomib and panobinostat in pediatric patients with diffuse intrinsic pontine glioma [81,83,89]. A phase II study to evaluate the efficacy of treatment with marizomib for recurrent low-grade and anaplastic supratentorial, infratentorial was terminated because the pharmaceutical company leading the study closed their program evaluating marizomib [90]. A phase II study will be carried out to evaluate the combination of marizomib, more pomalidomide and dexamethasone in patients with relapsed/refractory MM patients and patients with central nervous system involvement [98].

### 3.8. Syrbactins

**Figure 19:**
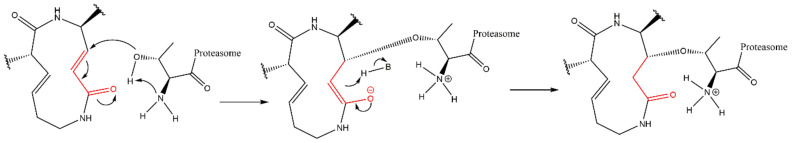
Mechanism of proteasome inhibition by syrbactin inhibitor, via a Michael-type 1,4-addition [29].

Syrbactins are a highly potent class of 20S CP inhibitors that consist of a 12-membered lactam, with an α,β-unsaturated amide system which reacts irreversibly with the hydroxyl from Thr1, through a Michael-type 1,4-addition (Figure 19).

This class of inhibitors is comprised of structurally related families of natural products, distinct in their own lactam macrocyclic systems and exocyclic chains (namely in the presence of urea): syringolins and glidobactins.

**Figure 20:**
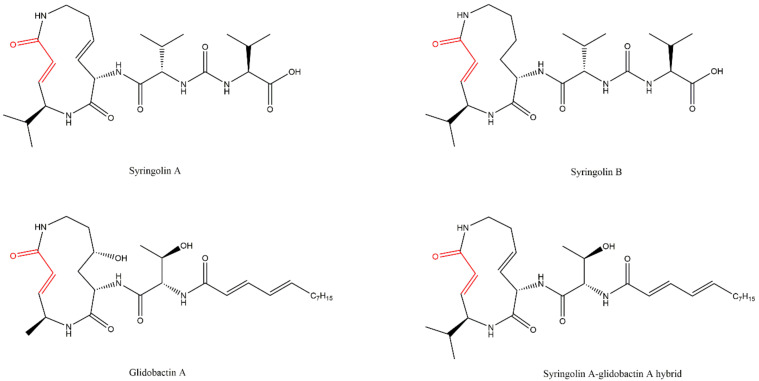
Examples of syrbactin inhibitors [99,103].

Syringolins A and B (Figure 20), produced by strains of the vegetal pathogen *Pseudomonas syringae pv. Syringae*, and the glidobactin A, isolated from various bacteria, are natural inhibitors that belong to this class.

Syringolin A proved to be a more potent inhibitor than syringolin B, being able to inhibit irreversibly the three catalytic subunits of the eukaryotic 20S CP, and showed anticancer activity, pointing to the existence of apoptosis in human neuroblastoma and ovarian cancer cells. Various analogs were also synthesized with the help of their total synthesis.

Glidobactin A, isolated from the bacterial strain *Polyangium brachsporum*, was described as an antitumoral drug, and its cellular target (20S CP) was only identified 20 years later, because of its similar structure to that of syringolin A. It showed an inhibitory activity 15-fold higher than syringolin A for the β2 and β5 subunits, but there was no evidence that it could inhibit the β1 subunit [99,100,101,102]. The hybrid inhibitor syringolin A-glidobactin A (Figure 20) showcased inhibitory activity to the β1 subunit [103].

## 4. Resistance Mechanisms to Proteasome Inhibitors (PIs)

Acquired or innate PI resistance is a major obstacle in the treatment of MM and MCL. Resistance to bortezomib is particularly relevant since its therapeutic effect heavily depends on interpatient variability: only around 35% of patients with MM respond to bortezomib therapy [104]; the newly diagnosed patients did not achieve a partial or better response [39] and the ability of patients, who previously demonstrated sensitivity to bortezomib, to return to positive responses to bortezomib ranges between 31% and 60% [105], emerging as a limitation to continued clinical use.

As a strategy to partially overcome bortezomib resistance, irreversible inhibitors, such as carfilzomib (or more potent ones) were used because the prolonged 20S CP inhibition would induce a less resistant antitumor response and several studies have shown less pronounced cross-resistance compared to bortezomib [39,106,107,108,109]. Two phase II clinical trials demonstrated that carfilzomib had inhibitory activity in patients with relapsed and/or refractory MM who had already received bortezomib treatment [110,111]. A phase I/II clinical trial demonstrated that replacing bortezomib with carfilzomib, in patients with MM, who failed to bortezomib-containing combination regimens is safe and can be effective [112]. In 2018 a similar phase I/II clinical trial was initiated to evaluate the efficacy and safety of ixazomib as a replacement for bortezomib or carfilzomib among MM patients who are non-responsive to proteasome inhibitor-containing combination regimens [113]. Preliminary results show that the replacement of bortezomib or carfilzomib with ixazomib rarely leads to responses among the participants [114]. Marizomib is an irreversible inhibitor and inhibits all three catalytic subunits of 20S CP, and therefore it can help overcome bortezomib and carfilzomib resistance, associated with the upregulation or mutation of the β5 subunit [115].

Different mechanisms have been suggested to lead to drug resistance e.g., mutations and overexpression of proteasome subunit β5 [116], alterations in genes associated with stress response such as heat shock proteins [117], and up-regulation of cell survival pathways such as the insulin-like growth factor 1/insulin-like growth factor type 1 receptor axis (IGF-1/IGF-1R) [118] and peptidylprolyl isomerase A (PPIA) [119].

Next, we describe some of the main mechanisms associated with innate and acquired resistance to PIs and some of the approaches developed to overcome this therapeutic drawback, namely by using combination therapies.

### 4.1. Innate Resistance

Innate resistance, also known as inherent [106] or intrinsic resistance [120,121], is defined by the ability of the cell to survive in the presence of a drug to which the cell has not been previously exposed (resistance before therapy, in this situation tumor cells that do not respond to the therapy) [121]. Researchers believe that innate resistance is mainly related to proteasome overexpression, as well as single nucleotide polymorphisms (SNPs) of the genes encoding the β subunits of the 20S CP. Resistance also arises from the tumor type and, for instance, clinical efficacy of bortezomib monotherapy in solid tumors is lower than in hematological malignant tumors [106]. However, the innate resistance of patients to PIs can also be influenced by gender, age, environmental factors, therapeutics previously performed and the stage of pathology [122].

#### 4.1.1. Proteasome Overactivation and Overexpression

Regarding the innate overexpression of the β5 subunit, the study conducted by Li et al. [123] demonstrated that the K562 human leukemia cell line overexpresses the β5 subunit and is more resistant to bortezomib when compared with other cell lines of leukemia (OCI-AML2) and myeloma (MY5).

In the following year, Shuqing et al. [124] performed a small study with three patients with MM where cancer cell samples were collected from the bone marrow pre-treatment (before the patients were treated for four weeks with a therapy involving bortezomib) and post-treatment if a complete response was not achieved after six cycles of treatment; and bone marrow mononuclear cells were collected from healthy volunteers (control group). They analyzed the mRNA expression levels of the β5 subunit gene and carried out DNA sequencing to identify possible mutations. Two patients with MM of IgG λ subtype and stage II and III (according to the International Staging System) achieved complete response after one cycle of treatment. The third patient with MM of light chain λ subtype and stage III did not achieve complete response after eight cycles of treatment. It was found that there were no statistically significant differences in the mRNA levels of the β5 subunit gene between pre-treatment samples from the three patients and samples from the control group. However, after six cycles of treatment, the third patient had 5-fold higher mRNA expression of this subunit when compared to the control group and 5-fold higher expression than before treatment (acquired resistance). DNA sequencing did not identify mutations in the gene encoding the β5 subunit in the pre-treatment samples from the three patients, in the samples from the control group and in the post-treatment samples collected from the third patient after six cycles of treatment.

In 2012, de Wilt et al. [120] demonstrated that different human non-small cell lung cancer cells (H460, A549 and SW1573 cell lines) show differential sensitivity to bortezomib with IC_50_ values of 12.6, 8.7 and 1.7 nM for H460, A549 and SW1573, respectively. These differences can be pinned down to the different proteasome activities in these cells, while expression levels remained the same. H460 cells presented significantly more (3 and 4.5-fold) activity of β1 and β5 subunits compared to SW1573 cells, whereas A549 cells showed intermediate activity of these β subunits.

Niewerth et al. [125] found that acute myeloid leukemia patients expressed relatively higher levels of β1, β2 and β5 than acute lymphoblastic leukemia patients. This was associated with lower sensitivity to bortezomib, carfilzomib and oprozomib (separately) in the former. Additionally, overexpression of β1 and β5 subunits was also correlated to the lower sensitivity to carfilzomib and bortezomib. The researchers also propose that the underexpression of the immunoproteasome subunits, alongside the overexpression of the constitutive proteasome, also plays a role in the innate resistance. It is important to note that in this research the patient samples did not show mutations in the β5 subunit of the 20S CP, which corroborates the idea that these mutations are related to prolonged exposure to bortezomib (acquired resistance).

In 2016, Niewerth et al. [126] reported that acute myeloid leukemia (AML) cells did not differ from pre-B or T subtypes of acute lymphoblastic leukemia (ALL) cells in β1 subunit expression, but the AML and T subtype of ALL cells showed a higher β5 subunit expression than pre-B subtype of ALL cells.

#### 4.1.2. Single Nucleotide Polymorphisms (SNPs) of the Genes Encoding the Three Catalytic Subunits β of the 20S CP

The SNPs of the genes encoding the β subunits of the 20S CP may contribute to individual variability in disease pathophysiology and/or in the response to 20S CP inhibitor therapy [122].

Wang et al. [122] performed a study that included 240 DNA samples (60 participants from four ethnic groups: African Americans, Caucasian Americans, Han Chinese Americans and Mexican Americans) and re-sequenced genes encoding the β1, β2 and β5 subunits. They identified a series of polymorphisms which included two nonsynonymous SNPs in the gene encoding the β1 subunit and three nonsynonymous SNPs in the gene encoding the β5 subunit. None of the nonsynonymous SNPs identified changed amino acids located within the catalytic sites of the subunits. Allele frequencies for the nonsynonymous SNPs differed widely among ethnic groups; for example, the Arg24Cys polymorphism (substitution of arginine at position 24 for cysteine) of the gene encoding the β5 subunit was present in all groups, except in the Han Chinese American group; the Try212Cys polymorphism of the gene encoding the β5 subunit was identified only in a sample from a Mexican American participant, and the Val238Met polymorphism of the gene encoding the β5 subunit was observed in a sample obtained from a Han Chinese American participant. Through cytotoxicity studies, they verified that the SNPs of the gene encoding the β5 subunit did not significantly affect the proteasome activity and the inhibition or cytotoxicity induced by MG262 boronate inhibitor. However, they did not rule out the possibility that these polymorphisms alter the degradation of specific protein-substrates, and that they might induce different results when another 20S CP inhibitor is used. Additionally, 79 DNA samples obtained from 61 patients with MM who had previously been treated with bortezomib were analyzed, and they did not detect a significant association between SNPs in the gene encoding the β5 subunit and response to bortezomib therapy, due to the reduced number of samples and the heterogeneity of the disease. However, SNPs at nucleotide 1042 can influence expression of the gene encoding the β5 subunit. In 2013, Lü and Wang described that the Arg24Cys polymorphism in the gene encoding the β5 subunit is five times more frequent in patients with MM than in the general population, as previously reported [122,127].

In 2012, Lichter et al. [128] performed a study with an objective to investigate a possible association between the variations in the genes encoding the β1, β5 and β6 subunits and the resistance to bortezomib treatment in patients with relapsed MM. They sequenced genes encoding the β1, β5 and β6 subunits of tumor DNA pre- and post-treatment samples from patients who participated in phase III of the Assessment of Proteasome Inhibition for Extending Remissions (APEX) trial of single-agent bortezomib versus high-dose dexamethasone for the treatment of relapsed MM. Through a comparison between allelic and genotype frequency of nonsynonymous SNPs in pre- and post-treatment, it was observed that the samples did not differ significantly from a weighted average of European population data from the National Center for Biotechnology Information SNP database, suggesting that nonsynonymous variants in the genes encoding the β subunits are not specifically selected in MM. In addition, no unique nonsynonymous replacements were observed in post-treatment samples, and they registered that recurrent variants occurred at similar frequencies in pre- and post-treatment samples, suggesting that variations in the genes encoding the β subunits do not arise during treatment (acquired resistance) and that these variants are more prone to represent naturally occurring germline single nucleotide polymorphism. One variant (a C/G substitution resulting in a Ser112Arg change approximately 7 Å away from the bortezomib binding pocket) located in gene encoding the β5 subunit was identified in pre-treatment sample from a patient who had achieved a partial response to bortezomib. This study included samples post-treatment from 10 patients who were relatively insensitive to bortezomib monotherapy (best response of minimal response, stable disease or progressive disease) and from six patients who achieved a confirmed partial response but subsequently relapsed on study before sample collection. In these cases, resistance to bortezomib monotherapy was independent of variants in the genes encoding the β subunits, thus excluding the hypothesis that the acquired resistance to bortezomib that develops in some patients with MM is due to variants in the genes encoding the 20S CP subunits. They also did not find associations between the frequencies of the SNPs in the genes encoding the β subunits in the pre- and post-treatment sample set and the subsequent patient response to bortezomib or dexamethasone treatment. Nevertheless, the small sample size limited this study. The rs12717 SNP in the gene encoding β6 subunit (a C31G substitution resulting in a Pro111Ala change) was associated with a relatively progression-free survival benefit in relapsed follicular lymphoma treated with bortezomib-rituximab versus rituximab [129]. However, in the study by Lichter et al., this benefit was not observed with bortezomib or dexamethasone monotherapy. The β6 subunit has no known direct proteolytic activity, but it has been proposed that it contributes to the assembly and stability of the two proteasome heptameric β rings and to the formation of the proteolytic environment on their inner surface. Therefore, this polymorphism may influence the efficacy of bortezomib therapy [130]. Vargas et al. [130] reported that carrying the rs12717 SNP in the gene encoding β6 subunit is predictive for suboptimal response with bortezomib treatment in myeloma cells, which could be explained by less active proteasomes which are less sensitive to bortezomib (caspase- and trypsin-like activity from GG individuals).

### 4.2. Acquired Resistance

Acquired resistance implies that the cells develop survival mechanisms after drug exposure, arising during treatment [121], generally by targeted overexpression of 20S CP subunits, genetic mutations and upregulation of channel proteins/transporters which mediate removal of 20S CP inhibitors from the cells, but also through the activation of anti-apoptotic mechanisms that involve upregulation of heat shock proteins (HSP) (with proteasome inhibition there is overexpression of the proteins HSP27, HSP70, HSP72, HSP90), altered expression of apoptosis-related proteins (e.g., BCL-2 and p27), increased antioxidant levels, growth-related proteins (e.g., interleucine-6 and the insulin-like growth factor 1), protein kinase B activation and upregulated autophagy [106,131,132,133,134,135,136,137].

#### 4.2.1. 20S CP β Subunits Overexpression

It is argued that acquired resistance is also related to the overexpression of β subunits of the 20S CP. Some studies regarding the resistance to bortezomib (by repeated drug exposure) mediated by this overexpression are reported in Table 1.

Interestingly, in their study, Suzuki et al. [107] verified that, for bortezomib-resistant HT-29 adenocarcinoma cells in the absence of bortezomib, a reduction in the β5 level overexpressed occurred.

Diverse studies [116,120,138,140] identified an increase in 20S CP levels in bortezomib-resistant cells, which indicates that there was not only an increase in β5 subunit level. Some studies indicate that the upregulation of β subunits contributes minimally to cellular resistance to 20S CP inhibitors and free β subunits, which are catalytically inactive and subsequently, do not present capacity to bind to inhibitors, unless they are assembled into functional proteasomes [3].

It is important to correlate the level of resistance detected in a study with the corresponding extent of β5-subunit expression, but also to evaluate mRNA transcriptional levels and protein levels of the β subunits, for a better clarification of 20S CP inhibitors resistance mechanisms. Oerlemans et al. [116], and Franke et al. [140] concluded in their studies that while it is possible that the level of mRNA of the gene encoding the β5 subunit remains unchanged in cases of cell resistance, a not substantiated increase in β5 subunit level may be observed, suggesting that additional post-transcriptional mechanisms contribute to resistance.

#### 4.2.2. β5. Subunit Mutation

Another acquired resistance mechanism to the 20S CP that is suggested is mutation. Actually, mutations in the gene encoding for the β5 subunit occur after treatment with the inhibitors. Some β5 subunit mutations are reported in Table 2.

Cross-resistance to other 20S CP inhibitors was registered in bortezomib-resistant cell lines [120,141,144]. However, Suzuki et al. [107] suggested that inhibition of β5 subunit by irreversible inhibitors (e.g., carfilzomib) is unaffected by the Cys63Phe mutation.

Huber et al. [131] registered a high attenuation of the chymotrypsin-like activity in the yeast 20S CP for Ala49Ser, Ala49Thr, Ala49Thr_Ala50Val, Ala49Val, Ala50Val, Cys52Phe, Met45Ala, Met45Thr, Met45Val and Met45Ile mutations, except for Cys63Phe mutation, compared with the wild-type yeast 20S CP. Additionally, some studies performed crystallographic analysis of the impact of mutation in the gene encoding the β5 subunit [107,127,131,133,140,145,146]. Generally, mutations observed at Thr21, Ala49 and Ala50 may disrupt H-bonding and, subsequently, directly influence bortezomib binding to β5 subunits, whereas Cys52 and Met45 mutations are indirectly involved in bortezomib binding because they are in close proximity to the bortezomib-binding pocket in β5 subunits [127,131,133,145,146].

Carfilzomib could overcome mutation-mediated resistance better than bortezomib, which may result from irreversible binding as well as from its tetrapeptide moiety, which significantly improves anchoring in the β5 substrate binding channel compared with the dipeptide bortezomib [131].

Suzuki et al. [107] using the software MOE (Molecular Operating Environment) analyzed the Cys63Phe mutation in the crystal structures of the yeast proteasome α5/β5/β6 subunits unbound or bound to bortezomib or epoxomicin. They observed that Cys63 is housed in the same helix Ala49/50, which are residues critical for bortezomib binding, and the Cys63Phe mutation leads to a shift in the angle of the helix with respect to the active site. This shift is much more significant in the inhibitor-bound forms than in the unbound forms and it did not alter the orientation of epoxomicin but resulted instead in a twist in the orientation of bortezomib, affecting the binding of bortezomib to the β5 subunit. The crystallographic analysis by Huber et al. [131] revealed that this mutation did not induce structural peculiarities, and together with biochemical analysis they concluded that the mutation Cys63Phe does not confer bortezomib resistance in yeast 20S CP.

Niewerth et al. [144] reported that in the β5 active site, the Met45 residue facilitates the marizomib binding by hydrophobic interactions, and so Met45 mutations restrict this interaction.

Some studies reported that there are tumor cells from bortezomib resistant patients which do not evidence mutation in the gene encoding the β5 subunit of the 20S CP [124,136,147,148]. This clinical evidence can be related to the time of cellular exposure to the inhibitor and to its concentration; several studies indicate that the overexpression of proteasomal activity may constitute a primary mechanism as a cellular response to the treatment of bortezomib, which precedes the mutation acquisition after prolonged inhibitor exposure, but there may also exist other multifactorial mechanisms which contribute to acquired cellular resistance [133,138].

#### 4.2.3. Alteration of the Expression Levels of Transporters which Mediate Efflux of 20S CP Inhibitors

Even though this is an indirect mechanism of resistance, it is nonetheless clinically relevant. Drug transporters can be categorized into influx and efflux transporters. The efflux transporters are frequently related with anticancer drug resistance, usually mediated by the ATP-binding cassette (ABC) superfamily, which includes P-gp (P-glycoprotein), the MRP (Multidrug Resistance Protein) family and BCRP (Brest Cancer Resistance Protein) [149].

Information about bortezomib interaction with drug transporters is far from complete and contradicting evidence exists. However, several studies revealed P-gp overexpression does not markedly induce resistance to bortezomib, nor do BCRP, MRP1–9 and LRP transporters [116,120,138,139,150,151,152]. On the other hand, O’Connor et al. [153] suggest that bortezomib is a substrate and a weak inhibitor of P-gp efflux transporters and that bortezomib activity against proteasome is affected by high cellular overexpression of these transporters. In addition, they have observed that cellular treatment with bortezomib can induce decreased expression of these efflux transporters.

For ixazomib, EMA and the FDA indicated that it has a low affinity with P-gp, and it does not interact with P-gp, BCRP and MRP2 transporters [4,7]. Carfilzomib is classed by the EMA and FDA as a substrate of P-gp but not BCRP [5,8].

There are studies which reported an upregulation of P-gp transporters as a resistance mechanism for 20S CP inhibitors (unlikely to influence bortezomib and ixazomib). Some studies reporting resistance to 20S CP inhibitors mediated by P-gp overexpression are listed in Table 3. The study by Verbrugge et al. [141] indicated which MRP1–5 and BCRP transporters do not contribute to carfilzomib and oprozomib efflux.

Rumpold et al. [158] suggested that resistance to 20S CP inhibitors mediated by P-gp transporters may not be applicable for all inhibitors, even those of the same class, possibly because they may have far different kinetics with respect to their P-gp affinity. They registered a significant reduction in proteasome activity in the K562/Dox-MM cell line of myeloid leukemia (which exhibits a stable P-gp knockdown) compared to the K562/Dox-H1 cell line (exhibiting an overexpression of P-gp transporters) after exposure to PS273. On the contrary, after exposure to bortezomib, no significant differences in proteasome activity were identified between the two cell lines.

Besse et al. [154] (first study of Table 4) verified that marizomib activity was almost independent from the level of P-gp with the weakest interaction with this transporter. They also reported that bortezomib and ixazomib showed moderately low degrees of P-gp interactions, delanzomib was a considerably strong P-gp substrate, and that cytotoxicity of oprozomib and carfilzomib were very sensitive to P-gp overexpression.

Clemens et al. [159] investigated the capacity of bortezomib, carfilzomib and ixazomib to alter the expression and/or activity of efflux transporters (BCRP, MRP1, MRP2 and P-gp). They registered that none of the PIs tested significantly induced or repressed any of the drug transporters’ genes in the LS180 human colon adenocarcinoma cell line, following a period of exposition of 4 days. However, for myeloma cells, ixazomib caused certain changes: it induced the expression of gene encoding the P-gp in Karpas-620 cells, of gene encoding the MRP1 in L363 cells and of gene encoding the BCRP in KMM-1, LP-1 and U266 cells, and suppressed this gene in Karpas-620 cells. Carfilzomib suppressed mRNA’s expression of the gene encoding the P-gp in LP-1 cells.

The combination with P-gp inhibitors is suggested to overcome 20S CP resistance caused by overexpression of MDR transporters. However, considering the role of P-gp is also important to prevent resistant phenotypes before treatment with 20S CP inhibitors, such as through the use of efflux analysis to determine whether high expression of P-gp is predictive of poor clinical responses in patients treated with 20S CP inhibitors, namely carfilzomib [160].

Ao et al. [155] predicted that carfilzomib would be a good molecule lead in developing resistance-reversing agents, and they synthesized a small library of 10 peptide analogs based on the peptide backbone structure of carfilzomib. They screened these molecules for their activity to restore carfilzomib sensitivity when co-treated with carfilzomib and they found that compounds as small as dipeptides are sufficient to restore carfilzomib sensitivity.

The study of Besse et al. [154] reveals that resistance to 20S CP inhibitors (carfilzomib and oprozomib) mediated by P-gp transporters overexpressed can be overcome with nelfinavir and lopinavir (human immune deficiency virus protease inhibitors). They identified nelfinavir and lopinavir as potent functional modulators of P-gp, most likely via modulation of the mitochondria permeability transition pore. In the absence of P-gp, they verified that nelfinavir and lopinavir also retained sizable carfilzomib-sensitizing activity, and they concluded that these drugs target additional molecules that substantially contribute to 20S CP inhibitors resistance.

## 5. Combination Therapy to Overcome Resistance to Proteasome Inhibitors (PIs)

Combination therapy presents itself as an important strategy to potentially overcome the effects of resistance to PIs. The main classes of agents that have been used in combination with 20S CP inhibitors are corticosteroids (e.g., dexamethasone and prednisone), cytotoxic agents (e.g., the bendamustine, melphalan and cyclophosphamide alkylating agents and doxorubicin anthracycline), immunomodulatory agents (e.g., lenalidomide, pomalidomide and thalidomide) and inhibitors of histone deacetylases (e.g., panobinostat, vorinostat, belinostat and romidepsin).

Different combination therapies were studied as a strategy to overcome 20S CP inhibitors resistance because different drugs can interact in an additive or synergistic mechanism to induce apoptosis [106]. For example, inhibitors of histone deacetylases (HDACs) and 20S CP inhibitors interfere with vital pathways to proliferation and survival, with some common pathways (e.g., upregulation of pro-apoptotic proteins), but also with complementary pathways that may underlie synergistic effects. The most well-characterized synergistic model between these two types of inhibitors is the dual inhibition of protein degradation pathways: the proteasome pathway and the lysosomal pathway (ubiquitinated proteins aggregate in aggresome upon proteasome failure, and they are degraded by the autophagy-lysosome pathway) [161]. HDAC6 has an essential role in protein degradation via the autophagy-lysosome pathway [162]. HDAC6 inhibitors increase the acetylation of α-tubulin and, consequently, block aggresome formation and the degradation of proteins by the autophagy-lysosome pathway. These molecules are considered very promising because they may complement proteasome inhibition, which would explain the synergistic effect between HDAC inhibitors and 20S CP inhibitors [161,162,163].

**Figure 21:**
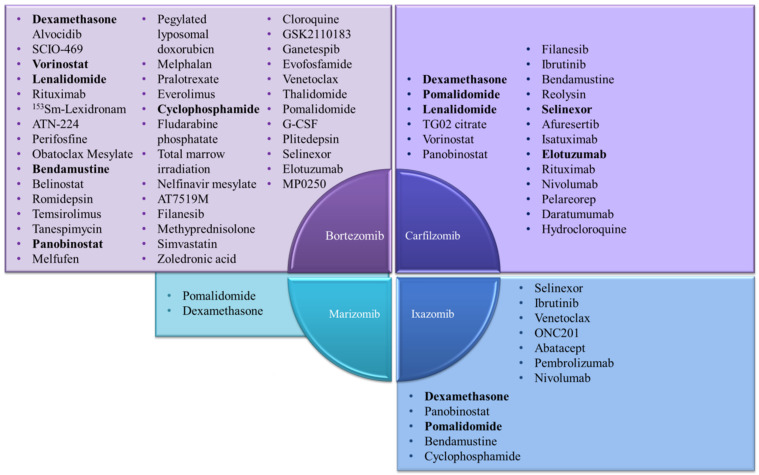
Combination therapies with 20S CP inhibitors in clinical trials, involving patients with relapsed and/or refractory disease, and who were previously treated with a 20S CP inhibitor (bold drugs are most frequently involved in combination therapy). Clinical trials were selected from ClinicalTrials (https://clinicaltrials.gov/ accessed on 22 February 2022), entering the search filter “bortezomib”, “carfilzomib”, “ixazomib”, “oprozomib”, “delanzomib” and “marizomib” in “other terms” fields, and sorted by study description, inclusion and exclusion criteria and results.

Some clinical trials of combination therapies with 20S CP inhibitors that can overcome their resistance are reported in Table 4 and Figure 21. (Additional data on clinical trials are reported in the Supplementary Materials Table S1).

Furthermore, some patents cover the application of combination therapies capable of reducing or overcoming 20S CP inhibitors resistance. These patents contain agents that lead to increased expression or activity levels of the 19S RP [164], 19S RP inhibitors [165,166], galectin-3C (an N-terminally truncated form of the human carbohydrate binding protein, galectin-3) [167], rapamycin and analogs which are inhibitors of the catalytic core proteasome involving allosteric interactions [168], the HDAC romidepsin [169], cytotoxic agents [170], MEK inhibitors [171] and agents which induce the formation of reactive oxygen species and oxidative damage [172].

In 2019, XPOVIO^®^ (selinexor), the first-in-class, oral selective inhibitor of nuclear export (SINE) compound was approved by the FDA in combination with dexamethasone for the treatment of relapsed or refractory MM patients who have received at least four prior therapies and whose disease is refractory to at least two PIs, at least two immunomodulatory agents, and an anti-CD38 monoclonal antibody, based on results of STORM clinical trial [173]. In early 2020, the FDA accepted the filing of an application seeking accelerated approval for selinexor for the treatment of relapsed or refractory diffuse large B-cell lymphoma in patients who have received at least two prior therapies [174]. Selinexor is a covalent reversible inhibitor of the nuclear export protein XPO1/CRM1, one of the seven human exportins that are well-described as a nuclear export protein overexpressed in diverse cancer cells, inducing cellular deregulation with excessive nuclear export of tumor suppressor proteins, of growth regulatory proteins and of mRNAs of oncogenic proteins such as c-MYC, BCL-2, and cyclin D1, allowing cancer cells to grow and divide uncontrollably, and leading to the restoring of tumor suppressor proteins and cell-cycle regulators in the cell nucleus which is necessary to identify DNA damage, to help signal for repair mechanisms, or if the damage is too severe, for apoptosis. Due to the synergistic effect of selinexor combined with a 20S CP inhibitors in pre-clinical trials in MM cells and animal models, selinexor is included in combination therapies with the 3 20S CP inhibitors approved in clinical trials [173,175,176].

A phase I study of the combination of selinexor with carfilzomib and dexamethasone in patients with relapsed or relapsed/refractory MM was initiated in 2014 and it is currently recruiting patients [177]. A phase I dose-escalation trial of twice-weekly selinexor in combination with carfilzomib and dexamethasone showed that participants achieved disease control after one cycle, with an overall response rate of 38% and clinical benefit rate of 67%. The overall response rate (ORR) was 48%; 24%, 10% and 14% of the participants achieved a minimal response, stable disease, and progressive disease, respectively. The ORR for carfilzomib-refractive patients was 62% and 15% achieved a minimal response. The results indicated that selinexor could be a possible subsequent therapy, mainly as a therapy to at least transiently overcome resistance [176]. Additionally, a phase II study to compare the efficacy of carfilzomib plus low-dose dexamethasone with and without selinexor in patients with relapsed/refractory MM. However, this study has been withdrawn prior to enrollment [178]. A phase Ib/II study was also carried out to evaluate six combination therapies (including three or four drugs among selinexor, bortezomib, dexamethasone, pomalidomide, carfilzomib, daratumumab and lenalidomide), in participants with relapsed/refractory MM after at least one previous line of therapy, but who were not refractory to bortezomib in their most recent line of therapy [179]. Clinical results reported in 2018 support pre-clinical evidence that selinexor re-sensitizes and overcomes resistance to PIs. In this clinical trial testing, selinexor plus bortezomib plus dexamethasone, proteasome-inhibitors-refractory participants had an ORR of 43% [175].

Additional phase I studies with selinexor-containing combinations were also initiated but have no results yet, namely to study selinexor plus ixazomib plus low dose of dexamethasone in refractory MM [180]. Phase II and III studies testing selinexor plus bortezomib plus dexamethasone for refractory MM [181,182,183,184,185,186] were also started, and additional phase II trials with selinexor plus bortezomib plus dexamethasone plus daratumumab [187] and selinexor plus carfilzomib [188,189] in refractory MM were also registered.

## 6. Final Considerations and Future Perspectives

The deregulation of the ubiquitin–proteasome pathway is associated with diverse diseases, namely, neoplastic ones. The use of PIs is a therapy that has received attention from the pharmaceutical industry. Different classes and several inhibitors have been developed to date. However, only three were approved by the FDA and EMA: bortezomib, carfilzomib and ixazomib.

A major limitation for the clinical success of these inhibitors is innate and/or acquired resistance, associated, for example, with proteasomal overactivation and overexpression, single nucleotide polymorphisms of the genes encoding the catalytic subunits β of the 20S proteasome, β5 subunit mutations and upregulation of the expression levels of the transporters which mediate the efflux of these inhibitors from the cells.

To overcome resistance, a relevant strategy is the development of combination therapy with 20S CP and other agents such as inhibitors of efflux transporters (P-gp), corticosteroids, cytotoxic agents, immunomodulatory agents and inhibitors of histone deacetylases.

However, it is also important to prevent resistant states before treatment with 20S CP inhibitors. Some methods to determine whether a patient will be responsive or non-responsive to a proteasome inhibition therapeutical agent have been patented and comprise the determination of key predictive markers and the analysis of a gene expression panel [190,191].

Multi-targeting could be another strategy that can reduce the number of drugs within the traditional cocktails, such as the RTS-V5, the first-in-class dual HDAC-proteasome ligand [192].

The development and combination (with other anti-cancer agents) of PIs outlined here still have a long but very promising path ahead.

## Figures and Tables

**Table 1:** Examples of studies of resistance to bortezomib (by continued exposure to inhibitor) mediated by 20S CP β5 subunit overexpression.

Bortezomib-Resistant Cell Lines	Observations (Alteration of the Expression Levels of Proteasomal Subunits)	Ref.
Human Namalwa Burkitt lymphoma	Proteolytic activities increased. Increased expression of the proteolytic subunits α, β1, β2, β4, β5 and β6, indicating that the cells abundantly express and assemble complete 20S complexes.	[138]
Jurkat B1 and B5 of lymphoblastic lymphoma/leukemia (highly resistant)	mRNA of the gene encoding the β5 subunit levels overexpressed, amplification of this gene and increased chymotrypsin-like activity.	[139]
Jurkat B2 of lymphoblastic lymphoma/leukemia (slightly resistant)	No significant differences were registered in comparison with parental cells.	[139]
THP-1 of human myelomonocytic	β5 subunit levels overexpressed up to 60-fold (proportional to the gradually increasing concentrations of bortezomib during the stepwise selection). β1 and β2 subunit’s expression was less than 2-fold increased.	[116]

**Table 2:** Examples of mutations in the gene encoding the β5 subunit of 20S CP, in cell lines resistant to 20S CP inhibitors. (*single nucleotide polymorphism).

Mutation	Cell Line	Resistance	Observations	Refs.
Ala49Thr	THP-1 of human myelomonocytic	Bortezomib	Cross-resistance to carfilzomib and oprozomib. The siRNA-guided silencing of β5 subunit gene expression restored bortezomib sensitivity.	[116,140,141]
	Jurkat B of lymphoblastic lymphoma/leukemia	Bortezomib	Chymotrypsin-like activity did not differ significantly between mutated and non-mutated cells.	[142]
	KMS-11 and OPM-2 of multiple myeloma	Bortezomib and MG132	The artificial introduction of the mutation in the cell line KMS-11 induced a less prominent resistance.	[143]
	8226 of multiple myeloma	Bortezomib		[140]
	CCRF-CEM of leukemia	Bortezomib	Cross-resistance to marizomib.	[140,144]
	H460 e A549 of non-small cell lung cancer human	Bortezomib	Cross-resistance to carfilzomib and MG132.	[120]
Ala49Val	Jurkat B of lymphoblastic lymphoma/leukemia	Bortezomib	Chymotrypsin-like activity did not differ significantly between mutated and non-mutated cells.	[142]
	CCRF-CEM of leukemia	Bortezomib		[140]
Ala50Val	Jurkat B of lymphoblastic lymphoma/leukemia	Bortezomib		[142]
Arg24Cys (*)	HT-29 of adenocarcinoma	Bortezomib	Proteasome activity and sensitivity to MG262 did not change.	[107]
Cys52Phe (*)	CCRF-CEM of leukemia	Bortezomib	Cross-resistance to marizomib.	[140,144]
	SW1573 of non-small cell lung cancer human	Bortezomib	Cross-resistance to carfilzomib and MG132.	[120]
Cys63Phe	HT-29 of adenocarcinoma	Bortezomib		[107]
Met45Ile	THP-1 of human myelomonocytic	Bortezomib	Cross-resistance to carfilzomib and oprozomib.	[140,141]
Met45Val	THP-1 of human myelomonocytic	Bortezomib		[140]
	H549 of non-small cell lung cancer human	Bortezomib	Cross-resistance to carfilzomib and MG132.	[120]
	CCRF-CEM of leukemia	Marizomib	Cross-resistance to bortezomib.	[144]
Thr21Ala	8226 of multiple myeloma	Bortezomib		[140]

**Table 3:** Examples of studies that identified situations of resistance to 20S CP inhibitors mediated by P-gp efflux transporters.

Cell Line	Resistance to Inhibitor	Observations	Ref.
AMO of multiple myeloma and ARH77 of plasmoide leukemia	Carfilzomib (resistance induced by continued exposure to inhibitor)	Additionally, the influence of P-gp transporters on other inhibitors was evaluated. The ratio of IC_50_ values between the ABCB1-containing and the ABCB1-deficient AMO cells was 2.6, 7.8, 3.7, 2, 6.7 and 1.9, respectively to bortezomib, carfilzomib, delanzomib, ixazomib, oprozomib and marizomib.	[154]
CEM/VLB of T-cell leukemia with P-gp transporters overexpressed	Carfilzomib and oprozomib	Comparatively with parental CEM cells, the cells were 4.5-fold resistant to bortezomib, 23-fold resistant to oprozomib and 114-fold resistant to carfilzomib. The P-gp inhibitor P121 restored the capacity of inhibitors to inhibit chymotrypsin-like proteasome activity at inhibitory concentrations obtained with parental CEM cells.	[141]
H23 of human lung adenocarcinoma and DLD of human colon adenocarcinoma	Carfilzomib (resistance induced by continued exposure to inhibitor)	Cross-resistance to YU-101. No significant differences, compared with cells, were registered for the BCRP and MRP1–3 transporters levels. The P-gp inhibitor verapamil restored carfilzomib sensitivity of cells.	[155]
KMS11 of multiple myeloma	Epoxomicin (resistance induced by continued exposure to inhibitor)	Mutations in the gene encoding the β5 subunit of 20S CP were not identified. The verapamil restored epoxomicin sensitivity of cells.	[156]
RPMI8226.Dox40 of doxorubicin-resistant multiple myeloma with P-gp transporters overexpressed	Carfilzomib	Pre-treatment with the verapamil partially overcame the resistance to carfilzomib.	[157]

**Table 4:** Examples of clinical trials which apply combination therapies with 20S CP inhibitors. Adapted from: ClinicalTrials.gov (https://clinicaltrials.gov/ accessed on 22 February 2022).

Study (Code)	Title	Status	20S CP inhibitor	Other Drugs
NCT00401011	An Open-Label Phase I/II Study of the Safety and Efficacy of Perifosine and Bortezomib with or without Dexamethasone for Patients with Relapsed or Refractory Multiple Myeloma Previously Treated with Bortezomib	Completed	Bortezomib	Dexamethasone and perifosine
NCT01083602	A Phase II, Multi-center, Single Arm, Open Label Study of Panobinostat in Combination with Bortezomib and Dexamethasone in Patients with Relapsed and Bortezomib-refractory Multiple Myeloma	Completed	Bortezomib	Dexamethasone and panobinostat
NCT01794507	A Phase 1b Study Evaluating the Safety and Pharmacokinetics of ABT-199 in Relapsed or Refractory Multiple Myeloma Subjects Who Are Receiving Bortezomib and Dexamethasone as Their Standard Therapy	Completed	Bortezomib	Dexamethasone and venetoclax
NCT02188537	Nelfinavir as Bortezomib-sensitizing Drug in Patients with Proteasome Inhibitor-nonresponsive Myeloma. A Multicenter Phase II Trial	Completed	Bortezomib	Dexamethasone and nelfinavir
NCT04065789	Safety, Tolerability, and Efficacy of Once Weekly Carfilzomib in Combination with Daratumumab, Lenalidomide, and Dexamethasone, in Transplant-ineligible Multiple Myeloma Patients Non-responsive to a Bortezomib Based Induction	Completed	Carfilzomib	Daratumumab, lenalidomide and dexamethasone
NCT04163107	Combined Carfilzomib and Hydroxychloroquine in Patients with Relapsed/Refractory Multiple Myeloma–a Phase 1 Trial	Active, not recruiting participants	Carfilzomib	Hydroxychloro-quine and dexamethasone

## Supplementary Materials

The following supporting information can be downloaded at: https://www.mdpi.com/article/10.3390/molecules27072201/s1, Table S1. Clinical trials of combination therapies with 20S CP inhibitors, involving patients with relapsed and/or refractory disease, and who were previously treated with a 20S CP inhibitor. Source: ClinicalTrials (https://clinicaltrials.gov/, accessed on 22 February 2022).
