# Supplementary material for: Revisiting Proteasome Inhibitors: Molecular Underpinnings of Their Development, Mechanisms of Resistance and Strategies to Overcome Anti-Cancer Drug Resistance
Source: Molecules. 2022 Mar 28;27(7):2201. doi: 10.3390/molecules27072201 (PMC9000344; doi:10.3390/molecules27072201)
Supplement: Supplementary file 1 [file molecules-27-02201-s001.zip › molecules-1638513-supplementary.pdf]

Proteasome inhibitors in anticancer therapy: molecular underpinnings underlying their development and strategies to overcome resistance mechanisms

Carlota Leonardo-Sousa[1], Andreia Neves Carvalho[1], Romina A. Guedes[1,2], Pedro Fernandes[1,2], Natália Aniceto[1], Jorge A. R. Salvador[2,*], Maria João Gama[1,*] and Rita C. Guedes[1,*]

[1]Research Institute for Medicines (iMed.ULisboa), Faculty of Pharmacy, Universidade de Lisboa, 1649-003 Lisboa, Portugal.

[2]Laboratory of Pharmaceutical Chemistry, Faculty of Pharmacy, University of Coimbra, Coimbra, Portugal and Center for Neuroscience and Cell Biology (CNC), Center for Innovative Biomedicine and Biotechnology (CIBB), University of Coimbra, Coimbra, Portugal

Supplementary table S1- Clinical trials of combination therapies with 20S CP inhibitors, involving patients with relapsed and/or refractory disease, and who were previously treated with a 20S CP inhibitor. Source: ClinicalTrials (https://clinicaltrials.gov/).

Study (code)	Title	20S CP inhibitor	Other drugs	Purpose	Observations	Ref.
NCT00082784	Phase I Trial of Bortezomib (PS-341; NSC 681239) and Alvocidib (Flavopiridol NSC 649890) in Patients with Recurrent or Refractory Indolent B-Cell Neoplasms	Bortezomib	Alvocidib (cyclin-dependent kinases inhibitor)	To determine the recommended phase II dose of bortezomib and flavopiridol in patients with recurrent or refractory indolent B-cell neoplasms, and to determine the toxicity, pharmacodynamics and pharmacokinetics of this regimen.	Study completed.<br>One participant with multiple myeloma (MM), who progressed on prior bortezomib, achieved a complete response for the treatment in study.	[1]
NCT00087867<br>NCT00095680	An Open-label Study of the Efficacy, Safety, and Tolerability of Oral SCIO-469 in Treatment of Relapsed, Refractory Patients with Multiple Myeloma; A 24-week, Open-label Extension Study of the Efficacy, Safety, and Tolerability of Oral SCIO-469 in Treatment of Relapsed Refractory Patients with Multiple Myeloma	Bortezomib	SCIO-469 (inhibits p38a mitogen-activated protein kinase blocking synthesis of Il-1β, PGE2, VEGF, MIP-1αand TNFα)	To assess the efficacy, safety and tolerability of SCIO-469 as monotherapy, or in combination with bortezomib in relapsed, refractory patients with MM.	A phase II and completed study.<br>In addition to SCIO-469 treatment patients with disease progression received bortezomib.<br>In combination of SCIO-469 and bortezomib, the objective response rate was 32%, including 4 patients who had failed prior bortezomib.	[2]

Supplementary table S1- Clinical trials of combination therapies with 20S CP inhibitors, involving patients with relapsed and/or refractory disease, and who were previously treated with a 20S CP inhibitor. Source: ClinicalTrials (https://clinicaltrials.gov/).

Study (code)	Title	20S CP inhibitor	Other drugs	Purpose	Observations	Ref.
NCT00111813	Phase I Clinical Trial of Vorinostat (MK-0683) in Combination with Bortezomib in Patients with Advanced Multiple Myeloma	Bortezomib	Vorinostat (and dexamethasone at the time of disease progression, and after at least 1 cycle)	To determine the maximum tolerated dose for the combination of vorinostat and bortezomib in participants with refractory and/or relapsed and advanced MM and to assess the safety and tolerability of this regimen.	Study completed. Of 34 participants, 27% achieved partial response, but none achieved complete response; minimal response was noted in 6% and 59% continued with stable disease. Response rates were similar in participants previously exposed to bortezomib and participants who were naïve to bortezomib therapy. Among 18 participants who had previously received bortezomib, 22% achieved partial response. Of 7 patients who were considered to have disease refractory to prior bortezomib, 14% achieved partial response and the remaining 86% achieved stable disease. The median duration of response was 120 days among the 31 participants with stable disease or better, 125 days among the 16 participants with stable disease or better who had previously received bortezomib, 111 days for participants resistant to previous bortezomib, and 158 days for participants who achieved response after the addition of dexamethasone.	[3]

Supplementary table S1- Clinical trials of combination therapies with 20S CP inhibitors, involving patients with relapsed and/or refractory disease, and who were previously treated with a 20S CP inhibitor. Source: ClinicalTrials (https://clinicaltrials.gov/).

Study (code)	Title	20S CP inhibitor	Other drugs	Purpose	Observations	Ref.
NCT00153933	An Open-Label Phase I Study of the Safety and Efficacy of Bortezomib in Combination with CC-5013 in the Treatment of Subjects with Relapsed and Relapsed/Refractory Multiple Myeloma	Bortezomib	Lenalidomide (and dexamethasone if the disease is getting worse after 2 cycles of treatment)	To evaluate the safety of bortezomib combined with lenalidomide, in participants with relapsed and relapsed/refractory MM. To determine the maximum tolerated dose and the recommended phase II dose, and to determine the pharmacokinetics of this combination.	Study completed. Among the 24 participants who were refractory to prior bortezomib, lenalidomide, and/or thalidomide, 25% achieved at least partial response, and 50% achieved at least minimal response. Among the 2 participants refractory to bortezomib, 1 achieved a minimal response, and the other had progressive disease. Among 9 participants refractory to bortezomib and thalidomide, 1 achieved partial response, 3 achieved minimal response, 4 had stable disease, and 1 was unevaluable for response. Among the 3 patients refractory to all 3 agents, 1 achieved near-complete response, and 2 had stable disease.	[4]

Supplementary table S1- Clinical trials of combination therapies with 20S CP inhibitors, involving patients with relapsed and/or refractory disease, and who were previously treated with a 20S CP inhibitor. Source: ClinicalTrials (https://clinicaltrials.gov/).

Study (code)	Title	20S CP inhibitor	Other drugs	Purpose	Observations	Ref.
NCT00295932	A Phase II Study of the Novel Proteasome Inhibitor Bortezomib in Combination with Rituximab, Cyclophosphamide and Prednisone in Patients with Relapsed/Refractory Indolent B-Cell Lymphoproliferative Disorders and Mantle Cell Lymphoma (MCL)	Bortezomib	Cyclophosphamide, prednisone and rituximab (monoclonal antibody against CD20)	To evaluate the toxicity and to determine the maximum tolerated dose of bortezomib in combination with rituximab, cyclophosphamide, and prednisone in patients with relapsed or refractory indolent B-cell non-Hodgkin's lymphoma.	Study completed. In phase I, 4 participants in the weekly treatment group had received prior bortezomib. One patient with MCL had an initial partial response to bortezomib, but stable disease with a second course upon relapse, and with the combination therapy in study achieved a complete response. The other 3 patients did not respond to single agent bortezomib (2 stable disease, 1 progressive disease). with the combination therapy in study, 1 of these achieved a partial response, and 2 had stable disease. Three patients in the twice-weekly treatment group were bortezomib refractory. with the combination therapy in study, 2 of these patients achieved partial response and 1 had stable disease.	[5]

Supplementary table S1- Clinical trials of combination therapies with 20S CP inhibitors, involving patients with relapsed and/or refractory disease, and who were previously treated with a 20S CP inhibitor. Source: ClinicalTrials (https://clinicaltrials.gov/).

Study (code)	Title	20S CP inhibitor	Other drugs	Purpose	Observations	Ref.
NCT00310024	A Phase I Study of SAHA in Combination with Bortezomib in Relapsed and Refractory Multiple Myeloma	Bortezomib	Vorinostat and dexamethasone (after 2 cycles, in patients with less than a partial remission)	To determine the toxicity and to evaluate the maximum tolerated dose of vorinostat when given together with bortezomib in patients with relapsed or refractory MM. To determine whether giving vorinostat together with bortezomib inhibits histone deacetylation in normal cells (buccal mucosal cells and/or peripheral blood monocytes) as well as in MM cells. To evaluate the effect of dexamethasone when given together with vorinostat and bortezomib. To explore molecular mechanisms involved in apoptosis and to correlate the change of histone acetylation with the clinical outcome in participants.	No results were published. However, the principal investigator published an article related with a study with similar purposes. This study involved 19 participants who received bortezomib, 9 of whom were bortezomib-refractory, while the rest had progressive disease after an initial response lasting a median of 6 months. Eight of the 9 bortezomib-refractory participants were evaluated, 3 achieved partial response, 4 achieved stable disease and 1 achieved progressive disease. Of the 10 participants who had progressive disease and were subject to previous bortezomib-treatment, the response to the study regimen was evaluated on 9 participants, 1 achieved very good partial response, 2 achieved partial response, 5 achieved stable disease and 1 achieved progressive disease. The maximum tolerated dose of vorinostat in their study was 400 mg daily for 8 days every 21 days, with bortezomib administered at a dose of 1.3 mg/m^2 on days 1, 4, 8, and 11.	[6]

Supplementary table S1- Clinical trials of combination therapies with 20S CP inhibitors, involving patients with relapsed and/or refractory disease, and who were previously treated with a 20S CP inhibitor. Source: ClinicalTrials (https://clinicaltrials.gov/).

Study (code)	Title	20S CP inhibitor	Other drugs	Purpose	Observations	Ref.
NCT00310024 (continuation)	A Phase I Study of SAHA in Combination with Bortezomib in Relapsed and Refractory Multiple Myeloma	Bortezomib	Vorinostat and dexamethasone (after 2 cycles, in patients with less than a partial remission)	To determine the toxicity and to evaluate the maximum tolerated dose of vorinostat when given together with bortezomib in patients with relapsed or refractory MM. To determine whether giving vorinostat together with bortezomib inhibits histone deacetylation in normal cells (buccal mucosal cells and/or peripheral blood monocytes) as well as in MM cells. To evaluate the effect of dexamethasone when given together with vorinostat and bortezomib. To explore molecular mechanisms involved in apoptosis and to correlate the change of histone acetylation with the clinical outcome in participants.	Of the 6 bortezomib-refractory evaluable participants treated with the maximum tolerated dose, 2 achieved partial response, 4 achieved progressive disease; and of the 3 participants who had progressive disease and were subject to previous bortezomib-treatment, 2 achieved partial response and 1 achieved progressive disease. There was no improvement in response with the addition of dexamethasone. Pharmacodynamic changes in protein levels of NF-κB, IκB, p21CIP1 and acetylated tubulin in plasma cells isolated from the bone marrow on days 1 (pre-treatment) and 11 of vorinostat and bortezomib in the study did not correlate with the participants' clinical responses.	[6]

Supplementary table S1- Clinical trials of combination therapies with 20S CP inhibitors, involving patients with relapsed and/or refractory disease, and who were previously treated with a 20S CP inhibitor. Source: ClinicalTrials (https://clinicaltrials.gov/).

Study (code)	Title	20S CP inhibitor	Other drugs	Purpose	Observations	Ref.
NCT00316940	A Phase I Study of Samarium Sm-153 Lexidronam Combined with Bortezomib for Patients with Relapsed or Refractory Multiple Myeloma	Bortezomib	^{153}Sm-lexidronam pentasodium (radiopharmaceutical agent which is used as palliative therapy for metastatic bone disease, but emerging evidence suggests that it also has anti-myeloma activity)	To assess the safety, tolerability, toxicity and efficacy and determine the maximum tolerated dose of ^{153}Sm-lexidronam and bortezomib in patients with relapsed or refractory MM.	Study completed. Participants previously treated with bortezomib-containing regimens were allowed, and all of those participants had progressed disease following those regimens. The overall response rate for participants previously treated with a bortezomib-containing regimen was 15% compared with 27% in participants whose prior treatments did not include bortezomib. Two patients who responded to this less frequently dosed bortezomib with ^{153}Sm-lexidronam received the study treatment within 1 month of progressing from a combination of oral melphalan and bortezomib on a more frequent dosing schedule (4 doses every 4 weeks). These results suggest that the ^{153}Sm-lexidronam and bortezomib combination can achieve responses even among patients resistant to bortezomib/chemotherapy combination therapies.	[7]

Supplementary table S1- Clinical trials of combination therapies with 20S CP inhibitors, involving patients with relapsed and/or refractory disease, and who were previously treated with a 20S CP inhibitor. Source: ClinicalTrials (https://clinicaltrials.gov/).

Study (code)	Title	20S CP inhibitor	Other drugs	Purpose	Observations	Ref.
NCT00352742	A Phase I/II Study of ATN-224 and Bortezomib in Patients with Multiple Myeloma Relapsed from or Refractory to Bortezomib	Bortezomib	ATN-224 (antiangiogenic agent, an inhibitor of the enzyme superoxide dismutase 1)	The purposes of phase I were to determine a safe dose of ATN-224 and bortezomib to be used in the phase II portion of the study and to evaluate the preliminary evidence of efficacy, in patients with MM relapsed from or refractory to bortezomib. The purpose of phase II was to evaluate the efficacy of this combination.	Study terminated. A preclinical study with the combination of ATN-224 and bortezomib shows that the combination is more effective than either single agent in a bortezomib resistant cell line. In phase I portion 21 patients were enrolled. One experienced a complete response that was durable for over 10 months and 20 experienced disease progression within 90 days.	[8,9]
NCT00378209	An Open-Label Phase II Study of the Safety and Efficacy of Bortezomib, Lenalidomide, and Dexamethasone Combination Therapy for Patients with Relapsed or Relapsed and Refractory Multiple Myeloma	Bortezomib	Dexamethasone and lenalidomide	To evaluate the effectiveness and side effects of the bortezomib, lenalidomide and dexamethasone combination in relapsed or relapsed and refractory MM.	Study completed. The response to the lenalidomide-bortezomib--dexamethasone triplet regimen used in the present study was not significantly affected by prior bortezomib exposure.	[10]

Supplementary table S1- Clinical trials of combination therapies with 20S CP inhibitors, involving patients with relapsed and/or refractory disease, and who were previously treated with a 20S CP inhibitor. Source: ClinicalTrials (https://clinicaltrials.gov/).

Study (code)	Title	20S CP inhibitor	Other drugs	Purpose	Observations	Ref.
NCT00401011	An Open-Label Phase I/II Study of the Safety and Efficacy of Perifosine and Bortezomib with or without Dexamethasone for Patients with Relapsed or Refractory Multiple Myeloma Previously Treated with Bortezomib	Bortezomib	Perifosine (signal transduction modulator with multiple pathway effects, including inhibition of Akt, activation of c-Jun NH_2-terminal kinase, and upregulation of the death receptors DR4/DR5 expression, which subsequently induces cytotoxicity in MM cells) and dexamethasone (if the patient shows progressive disease)	The purpose of phase I portion was to determine the maximum tolerated dose of perifosine in combination with bortezomib in patients with relapsed or refractory MM previously treated with bortezomib. The purpose of phase II was to evaluate the efficacy, safety and tolerability of this combination with or without dexamethasone.	Study completed. Of the 53 response-evaluable participants with disease refractory to prior bortezomib, the overall response rate was 32%, including a 2% complete response rate, 11% partial response rate, and 19% minimal response rate. 43% of the participants had stable disease. 60% of the 53 participants received dexamethasone and the overall response rate was 34%, including 13% partial response rate, and 22% minimal response rate.<br>Of the 20 response-evaluable participants with bortezomib-relapsed disease, the overall response rate was 65%, including a 10% complete response rate and 35% partial response rate, and 20% minimal response rate. 35% of the participants had stable disease.	[11]

Supplementary table S1- Clinical trials of combination therapies with 20S CP inhibitors, involving patients with relapsed and/or refractory disease, and who were previously treated with a 20S CP inhibitor. Source: ClinicalTrials (https://clinicaltrials.gov/).

Study (code)	Title	20S CP inhibitor	Other drugs	Purpose	Observations	Ref.
NCT00407303	A Phase I/II Study of Obatoclax Mesylate (GX15-070MS) Administered in Combination with Bortezomib to Patients with Relapsed or Refractory Mantle Cell Lymphoma (MCL)	Bortezomib	Obatoclax Mesylate (restore apoptosis through inhibition of the Bcl-2 family of proteins)	To determine the maximum tolerated dose of obatoclax in combination with bortezomib and to evaluate the efficacy and safety of this combination regimen in patients with relapsed or refractory MCL.	Study completed. Of the 13 patients included in the efficacy-evaluable population, 1 participant who achieved complete response had received prior bortezomib. Of the 23 participants included in the safety/intent-to-treat population 7 participants were treated with prior bortezomib. Of this subset of participants, 3 achieved stable disease, 2 experienced disease progression as the best response and 2 were unevaluable.	[12]
NCT00426855	Weekly Bendamustine and Bortezomib Combination Therapy in Relapsed or Refractory Indolent Non-Hodgkin's Lymphoma or B-CLL: A Single-Center Phase 2 Study	Bortezomib	Bendamustine	To determine whether the combination of bendamustine and bortezomib in patients with indolent non-Hodgkin's lymphoma or chronic lymphocytic leukemia is safe and tolerable.	Study completed. One of the participants, with 1 MCL achieved a complete response, and was treated with prior bortezomib. The other participant with the same disease and also treated with prior bortezomib, achieved a partial response.	[13]
NCT00431340	A Phase II Study of Belinostat in Combination with Bortezomib in Patients with Relapsed, Refractory Multiple Myeloma	Bortezomib	Belinostat	To assess anti-tumor activity and safety of belinostat in combination with bortezomib in MM patient's refractory to or relapsed from at least one prior bortezomib--containing regimen.	This study has been terminated due to dose limiting toxicity.	

Supplementary table S1- Clinical trials of combination therapies with 20S CP inhibitors, involving patients with relapsed and/or refractory disease, and who were previously treated with a 20S CP inhibitor. Source: ClinicalTrials (https://clinicaltrials.gov/).

Study (code)	Title	20S CP inhibitor	Other drugs	Purpose	Observations	Ref.
NCT00431990	A Phase I/II Trial of Romidepsin (Depsipeptide) and Bortezomib in Patients with Relapsed Myeloma	Bortezomib	Dexamethasone and romidepsin	The purpose of phase I was to determine whether, and at what dose, romidepsin, bortezomib and dexamethasone can be safely administered to patients with MM. The purpose of phase II was to establish whether these 3 drugs combined are effective in the treatment of patients with MM. Lastly, it sought to examine the role of maintenance therapy with romidepsin.	According to ClinicalTrials.gov, the recruitment status of this study is unknown (the completion date has passed and the status has not been verified in more than 2 years). However, Harrison *et al.* published an article which revealed results of this study. Of the 6 patients who previously relapsed on bortezomib, 4 established disease stability or improved to partial response (1 partial response, 1 minor response and 2 no change response), with a median time to progression of 5.6 months, despite having rapidly progressive disease at the time of study entry.	[14]
NCT00483262	Phase I/II Trial of Combination CCI-779 (Temsirolimus) and Bortezomib (Velcade) in Relapsed and/or Relapsed/Refractory Multiple Myeloma	Bortezomib	Temsirolimus (an mTOR inhibitor)	To determine the safety of temsirolimus and bortezomib, and the highest dose of this drug that can be given to people safely. To evaluate the toxicity and efficacy of this combination, in patients with relapsed or refractory MM.	Study completed. In phase II of the study, 32 participants were previously treated with bortezomib. For the combinate therapy in study these participants had a minimal-or-better response rate of 38% and the partial-or-better response rate was 25%.	[15]

Supplementary table S1- Clinical trials of combination therapies with 20S CP inhibitors, involving patients with relapsed and/or refractory disease, and who were previously treated with a 20S CP inhibitor. Source: ClinicalTrials (https://clinicaltrials.gov/).

Study (code)	Title	20S CP inhibitor	Other drugs	Purpose	Observations	Ref.
NCT00483262 (continuation)	Phase I/II Trial of Combination CCI-779 (Temsirolimus) and Bortezomib (Velcade) in Relapsed and/or Relapsed/Refractory Multiple Myeloma	Bortezomib	Temsirolimus (an mTOR inhibitor)	To determine the safety of temsirolimus and bortezomib, and the highest dose of this drug that can be given to people safely. To evaluate the toxicity and efficacy of this combination, in patients with relapsed or refractory MM.	A minimal-or-better response rate of over 60% in participants not refractory to previous treatment with bortezomib, suggests an additive clinical benefit of temsirolimus in combination with bortezomib in these participants. This study suggests that mTOR inhibitors could be synergistic with proteasome inhibitors in MM, but they might not completely overcome resistance or resensitive cells which are resistant to bortezomib.	[15]

Supplementary table S1- Clinical trials of combination therapies with 20S CP inhibitors, involving patients with relapsed and/or refractory disease, and who were previously treated with a 20S CP inhibitor. Source: ClinicalTrials (https://clinicaltrials.gov/).

Study (code)	Title	20S CP inhibitor	Other drugs	Purpose	Observations	Ref.
NCT00514371	Phase 2/3 Randomized, Open-Label Clinical Trial of Tanespimycin (KOS-953) Plus Bortezomib Comparing Three Doses of Tanespimycin in Patients with Relapsed-Refractory Multiple Myeloma	Bortezomib	Tanespimycin (an HSP90 inhibitor which binds to the ATP-binding site of HSP90, blocking ATPase activity and resulting in the degradation of HSP90 client proteins via the ubiquitin--proteasome pathway)	To assess the dose-response relationship of objective response rate using EBMT/IBMTR criteria of any three dose levels of tanespimycin in combination with bortezomib after four treatment cycles in patients with relapsed and refractory MM.	Study completed. This phase II study, with the same combination was published by Richardson et al., in 2010. All 22 participants were previously treated with bortezomib and lenalidomide, and 18% were bortezomib-refractory. In the group treated with 175 mg/m^2 tanespimycin and 1.3 mg/m^2 bortezomib, the best responses observed of the combination therapy in study were 2 partial responses, 1 confirmed and 1 unconfirmed. The confirmed partial response was achieved by a participant who previously did not respond to 3-4 bortezomib treatments (the participant had been previously treated with 10 regimens, including 3 bortezomib-containing regimens and was refractory to the last regimen of bortezomib with vorinostat, with progressive disease observed after 3 cycles of treatment). The patient who achieved an unconfirmed partial response responded to more than 4 treatments with bortezomib. For these participants, disease progression-free survival was more than 2.9 months and 0.9 months, respectively.	[16]

Supplementary table S1- Clinical trials of combination therapies with 20S CP inhibitors, involving patients with relapsed and/or refractory disease, and who were previously treated with a 20S CP inhibitor. Source: ClinicalTrials (https://clinicaltrials.gov/).

Study (code)	Title	20S CP inhibitor	Other drugs	Purpose	Observations	Ref.
NCT00514371 (continuation)	Phase 2/3 Randomized, Open-Label Clinical Trial of Tanespimycin (KOS-953) Plus Bortezomib Comparing Three Doses of Tanespimycin in Patients with Relapsed-Refractory Multiple Myeloma	Bortezomib	Tanespimycin (an HSP90 inhibitor which binds to the ATP-binding site of HSP90, blocking ATPase activity and resulting in the degradation of HSP90 client proteins via the ubiquitin--proteasome pathway)	To assess the dose-response relationship of objective response rate using EBMT/IBMTR criteria of any three dose levels of tanespimycin in combination with bortezomib after four treatment cycles in patients with relapsed and refractory MM.	In the group treated with 340 mg/m^2 tanespimycin and 1.3 mg/m^2 bortezomib, the best response observed of the combination therapy in study was minimal response of a participant who responded to 3 or 4 prior treatments with bortezomib. For this participants, disease progression-free survival was more than 12.7 months.<br>The study was closed early in its course for logistic and resource reasons unrelated to safety and efficacy.	[16]

Supplementary table S1- Clinical trials of combination therapies with 20S CP inhibitors, involving patients with relapsed and/or refractory disease, and who were previously treated with a 20S CP inhibitor. Source: ClinicalTrials (https://clinicaltrials.gov/).

Study (code)	Title	20S CP inhibitor	Other drugs	Purpose	Observations	Ref.
NCT00532389	A Phase Ib, Multi-center, Open-label, Dose-escalation Study of Oral LBH589 When Administered in Combination with Bortezomib in Adult Patients with Multiple Myeloma	Bortezomib	Panobinostat and dexamethasone (could be administered from cycle 2 onward)	To determine the maximum tolerated dose of panobinostat in combination with bortezomib. To assess safety, tolerability, pharmacokinetic and pharmacodynamic profile of the combined treatments in MM. In dose expansion phase to explore in a non-continuous panobinostat schedule with bortezomib and dexamethasone, safety and tolerability and pharmacokinetic profile of panobinostat and bortezomib with and without dexamethasone.	Study completed. Clinical benefit was observed in bortezomib-refractory and bortezomib- and immunomodulator-refractory participants. Among the 19 bortezomib-refractory patients (14 refractory to both bortezomib and dexamethasone), 26.3% achieved partial response or better, and 42.1% of participants had minimal response or better. Among the 13 patients refractory to both bortezomib and immunomodulators, 15.4% of participants achieved partial response or better, and 38.5% of participants had minimal response or better.	[17]

Supplementary table S1- Clinical trials of combination therapies with 20S CP inhibitors, involving patients with relapsed and/or refractory disease, and who were previously treated with a 20S CP inhibitor. Source: ClinicalTrials (https://clinicaltrials.gov/).

Study (code)	Title	20S CP inhibitor	Other drugs	Purpose	Observations	Ref.
NCT00603447	Phase 1b Multicenter Dose Escalation Study of Carfilzomib with Lenalidomide and Dexamethasone for Safety and Activity in Relapsed Multiple Myeloma	Carfilzomib	Dexamethasone and lenalidomide	To evaluate the safety, efficacy and pharmacodynamics and to determine maximum tolerated dose of carfilzomib in combination with lenalidomide and dexamethasone in patients with relapsed MM.	Study completed. In the phase 2 dose expansion at the maximum planned dose, for the 13 participants treated with the maximum planned dose and bortezomib-refractory, the overall response rate was 69.2%, with a median duration of response of 22.1 months and median progression-free survival of 15.4 months. In this study, this combinatory therapy demonstrated robust, rapid and durable clinical activity with an acceptable tolerability profile, including patients who were refractory to bortezomib and lenalidomide.	[18,19]
NCT00706953	A Phase II Single Arm Study of VELCADE and DOXIL (PLD) in Patients with Relapsed Multiple Myeloma Previously Treated with VELCADE	Bortezomib	Pegylated liposomal doxorubicin	To evaluate the overall response rate and safety of the combination of bortezomib and pegylated liposomal doxorubicin in patients with relapsed MM who had been previously treated with bortezomib.	This study has been withdrawn prior to enrollment.	

Supplementary table S1- Clinical trials of combination therapies with 20S CP inhibitors, involving patients with relapsed and/or refractory disease, and who were previously treated with a 20S CP inhibitor. Source: ClinicalTrials (https://clinicaltrials.gov/).

Study (code)	Title	20S CP inhibitor	Other drugs	Purpose	Observations	Ref.
NCT00765102	A Phase II Trial of Romidepsin and Bortezomib for Multiple Myeloma Patients with Relapsed or Refractory Disease	Bortezomib	Romidepsin	To evaluate the efficacy and safety of romidepsin in combination with bortezomib for patients with refractory or relapsed MM. Patients were included into one of two strata, bortezomib-resistant or bortezomib non-resistant.	This study has been terminated, due to a change regarding the Sponsor's research strategy, but safety concerns were not a factor. Due to the early termination of the study, the efficacy data of this combined treatment was not analyzed.	

Supplementary table S1- Clinical trials of combination therapies with 20S CP inhibitors, involving patients with relapsed and/or refractory disease, and who were previously treated with a 20S CP inhibitor. Source: ClinicalTrials (https://clinicaltrials.gov/).

Study (code)	Title	20S CP inhibitor	Other drugs	Purpose	Observations	Ref.
NCT00773838	An International, Multicenter, Open-Label Study of Vorinostat (MK0683) in Combination with Bortezomib in Patients with Relapsed or Refractory Multiple Myeloma	Bortezomib	Vorinostat and dexamethasone (in patients with progressive disease after 2 cycles or no change after 4 cycles)	To determine efficacy and tolerability of vorinostat in combination with standard doses of bortezomib (1,3 mg/m^2) in MM patients considered refractory to at least one prior bortezomib-containing regimen and to have received at least one dose of an immunomodulator (thalidomide or lenalidomide)-based regimen.	A phase II completed study. Of the 142 treated patients, none achieved a complete response under the study therapy; 11.3% had a partial response (with a median time to response of 44 days and a median duration of response of 211 days); 7.7% had minimal response (with a median time to response of 85 days) and 61.3% achieved stable disease (with a median duration of 66 days). Median overall survival was 11.2 months, with a 2-year survival rate of 32%. The median progression-free survival was 3.13 months and the median time to progression was 3.47 months. This study concluded that the combination of vorinostat with bortezomib is active in MM patient's refractory to novel treatment modalities and offers a new therapeutic option for this difficult-to-treat patient population.	[20]

Supplementary table S1- Clinical trials of combination therapies with 20S CP inhibitors, involving patients with relapsed and/or refractory disease, and who were previously treated with a 20S CP inhibitor. Source: ClinicalTrials (https://clinicaltrials.gov/).

Study (code)	Title	20S CP inhibitor	Other drugs	Purpose	Observations	Ref.
NCT00793650	Combination High Dose Melphalan and Autologous PBSC Transplant with Bortezomib for Multiple Myeloma: A Dose and Schedule Finding Study	Bortezomib	Melphalan and autologous peripheral blood stem cell transplantation	To evaluate the safety of melphalan and autologous peripheral blood stem cell transplantation in combination with bortezomib, in patients with MM and a response of less than a very good partial remission following at least one induction regimen.	A phase I/II terminated study. A poor response to induction therapy occurred despite 62% of patients having received bortezomib-based induction. Patients were randomized to receive bortezomib 24 hours before the first dose of melphalan (arm A) or received bortezomib 24 following the second dose of melphalan (arm B). In arm A and arm B, respectively, 11 and 12 patients were previously treated with bortezomib. Among the fraction of patients that had received prior bortezomib, in arm A, 55% of the patients achieved very good partial remission or better (2 complete response); in arm B, 58% of the patients achieved very good partial remission or better (5 complete response). The overall response rate and the very good partial remission or better rate were independent of prior bortezomib exposure.	[21]

Supplementary table S1- Clinical trials of combination therapies with 20S CP inhibitors, involving patients with relapsed and/or refractory disease, and who were previously treated with a 20S CP inhibitor. Source: ClinicalTrials (https://clinicaltrials.gov/).

Study (code)	Title	20S CP inhibitor	Other drugs	Purpose	Observations	Ref.
NCT00858234	A Multicenter, Open-Label, Phase I Study of MK0683 in Combination with Bortezomib in Patients with Relapsed and/or Refractory Multiple Myeloma	Bortezomib	Vorinostat	To determine recommended clinical dose, and to evaluate the safety, tolerability, pharmacokinetics and efficacy of vorinostat in combination with bortezomib in Japanese patients with relapsed and/or refractory MM.	Study completed. Of the 9 patients, 2 were refractory to bortezomib and both experienced grade 4 thrombocytopenia after study initiation. These results raised concerns about tolerability in bortezomib-refractory patients, prompting a protocol amendment to include only patients who were not considered refractory to bortezomib.	[22]

Supplementary table S1- Clinical trials of combination therapies with 20S CP inhibitors, involving patients with relapsed and/or refractory disease, and who were previously treated with a 20S CP inhibitor. Source: ClinicalTrials (https://clinicaltrials.gov/).

Study (code)	Title	20S CP inhibitor	Other drugs	Purpose	Observations	Ref.
NCT01002248	A Phase III Randomized Study to Assess the Efficacy and Safety of Perifosine Added to the Combination of Bortezomib and Dexamethasone in Multiple Myeloma Patients	Bortezomib	Dexamethasone, perifosine and perifosine placebo	To evaluate the efficacy and safety of perifosine when added to the combination of bortezomib and dexamethasone in MM patients who have relapsed on a prior bortezomib treatment regimen and received between 1 and 4 prior lines of therapy, but they must not be refractory to any bortezomib-containing regimen.	Terminated study. The participants were randomized 1:1 to perifosine, bortezomib and dexamethasone treatment or placebo, bortezomib and dexamethasone treatment. For the first group the progression-free survival was of 22.7 weeks, and of 39.0 weeks for the second group. The overall response rates were 20.3% and 27.3%, respectively, and the clinical benefit rates were 46.4% and 43.9%. For the placebo group the median overall survival was 83.3 weeks and for perifosine group was 141.9 weeks (but did not achieve significance). This study showed no benefit in progression-free survival or overall response rate when adding perifosine to bortezomib and dexamethasone in participants with highly resistant, relapsed and refractory MM previously treated with bortezomib.	[23]

Supplementary table S1- Clinical trials of combination therapies with 20S CP inhibitors, involving patients with relapsed and/or refractory disease, and who were previously treated with a 20S CP inhibitor. Source: ClinicalTrials (https://clinicaltrials.gov/).

Study (code)	Title	20S CP inhibitor	Other drugs	Purpose	Observations	Ref.
NCT01083602	A Phase II, Multi-center, Single Arm, Open Label Study of Panobinostat in Combination with Bortezomib and Dexamethasone in Patients with Relapsed and Bortezomib-refractory Multiple Myeloma	Bortezomib	Dexamethasone and panobinostat	To assess the effectiveness of the combination of panobinostat plus bortezomib and dexamethasone in patients with relapsed and refractory MM, who are bortezomib-refractory and have received at least 2 prior lines of therapy (patients must have been exposed to an immunomodulator and progressed on or within 60 days of their last bortezomib-containing line of therapy).	Study completed. In 55 participants, the overall response rate was 34.5% (1 near-complete response and 18 partial responses). For these participants, the median of duration of response was 1.4 months and the median of progression-free survival was 6.0 months. 18.2% of the participants achieved a minimal response. In exploratory analysis, 5.5% of the participants achieved a very good partial response, 36.4% had stable disease, 5.5% presented progressive disease and response could not be assessed in the remaining 5.5% participants. The response rates to this combination therapy appeared to be higher in patients whose last bortezomib-containing regimen was not their last line of therapy before study entry. For all participants, median overall survival was not reached after a median follow-up of 8.3 months. Median progression-free survival was 5.4 months. In this study it was concluded that panobinostat, when combined with bortezomib and dexamethasone, can recapture responses in heavily pretreated, bortezomib-refractory MM patients.	[24]

Supplementary table S1- Clinical trials of combination therapies with 20S CP inhibitors, involving patients with relapsed and/or refractory disease, and who were previously treated with a 20S CP inhibitor. Source: ClinicalTrials (https://clinicaltrials.gov/).

Study (code)	Title	20S CP inhibitor	Other drugs	Purpose	Observations	Ref.
NCT01114282	A Phase I Study of Bortezomib (VELCADE) in Combination with Pralatrexate in Relapsed/Refractory Multiple Myeloma	Bortezomib	Pralatrexate (inhibits folic acid metabolism by competitively binding to and inhibiting the enzyme dihydrofolate reductase, which converts the dihydrofolate to tetrahydrofolate. The tetrahydrofolate is required for the synthesis and catabolism of several amino acids, methylation of ribonucleic acids, and synthesis of purines and thymidine. Depletion of thymidine results in the inhibition of DNA replication and thus interferes with cell proliferation)	To determine the maximum tolerated dose of pralatrexate in combination with bortezomib in patients with relapsed or refractory MM that have progressed following at least on prior therapy and must have received at least 1 prior line of systemic treatment that may have included bortezomib.	Study completed. Ten of 11 participants had previously received bortezomib, 6 were refractory to bortezomib and 1 was refractory to carfilzomib. Prior treatment history did not predict response to the combination therapy evaluated. Of the 11 participants, 1 had a very good partial response, 1 had a partial response, 1 had a minimal response, 6 had stable disease and 2 had progressive disease. The participant who achieved a very good partial response was refractory to bortezomib and had previously undergone autologous stem cell transplant. The patient who achieved a partial response had also undergone prior autologous stem cell transplantation, but had not previously received bortezomib.	[25]

Supplementary table S1- Clinical trials of combination therapies with 20S CP inhibitors, involving patients with relapsed and/or refractory disease, and who were previously treated with a 20S CP inhibitor. Source: ClinicalTrials (https://clinicaltrials.gov/).

Study (code)	Title	20S CP inhibitor	Other drugs	Purpose	Observations	Ref.
NCT01125293	Phase I/II Study of Combination Everolimus (RAD001), and Rituximab (Rituxan), OR Everolimus, Bortezomib (Velcade, PS-341), and Rituximab in Patients with Relapsed and/or Relapsed/Refractory Waldenstrom's Macroglobulinemia	Bortezomib	Everolimus (a mTOR inhibitor) and rituximab	The phase I portion was to determine the maximum tolerated dose of everolimus and rituximab combination or everolimus, bortezomib and rituximab combination. The phase II portion was to evaluate the depth of responses to the everolimus, rituximab and bortezomib combination, in patients with relapsed and/or relapsed/refractory Waldenstrom's macroglobulinemia.	This study is ongoing, but not recruiting participants. Participants who received prior treatment with rituximab, bortezomib and/or everolimus are permitted, but must not have been refractory to rituximab. Results were published in 2015, included 46 patients, 98% who received prior rituximab, 57% who received prior bortezomib and 54% who received prior bortezomib and rituximab combination. Among the 36 participants who received full dose therapy of all 3 agents, 89% experienced at least a minimal response. This response rate indicated that this combination is highly effective in achieving a response despite prior treatment history (97% of these participants received prior rituximab therapy and 56% received bortezomib-based therapy with 53% receiving prior bortezomib/rituximab combination). This study suggests that, potentially, the addition of everolimus can be useful in enhancing response or overcoming resistance to prior therapy with bortezomib or rituximab.	[26]

Supplementary table S1- Clinical trials of combination therapies with 20S CP inhibitors, involving patients with relapsed and/or refractory disease, and who were previously treated with a 20S CP inhibitor. Source: ClinicalTrials (https://clinicaltrials.gov/).

Study (code)	Title	20S CP inhibitor	Other drugs	Purpose	Observations	Ref.
NCT01160484	A Phase II Study of Pegylated Liposomal Doxorubicin, Bortezomib, Dexamethasone and Lenalidomide (DVD-R) for Patients with Relapsed/Refractory Multiple Myeloma	Bortezomib	Dexamethasone, lenalidomide and pegylated liposomal doxorubicin	To evaluate the efficacy and safety of lenalidomide in combination with bortezomib, pegylated liposomal doxorubicin and dexamethasone, in adult patients with relapsed/refractory MM.	Study completed. Response rates were high regardless of the type of prior treatment including those exposed to bortezomib, pegylated liposomal doxorubicin, glucocorticosteroids or lenalidomide. The clinical benefit (minor response or better) rate was 81.8% on participants who received prior treatment regimen(s) containing bortezomib.	[27]
NCT01163357	Phase I Study of Bortezomib with or without Total Marrow Irradiation (TMI) Using Intensity Modulated Radiation Therapy (IMRT) in Combination with Fludarabine (FLU) and Melphalan (MEL) as a Preparative Regimen for Allogeneic Hematopoietic Stem Cell (HSC) Transplantation in Patients with High Risk Multiple Myeloma	Bortezomib	Fludarabine phosphate (a purine analog), melphalan and total marrow irradiation	To evaluate the toxicity and best dose of bortezomib when given together with fludarabine phosphate and melphalan with or without total marrow irradiation in the treatment of patients undergoing donor peripheral blood stem cell transplant for high-risk stage I or II MM.	This study is ongoing, but not recruiting participants. It will include participants with relapsed MM following previous autologous stem cell transplant and with relapsed/refractory disease on new targeted therapies, *e.g.* thalidomide, lenalidomide, bortezomib, or other new agents such as carfilzomib, pomalidomide.	

Supplementary table S1- Clinical trials of combination therapies with 20S CP inhibitors, involving patients with relapsed and/or refractory disease, and who were previously treated with a 20S CP inhibitor. Source: ClinicalTrials (https://clinicaltrials.gov/).

Study (code)	Title	20S CP inhibitor	Other drugs	Purpose	Observations	Ref.
NCT01164709	Phase I Trial of Nelfinavir and Bortezomib in Advanced Hematologic Malignancies	Bortezomib	Nelfinavir mesylate (HIV protease inhibitors)	To assess the safety of nelfinavir mesylate in combination with bortezomib in patients with relapsed or progressive, advanced hematologic malignancies. To establish the phase II recommended dose of nelfinavir mesylate in these patients.	Study completed. In the dose escalation cohort of the trial, 7 bortezomib-resistant MM patients were included. Over all dose levels, of these 7 patients treated in the trial, 4 achieved a partial response, 2 a minor response, and 1 progressive disease. In the extension cohort of the trial 6 bortezomib-resistant MM patients were included. Three of these patients achieved a partial response, 2 achieved a minor response and 1 progressive disease, with nelfinavir and bortezomib treatment. Of 11 patients with relapsed, bortezomib-resistant MM treated, 4 achieved a partial response, 2 a minor response and 2 stable disease for 2 cycles or more with nelfinavir and bortezomib treatment, corresponding to a clinical benefit rate of 55%. This study indicated that nelfinavir can be used to resensitize patients with proteasome inhibitor-refractory MM for proteasome inhibitor treatment.	[28]

Supplementary table S1- Clinical trials of combination therapies with 20S CP inhibitors, involving patients with relapsed and/or refractory disease, and who were previously treated with a 20S CP inhibitor. Source: ClinicalTrials (https://clinicaltrials.gov/).

Study (code)	Title	20S CP inhibitor	Other drugs	Purpose	Observations	Ref.
NCT01168804	Bendamustine Plus Bortezomib Plus Dexamethasone in the Treatment of Stage II/III Relapsed or Refractory Multiple Myeloma	Bortezomib	Bendamustine and dexamethasone	To evaluate efficacy and safety of the combination regimen of bortezomib, bendamustine and dexamethasone in patients with relapsed or refractory MM.	A phase II completed study. Of 79 participants, 50 had been previously treated with bortezomib (1-3 lines). For the drug combination evaluated, 28% of these participants achieved a very good partial response or better, the overall response rate was 56%, the median progression-free survival was 8.6 months and the median overall survival was 25.6 months. While 48.3% of the 29 bortezomib-naïve participants achieved a very good partial response or better, the overall response rate was 69.0%, the median progression-free survival was 12.4 months and the median overall survival was not reached. Thirty-two participants were previously treated with lenalidomide and bortezomib. For the drug combination evaluated, 18.8% of these participants achieved a very good partial response or better, the overall response rate was 46.9%, the median progression-free survival was 7.1 months and the median overall survival was 17.4 months.	[29]

Supplementary table S1- Clinical trials of combination therapies with 20S CP inhibitors, involving patients with relapsed and/or refractory disease, and who were previously treated with a 20S CP inhibitor. Source: ClinicalTrials (https://clinicaltrials.gov/).

Study (code)	Title	20S CP inhibitor	Other drugs	Purpose	Observations	Ref.
NCT01168804 (continuation)	Bendamustine Plus Bortezomib Plus Dexamethasone in the Treatment of Stage II/III Relapsed or Refractory Multiple Myeloma	Bortezomib	Bendamustine and dexamethasone	To evaluate efficacy and safety of the combination regimen of bortezomib, bendamustine and dexamethasone in patients with relapsed or refractory MM.	Nineteen participants were previously treated with neither lenalidomide nor bortezomib. For the drug combination evaluated, 47.4% of these participants achieved a very good partial response or better, the overall response rate was 63.2% and the median progression-free survival was 12.0 months. The overall response rate was slightly but not considered significantly lower in patients previously exposed to bortezomib or to bortezomib and lenalidomide compared, respectively, with bortezomib-naïve patients and patients pretreated with neither of these 2 drugs. In bortezomib-pretreated patients, overall response rate were similar, independent of the number of pretreatments.	[29]

Supplementary table S1- Clinical trials of combination therapies with 20S CP inhibitors, involving patients with relapsed and/or refractory disease, and who were previously treated with a 20S CP inhibitor. Source: ClinicalTrials (https://clinicaltrials.gov/).

Study (code)	Title	20S CP inhibitor	Other drugs	Purpose	Observations	Ref.
NCT01183949	A Phase I/II Open-label Multicenter Study of AT7519M Alone and in Combination with Bortezomib in Patients with Previously Treated Multiple Myeloma	Bortezomib	AT7519M (cyclin kinase inhibitor)	To determine whether AT7519M alone or AT7519M plus bortezomib are effective treatments in patients with previously treated and relapsed and/or refractory MM.	Study completed. The participants included in this study had disease progression following at least 2 systemic treatments for MM and had to be refractory to the last bortezomib. Although no significant efficacy was seen following treatment with AT7519M alone in heavily pretreated participants, the combination of AT7519M with bortezomib achieved significant responses (33% of the participants achieved partial or better response) in a proportion of patients who were either pre-treated with or refractory to treatment with bortezomib.	[30]
NCT01204164	Phase 1 Dose-Escalation and Pharmacokinetic Study of TG02 Citrate in Patients with Advanced Hematological Malignancies	Carfilzomib	Dexamethasone and TG02 citrate (cyclin-dependent kinase 9)	To determine the highest dose of TG02 citrate that can be safely given to patients with different types of hematological malignancy. To evaluate the safety and tolerability of once-weekly dosing at the maximum-tolerated dose/recommended phase 2 dose of TG02 in combination with carfilzomib.	Study completed. To evaluate TG02 in combination with carfilzomib, participants who received prior therapies including bortezomib and an immunomodulatory agent were included. This drug combination was also evaluated in carfilzomib-refractory participants. Results of a study of this combination were reported in 2015 (not is possible confirm that is the same clinical trial). The results revealed that all participants who had a response (minimal response or better) were carfilzomib-refractory in a previous treatment regimen.	[31,32]

Supplementary table S1- Clinical trials of combination therapies with 20S CP inhibitors, involving patients with relapsed and/or refractory disease, and who were previously treated with a 20S CP inhibitor. Source: ClinicalTrials (https://clinicaltrials.gov/).

Study (code)	Title	20S CP inhibitor	Other drugs	Purpose	Observations	Ref.
NCT01248923	A Study of ARRY-520 and Bortezomib Plus Dexamethasone in Patients with Relapsed/Refractory Multiple Myeloma	Bortezomib	Dexamethasone and filanesib (inhibitor of kinesin spindle proteins, which are critical for normal mitosis. Their inhibition results in the formation of a monopolar spindle, causing mitotic arrest and apoptosis).<br>Due to the exacerbated neutropenia observed, filgrastim (a human granulocyte colony-stimulating factor) was administered.	To establish the maximum tolerated dose of filanesib in combination with bortezomib and dexamethasone in patients with recurrent/refractory MM and to obtain preliminary estimates of efficacy and possible biomarkers to predict response.	A phase I completed study. On the dose-escalation phase, among the 15 participants whose disease was previously sensitive to proteasome inhibitors (bortezomib and/or carflzomib), treated with 1.25 mg/m^2 or higher dose of filanesib, the overall response rate was of 40%, the disease control rate was 87% and the median duration of response was 17.2 months. In the same dosing subset, 14 participants were refractory to proteasome inhibitors. Among these participants, 2 achieved a very good partial response and 2 achieved a partial response (overall response rate of 29%), with a disease control rate of 64%.	[33]
NCT01281917	A Phase II Study of Velcade and Temsirolimus for Relapsed or Refractory B-cell Non-Hodgkin Lymphoma	Bortezomib	Temsirolimus	To evaluate the safety, tolerability and efficacy of bortezomib in combination with temsirolimus in relapsed or refractory B-cell non-Hodgkin lymphoma.	Study completed. Three patients had previously received treatment with bortezomib, 1 of whom was refractory to a prior bortezomib-containing regimen. That patient had a partial response to the drug combination evaluated. Of the other 2 patients, 1 had disease progression, and the other had stable disease.	[34]

Supplementary table S1- Clinical trials of combination therapies with 20S CP inhibitors, involving patients with relapsed and/or refractory disease, and who were previously treated with a 20S CP inhibitor. Source: ClinicalTrials (https://clinicaltrials.gov/).

Study (code)	Title	20S CP inhibitor	Other drugs	Purpose	Observations	Ref.
NCT01297764	A Phase I/II Study of Carfilzomib, Lenalidomide, Vorinostat, and Dexamethasone in Relapsed and/or Refractory Multiple Myeloma	Carfilzomib	Vorinostat, dexamethasone and lenalidomide	To evaluate the feasibility of combining these 4 drugs for the treatment of relapsed and/or refractory MM. To determine the maximum tolerated dose and to evaluate the safety/tolerability and efficacy of this regimen.	This study is ongoing, but not recruiting participants. Results were published in 2015, which included 17 participants. All participants were previously exposed to bortezomib and 6 were refractory to bortezomib, lenalidomide, dexamethasone. No dose-limiting toxicities were observed, and, subsequently, the maximum tolerated dose was not reached. Of all participants, 12% had a very good partial response, 41% achieved a partial response, 29% achieved stable disease, 12% had progressive disease and 6% were not evaluable for response. For participants who achieved partial or better response, the median of the time to response was 2 months, and the median of the duration of the response was 15 months. Considering all participants, the median of progression-free survival was 12 months.	[35]

Supplementary table S1- Clinical trials of combination therapies with 20S CP inhibitors, involving patients with relapsed and/or refractory disease, and who were previously treated with a 20S CP inhibitor. Source: ClinicalTrials (https://clinicaltrials.gov/).

Study (code)	Title	20S CP inhibitor	Other drugs	Purpose	Observations	Ref.
NCT01301807	Phase 1/1b Study of the Efficacy and Safety of the Combination of Panobinostat Plus Carfilzomib in Patients with Relapsed/Refractory Myeloma	Carfilzomib	Panobinostat (dexamethasone weekly could be added at the investigator's discretion during Phase Ib)	To evaluate the safety and efficacy of the combination of carfilzomib with panobinostat and to determine the maximum tolerated and recommended doses that can be given to patients with relapsed/refractory MM with failure to at least 2 lines of treatment which must include at least one immunomodulator (thalidomide, lenalidomide) and proteasome inhibitor (bortezomib).	According to ClinicalTrials.gov, the recruitment status of this study is unknown. Results were published in 2019. Between 2 August 2011 and 29 April 2016, a total of 47 participants were enrolled and treated. The median prior lines of therapy were 4 (range 2-16), 96% of the participants were previously exposed to bortezomib, 28% to carfilzomib, 95.7% to one of the 2 proteasome inhibitors, and 68.1% were bortezomib-refractory and 27.7% were carfilzomib-refractory. In 27 participants previously exposed to one proteasome inhibitor, treated at maximum recommended dose and whose were evaluable for response: the overall response rate was 40.7% (1 complete response, 3 very good partial responses and 7 partial responses), 14.8%, 11.1% and 33.3% of the participants achieved a minimal response, stable disease and progressive disease, respectively.	[36]

Supplementary table S1- Clinical trials of combination therapies with 20S CP inhibitors, involving patients with relapsed and/or refractory disease, and who were previously treated with a 20S CP inhibitor. Source: ClinicalTrials (https://clinicaltrials.gov/).

Study (code)	Title	20S CP inhibitor	Other drugs	Purpose	Observations	Ref.
NCT01301807 (continuation)	Phase 1/1b Study of the Efficacy and Safety of the Combination of Panobinostat Plus Carfilzomib in Patients with Relapsed/Refractory Myeloma	Carfilzomib	Panobinostat (dexamethasone weekly could be added at the investigator's discretion during Phase Ib)	To evaluate the safety and efficacy of the combination of carfilzomib with panobinostat and to determine the maximum tolerated and recommended doses that can be given to patients with relapsed/refractory MM with failure to at least 2 lines of treatment which must include at least one immunomodulator (thalidomide, lenalidomide) and proteasome inhibitor (bortezomib).	In 21 bortezomib-refractory participants, treated at maximum recommended dose and were evaluable for response: the overall response rate was 38.1% (1 complete response, 1 very good partial response and 6 partial responses), 14.3%, 9.5% and 38.1% of the participants achieved a minimal response, stable disease and progressive disease, respectively. In 11 carfilzomib-refractory participants, treated at maximum recommended dose and whose were evaluable for response: the overall response rate was 27.3% (1 complete response, 1 very good partial response and 1 partial responses), 9.1%, 27.3% and 36.4% of the participants achieved a minimal response, stable disease and progressive disease, respectively. In 17 participants previously exposed to bortezomib, treated at maximum recommended dose and with dexamethasone, the overall response rate was 52.9% (1 complete response, 2 very good partial responses and 6 partial responses), 11.8%, 11.8% and 23.5% of the participants achieved a minimal response, stable disease and progressive disease, respectively.	[36]

Supplementary table S1- Clinical trials of combination therapies with 20S CP inhibitors, involving patients with relapsed and/or refractory disease, and who were previously treated with a 20S CP inhibitor. Source: ClinicalTrials (https://clinicaltrials.gov/).

Study (code)	Title	20S CP inhibitor	Other drugs	Purpose	Observations	Ref.
NCT01301807 (continuation)	Phase 1/1b Study of the Efficacy and Safety of the Combination of Panobinostat Plus Carfilzomib in Patients with Relapsed/Refractory Myeloma	Carfilzomib	Panobinostat (dexamethasone weekly could be added at the investigator's discretion during Phase Ib)	To evaluate the safety and efficacy of the combination of carfilzomib with panobinostat and to determine the maximum tolerated and recommended doses that can be given to patients with relapsed/refractory MM with failure to at least 2 lines of treatment which must include at least one immunomodulator (thalidomide, lenalidomide) and proteasome inhibitor (bortezomib).	In 10 participants previously exposed to bortezomib, treated at maximum recommended dose and without dexamethasone, the overall response rate was 20.0% (1 very good partial response and 1 partial response), 20.0%, 10.0% and 50.0% of the participants achieved a minimal response, stable disease and progressive disease, respectively. In 13 bortezomib-refractory participants, treated at maximum recommended dose and with dexamethasone, the overall response rate was 53.8% (1 complete response, 1 very good partial response and 5 partial responses), 15.4%, 7.7% and 23.1% of the participants achieved a minimal response, stable disease and progressive disease, respectively. In 8 bortezomib-refractory participants, treated at maximum recommended dose and without dexamethasone, the overall response rate was 12.5% (1 partial response), 12.5%, 12.5% and 62.5% of the participants achieved a minimal response, stable disease and progressive disease, respectively.	[36]

Supplementary table S1- Clinical trials of combination therapies with 20S CP inhibitors, involving patients with relapsed and/or refractory disease, and who were previously treated with a 20S CP inhibitor. Source: ClinicalTrials (https://clinicaltrials.gov/).

Study (code)	Title	20S CP inhibitor	Other drugs	Purpose	Observations	Ref.
NCT01301807 (continuation)	Phase 1/1b Study of the Efficacy and Safety of the Combination of Panobinostat Plus Carfilzomib in Patients with Relapsed/Refractory Myeloma	Carfilzomib	Panobinostat (dexamethasone weekly could be added at the investigator's discretion during Phase Ib)	To evaluate the safety and efficacy of the combination of carfilzomib with panobinostat and to determine the maximum tolerated and recommended doses that can be given to patients with relapsed/refractory MM with failure to at least 2 lines of treatment which must include at least one immunomodulator (thalidomide, lenalidomide) and proteasome inhibitor (bortezomib).	The researchers concluded from study results that carfilzomib can be safely combined with panobinostat and this regimen could be used as a bridge therapy in patients with relapsed/refractory MM and a higher degree of proteasome inhibitor and immunomodulator refractoriness, however with a lower overall response rate, progression-free survival and overall survival: 39%, 3 months and 17.2 months, respectively, to all participants.	[36]
NCT01315873	Phase II Trial of Bortezomib and Bendamustine in the Treatment of Relapsed/Refractory Myeloma	Bortezomib	Bendamustine	To evaluate the toxicity and the efficacy of bortezomib and bendamustine combined in patients with relapsed/refractory MM and who should have progressed on bortezomib.	Study terminated before all data analysis was completed.	

Supplementary table S1- Clinical trials of combination therapies with 20S CP inhibitors, involving patients with relapsed and/or refractory disease, and who were previously treated with a 20S CP inhibitor. Source: ClinicalTrials (https://clinicaltrials.gov/).

Study (code)	Title	20S proteasome inhibitor	Other drugs	Purpose	Observations	Ref.
NCT01332617	Phase II Study of Simvastatin, Zoledronic Acid, Bortezomib, Bendamustine and Methylprednisolone for Relapsed/Refractory Myeloma	Bortezomib	Bendamustine, methylprednisolone (corticosteroid), simvastatin (statin) and zoledronic acid (bisphosphonate)	To evaluate the efficacy, safety and tolerability of the combination therapy of simvastatin and zoledronic acid (for reversal of drug resistance), with bortezomib, high-dose methylprednisolone and bendamustine (to reduce toxicity), in patients with MM who have relapsed or are refractory after bortezomib treatment.	This study has been withdrawn prior to enrollment.	

Supplementary table S1- Clinical trials of combination therapies with 20S CP inhibitors, involving patients with relapsed and/or refractory disease, and who were previously treated with a 20S CP inhibitor. Source: ClinicalTrials (https://clinicaltrials.gov/).

Study (code)	Title	20S CP inhibitor	Other drugs	Purpose	Observations	Ref.
NCT01372540	A Phase I Study of Arry-520 and Carfilzomib in Patients with Relapsed/Refractory Multiple Myeloma	Carfilzomib	Dexamethasone and filanesib. Anti-viral drugs (such as acyclovir, famciclovir, or valacyclovir) will also be administered to help prevent side effects, as well as filgrastim.	To evaluate the safety and to determine the highest tolerable dose of the combination of filanesib and carfilzomib in patients with MM or plasma cell leukemia. To learn if the combination of carfilzomib and filanesib can help control MM or plasma cell leukemia, and to learn if filanesib can help carfilzomib to work in patients who have built up a resistance to carfilzomib or carfilzomib alone has not helped to control the disease.	Study completed. The study included 2 parts (A and B) consisted of separate 3+3 dose-escalation of filanesib and carfilzomib, followed by a dose-expansion at the maximum tolerated dose. In part A, filanesib was dose-escalation in a 3+3 design (doses starting at 0.75 mg/m^2) with a fixed-dose of carfilzomib (20 mg/m^2 on firsts 2 days of cycle 1 and 27 mg/m^2 at subsequent infused) and dexamethasone 4 mg prior to each carfilzomib infusion, followed by a dose-expansion at the maximum tolerated dose of each drug. In part B of the study, filanesib was administered at a fixed-dose at the maximum tolerated dose established in part A, in combination with escalating doses of carfilzomib (20 mg/m^2 on firsts 2 days of cycle 1 and at 36 mg/m^2 or at higher dose at subsequent infused) and dexamethasone 4 mg prior to each carfilzomib infusion (dexamethasone dose was amended to 40 mg in mid-study of part B dose expansion). Between 1 March 2012 and 31 May 2016, a total of 64 participants with a median of 5 lines of prior therapy were enrolled and treated. Thirty-seven participants were refractory to bortezomib and 22 to carfilzomib.	[37]

Supplementary table S1- Clinical trials of combination therapies with 20S CP inhibitors, involving patients with relapsed and/or refractory disease, and who were previously treated with a 20S CP inhibitor. Source: ClinicalTrials (https://clinicaltrials.gov/).

Study (code)	Title	20S CP inhibitor	Other drugs	Purpose	Observations	Ref.
NCT01372540 (continuation)	A Phase I Study of Arry-520 and Carfilzomib in Patients with Relapsed/Refractory Multiple Myeloma	Carfilzomib	Dexamethasone and filanesib. Anti-viral drugs (such as acyclovir, famciclovir, or valacyclovir) will also be administered to help prevent side effects, as well as filgrastim.	To evaluate the safety and to determine the highest tolerable dose of the combination of filanesib and carfilzomib in patients with MM or plasma cell leukemia. To learn if the combination of carfilzomib and filanesib can help control MM or plasma cell leukemia, and to learn if filanesib can help carfilzomib to work in patients who have built up a resistance to carfilzomib or carfilzomib alone has not helped to control the disease.	From 63 response-evaluable participants in this study, the overall response rate was 37%. In all carfilzomib-refractory participants, the overall response rate was 14% (1 very good partial response and 2 partial responses) and 14% of the participants achieved a minimal response. In 9 carfilzomib-refractory participants, the overall response rate was 11% (1 partial response). In 5 carfilzomib-refractory participants, the overall response rate was 40% (1 very good partial response and 1 partial response) and 40% of the participants achieved a minimal response. Additionally, the median progression-free survival was 4.8 months for all patients, 2.2 months for carfilzomib-refractory participants and 8.4 months for carfilzomib non-refractory participants. The researchers concluded that this combinatory therapy can be combined safely at the maximum tolerated dose of individual drugs for relapsed and/or refractory MM patients. This result should be interpreted with caution due to the small sample size in these subsets, and they defended that the measured benefit of this regimen is uncertain without a randomized study.	[37]

Supplementary table S1- Clinical trials of combination therapies with 20S CP inhibitors, involving patients with relapsed and/or refractory disease, and who were previously treated with a 20S CP inhibitor. Source: ClinicalTrials (https://clinicaltrials.gov/).

Study (code)	Title	20S CP inhibitor	Other drugs	Purpose	Observations	Ref.
NCT01438177	A Phase II, Trial of Chloroquine in Combination with VELCADE and Cyclophosphamide in Patients with Relapsed and Refractory Myeloma	Bortezomib	Chloroquine (an autophagy inhibitor) and cyclophosphamide	To evaluate the efficacy and toxicity of these 3 drugs combined in patients with relapsed or refractory MM, and who should have progressed on a combination of bortezomib and cyclophosphamide.	Terminated study. Between October 2011 and April 2013, 11 patients were enrolled. One patient withdrew and 2 patients progressed prior to completion of cycle 1. Of the 8 evaluable patients who completed at least 2 cycles, 3 achieved a partial response, 1 showed stable disease and 4 progressed. Among the 3 patients who achieved a partial response, median duration of response was 4 months. The researchers concluded that the addition of chloroquine to bortezomib and cyclophosphamide is effective in overcoming proteasome inhibitor resistance in a significant fraction of heavily pretreated patients, with an acceptable toxicity profile.	[38]
NCT01445587	An Open-Label, Single Arm, Phase Ib/II Study of GSK2110183 in Subjects with Proteasome Inhibitor Refractory Multiple Myeloma	Bortezomib	GSK2110183 (an AKT inhibitor)	One of the purposes was to evaluate the clinical benefit of GSK2110183 and bortezomib as salvage therapy for patients with proteasome inhibitor refractory MM and who progressed on GSK2110183 monotherapy.	This study has been withdrawn prior to enrollment. Based on the clinical activity, not safety data, of the AKT inhibitor, GSK has decided to suspend further development of GSK2110183 as monotherapy.	

Supplementary table S1- Clinical trials of combination therapies with 20S CP inhibitors, involving patients with relapsed and/or refractory disease, and who were previously treated with a 20S CP inhibitor. Source: ClinicalTrials (https://clinicaltrials.gov/).

Study (code)	Title	20S CP inhibitor	Other drugs	Purpose	Observations	Ref.
NCT01485835	A Phase I Study of Ganetespib +/- Bortezomib in Patients with Relapsed and/or Refractory Multiple Myeloma	Bortezomib	Ganetespib (HSP90 inhibitor)	To evaluate what effects ganetespib and bortezomib have on relapsed and/or refractory MM. To determine the side effects of different dose levels of ganetespib when given alone and the effect it has on cancer alone. And to determine the side effects of ganetespib at different dose levels in combination with bortezomib and the effect the combination has on cancer.	Study completed. The participants included had diagnosis of relapsed or refractory MM and documentation of at least 2 prior therapies which must have included bortezomib and an immunomodulatory agent.	

Supplementary table S1- Clinical trials of combination therapies with 20S CP inhibitors, involving patients with relapsed and/or refractory disease, and who were previously treated with a 20S CP inhibitor. Source: ClinicalTrials (https://clinicaltrials.gov/).

Study (code)	Title	20S CP inhibitor	Other drugs	Purpose	Observations	Ref.

NCT01496118	Phase I/II Study of the Combination of Panobinostat and Carfilzomib in Patients with Relapsed/Refractory Multiple Myeloma	Carfilzomib	Panobinostat	For phase I, to determine the maximum tolerated dose and optimal doses of the combination of carfilzomib and panobinostat, in patients with relapsed/refractory MM. For phase II, assuming this combination is feasible, to evaluate the efficacy of this combination.	Study completed. Participants must have progressed during or after at least one previous bortezomib-containing treatment regimen. Results were published in 2015. No dose-limiting toxicities were observed, and, subsequently, the maximum tolerated dose was not reached. The overall response rate did not differ for the different subsets of populations including those with prior bortezomib exposure (70%) and those who were refractory to bortezomib (67%). As expected, patients who were refractory to bortezomib had shorter progression-free survival, time to progression and overall survival than the whole population of patients. This study concluded that the combination of carfilzomib and panobinostat is safe, tolerable and highly efficacious in patients with relapsed and/or refractory MM. After the original study was extended to investigate higher dose levels.	[39–41]

Supplementary table S1- Clinical trials of combination therapies with 20S CP inhibitors, involving patients with relapsed and/or refractory disease, and who were previously treated with a 20S CP inhibitor. Source: ClinicalTrials (https://clinicaltrials.gov/).

Study (code)	Title	20S CP inhibitor	Other drugs	Purpose	Observations	Ref.
NCT01496118 (continuation)	Phase I/II Study of the Combination of	Carfilzomib	Panobinostat	For phase I, to determine the maximum tolerated	For the comparison of the initial dose expansion and the subsequent dose	[39–41]

	Panobinostat and Carfilzomib in Patients with Relapsed/Refractory Multiple Myeloma			dose and optimal doses of the combination of carfilzomib and panobinostat, in patients with relapsed/refractory MM. For phase II, assuming this combination is feasible, to evaluate the efficacy of this combination.	expansion, 80 patients were enrolled, of them 99% were previously exposed to proteasome inhibitors and 46% were refractory to proteasome inhibitors. For all patients, 43% achieved a partial response and 32% achieved very good partial response or better. In the final results of the second dose expansion, 33 patients were enrolled, 100% exposed to prior proteasome inhibitors and 48.5% refractory to these inhibitors. For all evaluable patients, the overall response rate was 84.4% and clinical benefit rate was 90.6% (6.3% achieved complete response, 34.4% very good partial response and near complete response, 43.8% partial response and 6.3% minimal response). For the patients refractory to prior proteasome inhibitors, overall response rate was 80% (6.7% achieved complete response, 26.7% very good partial response and near complete response, 46.7% partial response and 13.3% minimal response) and the clinical benefit rate was 93.3%.	

Supplementary table S1- Clinical trials of combination therapies with 20S CP inhibitors, involving patients with relapsed and/or refractory disease, and who were previously treated with a 20S CP inhibitor. Source: ClinicalTrials (https://clinicaltrials.gov/).

Study (code)	Title	20S CP inhibitor	Other drugs	Purpose	Observations	Ref.
NCT01504776	A Phase I Study of Panobinostat in Combination with Bortezomib in the Treatment of Relapsed	Bortezomib	Panobinostat	To evaluate the safety and clinical efficacy of the combination of panobinostat plus	Study completed. Patients previously treated with bortezomib were included in the study.	

	and/or Refractory Mantle Cell Lymphoma			bortezomib for relapsed and/or refractory MCL.		

Supplementary table S1- Clinical trials of combination therapies with 20S CP inhibitors, involving patients with relapsed and/or refractory disease, and who were previously treated with a 20S CP inhibitor. Source: ClinicalTrials (https://clinicaltrials.gov/).

Study (code)	Title	20S CP inhibitor	Other drugs	Purpose	Observations	Ref.
NCT01522872	A Phase 1/2 Open-label Study to Assess the Safety, Tolerability and Preliminary Efficacy of TH-302, A Hypoxia-Activated Prodrug, and	Bortezomib	Dexamethasone and evofosfamide (an investigational 2-nitroimidazole hypoxia-activated prodrug of the DNA	To evaluate the safety, tolerability and preliminary efficacy of evofosfamide and dexamethasone with bortezomib in subjects	According to ClinicalTrials.gov, the recruitment status of this study is unknown.<br>In results reported in 2018, 31 participants were treated with evofosfamide and dexamethasone (arm	[42]

	Dexamethasone with or without Bortezomib or Pomalidomide in Subjects with Relapsed/Refractory Multiple Myeloma		alkylator bromo-isophosphoramide mustard)	with relapsed/refractory MM.	A), while 28 participants were treated with evofosfamide, dexamethasone and bortezomib (arm B). All the participants had prior bortezomib exposure, 61.0% relapsed to bortezomib, 30.5% refractory to bortezomib and 79.7% relapsed or refractory to bortezomib. For the participants of the arm A, the overall response rate was 12.9% (3 partial responses and 1 very good partial response), the clinical benefit rate was 19.4% (2 additional minimal responses), and 64.5% and 12.9% of the participants had, respectively, stable and progressive disease. One participant was not assessable. The median progression-free survival was 4.4 months and the median overall survival was 12.8 months.	

Supplementary table S1- Clinical trials of combination therapies with 20S CP inhibitors, involving patients with relapsed and/or refractory disease, and who were previously treated with a 20S CP inhibitor. Source: ClinicalTrials (https://clinicaltrials.gov/).

Study (code)	Title	20S CP inhibitor	Other drugs	Purpose	Observations	Ref.
NCT01522872 (continuation)	A Phase 1/2 Open-label Study to Assess the Safety, Tolerability and Preliminary Efficacy of TH-302, A Hypoxia-	Bortezomib	Dexamethasone and evofosfamide (an investigational 2-nitroimidazole hypoxia-activated	To evaluate the safety, tolerability and preliminary efficacy of evofosfamide and dexamethasone with	For the participants of the arm B, the overall response rate was 10.7% (1 complete response and 2 partial responses) and the clinical benefit rate was 17.9% (1 additional minimal	[42]

	Activated Prodrug, and Dexamethasone with or without Bortezomib or Pomalidomide in Subjects with Relapsed/Refractory Multiple Myeloma		prodrug of the DNA alkylator bromo-isophosphoramide mustard)	bortezomib in subjects with relapsed/refractory MM.	response), and 64.3% and 17.9% of the participants had, respectively, stable and progressive disease. One participant was not assessable. The median progression-free survival was 2.2 months and the median overall survival was 9.0 months. The difference of the progression-free survival and overall survival between the 2 arms was not statistically significant. This was likely due to differences in patient characteristics including the enrollment of more patients with high risk cytogenetics and a higher number of prior lines of therapy in arm B compared to arm A. The researchers concluded that this study demonstrates that evofosfamide alone or in combination with bortezomib is well tolerated and achieves stable disease and prolonged survival in a heavily pre-treated end- stage relapsed/refractory MM patients.	

Supplementary table S1- Clinical trials of combination therapies with 20S CP inhibitors, involving patients with relapsed and/or refractory disease, and who were previously treated with a 20S CP inhibitor. Source: ClinicalTrials (https://clinicaltrials.gov/).

Study (code)	Title	20S CP inhibitor	Other drugs	Purpose	Observations	Ref.
NCT01537861	A Pilot Study of G-CSF to Disrupt the Bone Marrow Microenvironment in Bortezomib-, Carfilzomib-, or IMID-Refractory Multiple Myeloma	Bortezomib or carfilzomib	Cyclophosphamide, dexamethasone, filgrastim and an immunomodulatory agent (thalidomide, lenalidomide or pomalidomide)	To explore the safety of the combination of filgrastim and bortezomib-, carfilzomib-, or immunomodulatory-based treatment regimens in patients with bortezomib-,	Based on the pre-clinical data the investigators hypothesized that filgrastim treatment in patients with MM will generate a 'hostile' bone marrow microenvironment for myeloma cells, depriving them of key support	

				carfilzomib-, or immunomodulatory-refractory myeloma and to generate correlative data for a subsequent larger study looking at the combination.	signals and rendering them more sensitive to chemotherapy. The study was terminated due to an unexpected toxicity (2 early deaths).	

Supplementary table S1- Clinical trials of combination therapies with 20S CP inhibitors, involving patients with relapsed and/or refractory disease, and who were previously treated with a 20S CP inhibitor. Source: ClinicalTrials (https://clinicaltrials.gov/).

Study (code)	Title	20S CP inhibitor	Other drugs	Purpose	Observations	Ref.
NCT01794507	A Phase 1b Study Evaluating the Safety and Pharmacokinetics of ABT-199 in Relapsed or Refractory Multiple Myeloma Subjects Who Are Receiving Bortezomib and Dexamethasone as Their Standard Therapy	Bortezomib	Dexamethasone and venetoclax (a BCL-2 inhibitor)	To assess the safety profile, characterize pharmacokinetics and determine the dosing schedule, maximum tolerated dose, and the recommended phase II dose of venetoclax when administered in subjects	Study completed. Results included 66 participants, 80% who were previously treated with bortezomib and 39% refractory to bortezomib. For the 39 participants non-refractory to bortezomib (including naïve participants), the overall response rate was 90% (8% stringent complete response, 20% complete response, 36%	[43]

				with relapsed /refractory MM who are receiving bortezomib and dexamethasone as their standard therapy.	very good partial response and 26% partial response). The median time to progression was 11.3 months and the median duration of response was 10.2 months. For the 26 participants refractory to bortezomib, the overall response rate was 31% (4% complete response, 4% very good partial response and 23% partial response). The median time to progression was 1.8 months and the median duration of response was 4.2 months. Because only a third of the participants refractory to bortezomib had an overall response, the researchers suggest that venetoclax cannot not fully overcome resistance to bortezomib.	

Supplementary table S1- Clinical trials of combination therapies with 20S CP inhibitors, involving patients with relapsed and/or refractory disease, and who were previously treated with a 20S CP inhibitor. Source: ClinicalTrials (https://clinicaltrials.gov/).

Study (code)	Title	20S CP inhibitor	Other drugs	Purpose	Observations	Ref.
NCT01813227	A Phase II Study of Carfilzomib in Relapsed Waldenström's Macroglobulinemia (WM) IST-CAR-531	Carfilzomib	Dexamethasone and rituximab	To evaluate the safety and effectiveness of carfilzomib (combined with dexamethasone, and with rituximab if less than a partial remission is achieved after 4 cycles) in participants with relapsed Waldenström's macroglobulinemia, which are bortezomib-	The recruitment status of this study is unknown.<br>In the results reported in 2018, 2 patients were enrolled. These patients were previously exposed to bortezomib but were not refractory to this inhibitor. The 2 patients received carfilzomib and dexamethasone but did not receive rituximab. For the treatment in study, one achieved as best overall response a very good partial response and there was	[44]

				naïve or bortezomib-exposed.	no progressive disease after 26.7 months of follow-up. The other patient achieved a minor response and the time to progression was 16.0 months.	
NCT01903811	A Phase II Randomized Study Comparing Two Doses of Carfilzomib (NSC-756640) with Dexamethasone for Multiple Myeloma Patients with Relapsed or Refractory Disease	Carfilzomib	Dexamethasone	To evaluate and compare the progression-free survival and response rates of 2 different doses of carfilzomib (low and high dose) with dexamethasone in relapsed and/or refractory MM patients.	Study completed. 121 participants were evaluated: 64 treated with low dose of carfilzomib and dexamethasone (50% of these participants were refractory to bortezomib), and 57 treated with high dose of carfilzomib and dexamethasone (49% of these participants were refractory to bortezomib). It was observed that the progression-free survival or overall survival were not significantly different between 2 arms in bortezomib refractoriness. There was a trend that high dose of carfilzomib benefitted participants with any number of prior lines and not bortezomib refractory.	[45]

Supplementary table S1- Clinical trials of combination therapies with 20S CP inhibitors, involving patients with relapsed and/or refractory disease, and who were previously treated with a 20S CP inhibitor. Source: ClinicalTrials (https://clinicaltrials.gov/).

Study (code)	Title	20S CP inhibitor	Other drugs	Purpose	Observations	Ref.
NCT01962792	A Multicenter Phase 1/2b Study of the Bruton's Tyrosine Kinase Inhibitor, Ibrutinib (PCI-32765), in Combination with Carfilzomib (Kyprolis™) in Subjects with Relapsed or Relapsed and Refractory Multiple Myeloma	Carfilzomib	Dexamethasone and ibrutinib (a Bruton's tyrosine kinase inhibitor)	The purpose of phase I is to establish the maximum tolerated dose of ibrutinib in combination with carfilzomib with or without dexamethasone, in patients with relapsed or relapsed and refractory MM, after receiving at least 2 previous therapies, including an	Study completed. The phase II sub-study cohort included subjects who must have received a regimen containing carfilzomib in combination with dexamethasone as their most recent line of therapy and who achieved less than a partial response following at least 4 cycles and had not exhibited progression disease, or had disease progression following an initial confirmed response	[46,47]

				immunomodulator and bortezomib and whom had either no response or documented disease progression to the most recent treatment regimen. The purpose of phase IIb is to evaluate the overall response rate when ibrutinib is administered in combination with carfilzomib and dexamethasone.	of minimal response or better to the combination.<br>In the results of the phase I trial published in 2018, 43 participants were enrolled: 8 treated with ibrutinib and carfilzomib and 35 treated with ibrutinib, carfilzomib and dexamethasone. Seventy-four percent were refractory to bortezomib and 9% previously were treated with carfilzomib. Moreover, 88% of patients were refractory to their last line of therapy, of which 53% included bortezomib, all of whom were actively progressing post-bortezomib. Forty-two patients treated with the combination of carfilzomib and ibrutinib and with or without dexamethasone, were evaluable for response.	

Supplementary table S1- Clinical trials of combination therapies with 20S CP inhibitors, involving patients with relapsed and/or refractory disease, and who were previously treated with a 20S CP inhibitor. Source: ClinicalTrials (https://clinicaltrials.gov/).

Study (code)	Title	20S CP inhibitor	Other drugs	Purpose	Observations	Ref.
NCT01962792 (continuation)	A Multicenter Phase 1/2b Study of the Bruton's Tyrosine Kinase Inhibitor, Ibrutinib (PCI-32765), in Combination with Carfilzomib (Kyprolis™) in Subjects with Relapsed or Relapsed and Refractory Multiple Myeloma	Carfilzomib	Dexamethasone and ibrutinib (a Bruton's tyrosine kinase inhibitor)	The purpose of phase I is to establish the maximum tolerated dose of ibrutinib in combination with carfilzomib with or without dexamethasone, in patients with relapsed or relapsed and refractory MM, after receiving at least 2 previous therapies,	In 42 participants evaluated, the overall response rate was 67% (1 stringent complete response, 9 very good partial response and 18 partial response), the clinical benefit rate was 76% (4 additional minimal responses) and the median duration of response for patients who achieved partial response or better was 12.9 months. For the patients refractory to bortezomib and treated	[46,47]

				including an immunomodulator and bortezomib and whom had either no response or documented disease progression to the most recent treatment regimen. The purpose of phase IIb is to evaluate the overall response rate when ibrutinib is administered in combination with carfilzomib and dexamethasone.	with ibrutinib, carfilzomib and dexamethasone, the overall response rate was 73% and the median duration of response was 9.1 months. For all patients treated with the combination of the 3 drugs (refractory or not to bortezomib) the overall response rate was 71%.<br>Final results from participants who received the recommended phase 2 dose (which was determined in phase I), were published in 2020: 18 participants in phase I and 41 in phase IIb (including 69% refractory to bortezomib), however only 57 were considered response evaluable.	

Supplementary table S1- Clinical trials of combination therapies with 20S CP inhibitors, involving patients with relapsed and/or refractory disease, and who were previously treated with a 20S CP inhibitor. Source: ClinicalTrials (https://clinicaltrials.gov/).

Study (code)	Title	20S CP inhibitor	Other drugs	Purpose	Observations	Ref.
NCT01962792 (continuation)	A Multicenter Phase 1/2b Study of the Bruton's Tyrosine Kinase Inhibitor, Ibrutinib (PCI-32765), in Combination with Carfilzomib (Kyprolis™) in Subjects with Relapsed or Relapsed and Refractory Multiple Myeloma	Carfilzomib	Dexamethasone and ibrutinib (a Bruton's tyrosine kinase inhibitor)	The purpose of phase I is to establish the maximum tolerated dose of ibrutinib in combination with carfilzomib with or without dexamethasone, in patients with relapsed or relapsed and refractory MM, after receiving at least 2 previous therapies, including an	The overall response rate was 71% (2 stringent complete response, 12 very good partial response and 26 partial response), reported by the researchers like comparable to regimens used to treat relapsed/refractory MM containing bortezomib (63-84%). The clinical benefit rate was 78%, the median duration of response was 6.5 months and the median progression-free survival was 7.4 months. The researchers found	[46,47]

				immunomodulator and bortezomib and whom had either no response or documented disease progression to the most recent treatment regimen. The purpose of phase IIb is to evaluate the overall response rate when ibrutinib is administered in combination with carfilzomib and dexamethasone.	that carfilzomib can be used with acceptable safety combined with dexamethasone and ibrutinib and this regimen could be used as a bridge therapy in patients with relapsed/refractory MM and previously treated with bortezomib.	

Supplementary table S1- Clinical trials of combination therapies with 20S CP inhibitors, involving patients with relapsed and/or refractory disease, and who were previously treated with a 20S CP inhibitor. Source: ClinicalTrials (https://clinicaltrials.gov/).

Study (code)	Title	20S CP inhibitor	Other drugs	Purpose	Observations	Ref.
NCT01965353	A Phase I Open Label Study Of Panobinostat In Combination with Lenalidomide, Bortezomib, And Dexamethasone In Patients with Relapsed And Relapsed/Refractory Multiple Myeloma	Bortezomib	Dexamethasone, lenalidomide and panobinostat	To establish the maximum tolerated dose of panobinostat in combination with bortezomib plus dexamethasone plus lenalidomide, in patients with relapsed or relapsed/refractory MM, heavily pretreated, particularly in patients refractory to proteasome	Study completed. Twenty participants were enrolled: 75% bortezomib-refractory and 50% refractory to bortezomib and lenalidomide. Among the 18 response-evaluable, the overall response rate was 44%, the clinical benefit rate was 61%, the median duration of response was 9.2 months and the median progression-free survival was 7.4 months. Among the 10 response-evaluable from dual-refractory participants, the overall response rate	[48]

				inhibitors and/or immunomodulatory drugs. To evaluate the safety and the preliminary effectiveness of this combination therapy.	was 30%, the clinical benefit rate was 50%, the median duration of response was 22.1 months and the median progression-free survival was 22.9 months.	

Supplementary table S1- Clinical trials of combination therapies with 20S CP inhibitors, involving patients with relapsed and/or refractory disease, and who were previously treated with a 20S CP inhibitor. Source: ClinicalTrials (https://clinicaltrials.gov/).

Study (code)	Title	20S CP inhibitor	Other drugs	Purpose	Observations	Ref.
NCT01989325	A Study of Filanesib (ARRY-520) and Carfilzomib in Patients with Advanced Multiple Myeloma	Carfilzomib	Dexamethasone, filanesib, and filgrastim (prophylactic)	To evaluate the safety and the effectiveness of both single-agent carfilzomib and carfilzomib and filanesib in patients with refractory MM refractory to last regimen and who must have received at least 2 prior treatment regimens, including bortezomib and an immunomodulatory agent	Patients would be allowed to crossover from single-agent carfilzomib to carfilzomib and filanesib if disease progression occurs. A phase II completed study. Results reported in 2015 included 77 participants: 25 treated only with carfilzomib (arm A) and 52 with carfilzomib and filanesib (arm B). Sixty-eight participants were evaluable for response (results of 9 participants of arm B were not included). For the arm A, the overall response rate was 24% (6 partial	[49]

				(no prior treatment with carfilzomib was allowed.).	response), the clinical benefit rate was 28% (1 additional minor response), the disease control rate was 52%, the median progression-free survival was 3.7 months and the median duration of response for patients who achieved partial response or better was not reached. For the 15 participants refractory to bortezomib and immunomodulatory agents randomized to this arm, the overall response rate was 33%, the clinical benefit rate was 40%, and the median duration of response for patients who achieved partial response or better was not reached.	

Supplementary table S1- Clinical trials of combination therapies with 20S CP inhibitors, involving patients with relapsed and/or refractory disease, and who were previously treated with a 20S CP inhibitor. Source: ClinicalTrials (https://clinicaltrials.gov/).

Study (code)	Title	20S CP inhibitor	Other drugs	Purpose	Observations	Ref.
NCT01989325 (continuation)	A Study of Filanesib (ARRY-520) and Carfilzomib in Patients with Advanced Multiple Myeloma	Carfilzomib	Dexamethasone, filanesib, and filgrastim (prophylactic)	To evaluate the safety and the effectiveness of both single-agent carfilzomib and carfilzomib and filanesib in patients with refractory MM refractory to last regimen and who must have received at least 2 prior treatment regimens, including bortezomib and an immunomodulatory agent (no prior treatment with carfilzomib was allowed.).	For the arm B, the overall response rate was 28% (3 very good partial responses and 9 partial responses), the clinical benefit rate was 33% (2 additional minor response), the disease control rate was 65%, the median progression-free survival was 8.5 months and the median duration of response for patients who achieved partial response or better was 11.2 months. For the 27 participants refractory to bortezomib and immunomodulatory agents randomized to this arm, the overall response rate was 33%, the clinical benefit rate was 37%, and the median duration of response for	[49]

					patients who achieved partial response or better was 11.2 months.	

Supplementary table S1- Clinical trials of combination therapies with 20S CP inhibitors, involving patients with relapsed and/or refractory disease, and who were previously treated with a 20S CP inhibitor. Source: ClinicalTrials (https://clinicaltrials.gov/).

Study (code)	Title	20S CP inhibitor	Other drugs	Purpose	Observations	Ref.
NCT01999335	Phase 1b/3 Multicenter Study of Oprozomib, Pomalidomide, and Dexamethasone in Primary Refractory or Relapsed and Refractory Multiple Myeloma Subjects	Oprozomib	Dexamethasone and pomalidomide	The purpose of phase I of the study is to determine the maximum tolerated dose of oprozomib using 2 different schedules (5/14 and 2/7 schedules) when in combination with pomalidomide and dexamethasone, the recommended phase III dose and schedule, and assess the safety, tolerability and activity of oprozomib in combination with pomalidomide and dexamethasone in subjects with primary refractory or	Study completed. Bortezomib- and carfilzomib-refractory patients were included in initial results of phase Ib. In this study 3+3 dose-escalation schema and dose-expansion were used to determine the maximum tolerated dose on 2 schedules, with 150 and 210 mg of starting dose for oprozomib in the 5/14 and 2/7 schedules, respectively; 4 mg for pomalidomide in both schedules, and with a fixed-dose dexamethasone (20 mg). Thirty-three participants were enrolled and 31 of them were treated (4 and 27 in 5/14 and 2/7 schedule, respectively). Thirty participants were previously exposed to bortezomib, 15 were previously exposed to carfilzomib;	[50]

				relapsed and refractory MM. The purpose of phase III of the study is to compare the key outcome measures for subjects with primary refractory or relapsed and refractory MM who are randomized to either oprozomib or placebo in combination with pomalidomide and dexamethasone.	26 and 11 were refractory to bortezomib and carfilzomib, respectively, in any prior therapy. Oprozomib maximum tolerated dose was not defined in dose-escalation for either schedule, however 210 mg dose (with 4 mg pomalidomide) was selected in the expansion cohort based on the safety and tolerability profile.	

Supplementary table S1- Clinical trials of combination therapies with 20S CP inhibitors, involving patients with relapsed and/or refractory disease, and who were previously treated with a 20S CP inhibitor. Source: ClinicalTrials (https://clinicaltrials.gov/).

Study (code)	Title	20S CP inhibitor	Other drugs	Purpose	Observations	Ref.
NCT01999335 (continuation)	Phase 1b/3 Multicenter Study of Oprozomib, Pomalidomide, and Dexamethasone in Primary Refractory or Relapsed and Refractory Multiple Myeloma Subjects	Oprozomib	Dexamethasone and pomalidomide	The purpose of phase I of the study is to determine the maximum tolerated dose of oprozomib using 2 different schedules (5/14 and 2/7 schedules) when in combination with pomalidomide and dexamethasone, the recommended phase III dose and schedule, and assess the safety, tolerability and activity of oprozomib in combination with pomalidomide and dexamethasone in subjects with primary refractory or	In 4 participants included in the efficacy analysis of 5/14 schedule, who were all bortezomib-refractory and with prior carfilzomib exposure, the overall response rate was 50% (2 partial responses); 25% of the participants achieved a stable disease and 25% of the participants achieved a missing response. In 17 participants included in the efficacy analysis of 2/7 schedule at 210 mg of oprozomib and 4 mg of pomalidomide, and 82% and 29% of this subgroup of participants were refractory to bortezomib and carfilzomib, respectively. The overall response rate was 70.6% (8 very good partial responses and 4 partial responses,	[50]

				relapsed and refractory MM. The purpose of phase III of the study is to compare the key outcome measures for subjects with primary refractory or relapsed and refractory MM who are randomized to either oprozomib or placebo in combination with pomalidomide and dexamethasone.	including 4 of 5 participants refractory to carfilzomib in any prior line), 17.6% of the participants achieved a stable disease and 11.8% of the participants achieved progressive disease. In 10 participants included in the efficacy analysis of 2/7 schedule at 240 mg of oprozomib and 4 mg of pomalidomide, the overall response rate was 50% (3 very good partial responses and 2 partial responses), 10.0, 20.0 and 20.0% of the participants achieved a minimal response, stable disease and missing response, respectively.	

Supplementary table S1- Clinical trials of combination therapies with 20S CP inhibitors, involving patients with relapsed and/or refractory disease, and who were previously treated with a 20S CP inhibitor. Source: ClinicalTrials (https://clinicaltrials.gov/).

Study (code)	Title	20S CP inhibitor	Other drugs	Purpose	Observations	Ref.
NCT01999335 (continuation)	Phase 1b/3 Multicenter Study of Oprozomib, Pomalidomide, and Dexamethasone in Primary Refractory or Relapsed and Refractory Multiple Myeloma Subjects	Oprozomib	Dexamethasone and pomalidomide	The purpose of phase I of the study is to determine the maximum tolerated dose of oprozomib using 2 different schedules (5/14 and 2/7 schedules) when in combination with pomalidomide and dexamethasone, the recommended phase III dose and schedule, and assess the safety, tolerability and activity of oprozomib in combination with pomalidomide and dexamethasone in subjects with primary refractory or	Only for the 5/14 schedule the median duration of response was estimable (6.2 months). The researchers concluded that the regimen explored (2/7 schedule) have promising activity for patients with relapsed or refractory MM.	[50]

				relapsed and refractory MM. The purpose of phase III of the study is to compare the key outcome measures for subjects with primary refractory or relapsed and refractory MM who are randomized to either oprozomib or placebo in combination with pomalidomide and dexamethasone.		

Supplementary table S1- Clinical trials of combination therapies with 20S CP inhibitors, involving patients with relapsed and/or refractory disease, and who were previously treated with a 20S CP inhibitor. Source: ClinicalTrials (https://clinicaltrials.gov/).

Study (code)	Title	20S CP inhibitor	Other drugs	Purpose	Observations	Ref.
NCT01998971	An Open-Label, Multicenter, Phase 1b Study of JNJ-54767414 (HuMax CD38) (Anti-CD38 Monoclonal Antibody) in Combination with Backbone Regimens for the Treatment of Subjects with Multiple Myeloma	Carfilzomib	Daratumumab (human-specific anti-CD38 monoclonal antibody) and dexamethasone	Mainly to evaluate the safety, tolerability, and dose regimen of daratumumab plus carfilzomib and dexamethasone in patients with relapsed or refractory MM after 1 to 3 prior lines of therapy.	This study is ongoing, but not recruiting participants. Results reported in 2019 included 82 participants whose response was analyzed. All participants were previously treated with bortezomib. Analyzing the response of all participants, the overall response rate was 84%, with 33% achieving a best response of complete response or better. For the 25 participants to bortezomib analyzed, the overall response rate was 84%, with 20% achieving a best response of complete response or better. For the 24 participants refractory to a proteasome inhibitor and an	[51,52]

					immunomodulator drug analyzed, the overall response rate was 83%.	
NCT02020941	A Phase 2 Study of Carfilzomib and Bone Metabolism in Patients with Multiple Myeloma in First Relapse or Refractory to First Line Therapy	Carfilzomib	Dexamethasone (after 4 cycles of treatment with carfilzomib, patients achieving less than partial response also would receive dexamethasone)	Mainly to evaluate the efficacy of carfilzomib in patients with MM in first relapse or refractory to first-line therapy, which should include either an immunomodulatory agent or a proteasome inhibitor.	This study has been terminated (slow accrual).	

Supplementary table S1- Clinical trials of combination therapies with 20S CP inhibitors, involving patients with relapsed and/or refractory disease, and who were previously treated with a 20S CP inhibitor. Source: ClinicalTrials (https://clinicaltrials.gov/).

Study (code)	Title	20S CP inhibitor	Other drugs	Purpose	Observations	Ref.
NCT02027220	Phase II Study of G-CSF, Bortezomib, Cyclophosphamide and Dexamethasone in Patients with Multiple Myeloma	Bortezomib	Cyclophosphamide, dexamethasone and G-CSF (granulocyte colony-stimulating factor, possesses the ability to mobilize the plasma cells to detach from myeloma niche, so as to promote drug sensitivity)	To study how well the combination of G-CSF, bortezomib, cyclophosphamide and dexamethasone works in treating patients with MM. It will evaluate its efficacy and adverse effects.	The recruitment status of this study is unknown. One of the inclusion criteria was patients with relapsed or bortezomib resistant MM, who did not receive bortezomib during the last line of therapy prior to this study. In the initial results reported, those included were 19 participants with primary MM who had not received prior therapy and 3 participants with refractory disease who received regimens including bortezomib. For 19 participants with primary MM, the overall response rate was 89.5% after 1 cycle; for 10 participants who received 4 cycles the overall response rate was 100% (7 near	[53]

					complete responses/complete responses, 2 very good partial responses and 1 partial response), and for 3 participants treated with 6 cycles all achieved complete response. Of 3 refractory participants, the overall response rate was 67% (2 partial responses) after 1 cycle and 100% after 3 cycles (2 complete responses and 1 partial response).	

Supplementary table S1- Clinical trials of combination therapies with 20S CP inhibitors, involving patients with relapsed and/or refractory disease, and who were previously treated with a 20S CP inhibitor. Source: ClinicalTrials (https://clinicaltrials.gov/).

Study (code)	Title	20S CP inhibitor	Other drugs	Purpose	Observations	Ref.
NCT02056756	Carfilzomib in combination with bendamustine and dexamethasone in refractory or relapsed multiple myeloma - a multicenter phase Ib/II trial of the European myeloma network trialist group (EMNTG)	Carfilzomib	Bendamustine and dexamethasone	To determine the safety and efficacy of the novel salvage regimen (CBd) followed by a carfilzomib maintenance in patients with relapsed or/and refractory MM after failure of 2 or more treatment regimens, allowing previous bortezomib treatment.	This study is ongoing, but not recruiting participants.<br>Results reported in 2021 included 63 participants whose response was analyzed. The median progression-free survival was 11.6 months. No significant difference in progression-free survival was registered for the participants who relapsed on or were refractory to bortezomib,	[54]
NCT02057640	A Phase I/II Study of MLN9708 (Ixazomib) in Combination with Panobinostat and Dexamethasone in Patients with Relapsed or	Ixazomib	Dexamethasone and panobinostat	To evaluate the safety and tolerability of ixazomib in combination with panobinostat and dexamethasone among patients with relapsed or	Study completed.	

	Refractory Multiple Myeloma			refractory MM and who must have previously received therapy with a proteasome inhibitor and an immunomodulator. The secondary purposes are to analyse the response and clinical benefit of this treatment and the progression-free survival and overall survival of study participants.		

Supplementary table S1- Clinical trials of combination therapies with 20S CP inhibitors, involving patients with relapsed and/or refractory disease, and who were previously treated with a 20S CP inhibitor. Source: ClinicalTrials (https://clinicaltrials.gov/).

Study (code)	Title	20S CP inhibitor	Other drugs	Purpose	Observations	Ref.
NCT02095834	A Single Center Phase Ib Study of Carfilzomib, Bendamustine and Dexamethasone in Subjects with Relapsed/Refractory Multiple Myeloma	Carfilzomib	Bendamustine and dexamethasone	To determine the maximum tolerable dose and to evaluate the efficacy and safety of carfilzomib when given in combination with bendamustine and dexamethasone in patients with relapsed/refractory MM after at least one prior line of therapy and who should have received at least one prior novel agent (immunomodulatory agents or proteasome inhibitors).	Study completed. 17 response-evaluable patients were included: 12 refractory to bortezomib, 6 refractory to carfilzomib and 12 to both immunomodulatory drugs and proteasome inhibitors. For all participants and dual refractory participants, the median progression-free survival was 15.1 and 11.1 months, respectively. Despite the limitations of the study, it suggests that this combination therapy has the potential to treat heavily pretreated patients with a median of 4 lines of prior therapy, including carfilzomib-refractory and dual-refractory patients.	[55]

Supplementary table S1- Clinical trials of combination therapies with 20S CP inhibitors, involving patients with relapsed and/or refractory disease, and who were previously treated with a 20S CP inhibitor. Source: ClinicalTrials (https://clinicaltrials.gov/).

Study (code)	Title	20S CP inhibitor	Other drugs	Purpose	Observations	Ref.
NCT02100657	Phase I Study of Plitidepsin (Aplidin®) in Combination with Bortezomib and Dexamethasone in Patients with Relapsed and/or Refractory Multiple Myeloma	Bortezomib	Dexamethasone and plitidepsin (anticancer agent which blocks the proto-oncogene eEF1A2 over-expressed in different tumor types, including the MM)	To determine the recommended dose of plitidepsin in combination with bortezomib and dexamethasone in patients with relapsed and/or refractory MM. To evaluate the efficacy, the safety, the tolerability and to study the pharmacokinetics and pharmacodynamics of plitidepsin in combination with bortezomib and dexamethasone.	Study completed. The participants must have had received at least one previous treatment line of induction, chemotherapy, chemotherapy and transplantation or previous treatment with bortezomib or another proteasome drug. In the results reported in 2016, 83% of the 18 patients evaluable for efficacy had received bortezomib and lenalidomide. For the 18 patients evaluable, the overall response rate was 56% (2 complete responses, 4 very good partial responses and 4 partial responses) and the clinical benefit rate was 72% (3 additional minimal responses). One of the patients who achieved a partial response was triple refractory to bortezomib, lenalidomide and pomalidomide.	[56]

Supplementary table S1- Clinical trials of combination therapies with 20S CP inhibitors, involving patients with relapsed and/or refractory disease, and who were previously treated with a 20S CP inhibitor. Source: ClinicalTrials (https://clinicaltrials.gov/).

Study (code)	Title	20S CP inhibitor	Other drugs	Purpose	Observations	Ref.
NCT02101944	Pilot Trial Evaluating Viral Protein Production from the Combination of Reolysin and Carfilzomib in Multiple Myeloma	Carfilzomib	Dexamethasone and reolysin (wild-type reovirus)	To determine safety, tolerability and preliminary efficacy, and define the maximum tolerated dose of reolysin, carfilzomib and dexamethasone in patients with relapsed MM.	This study is ongoing, but not recruiting participants. Preliminary results reported in 2020, included 6 carfilzomib-refractory participants. 2 patients achieved partial responses with concomitant CD8 and NK cell recruitment, PD-L1 upregulation, activated caspase-3 expression and increased viral protein production within the myeloma cells. These patients demonstrated mild to severe signs and symptoms consistent with secondary haemophagocytic lymphohistiocytosis.	[57]

Supplementary table S1- Clinical trials of combination therapies with 20S CP inhibitors, involving patients with relapsed and/or refractory disease, and who were previously treated with a 20S CP inhibitor. Source: ClinicalTrials (https://clinicaltrials.gov/).

Study (code)	Title	20S CP inhibitor	Other drugs	Purpose	Observations	Ref.
NCT02103335	A Phase 1, Multicenter, Open-label, Dose-Escalation Combination Study of Pomalidomide, Marizomib, and Low-Dose Dexamethasone in Subjects with Relapsed and Refractory Multiple Myeloma	Marizomib	Dexamethasone and pomalidomide	The primary objective of this study is to determine the best drug dosing levels for this three-drug combination, including the highest safe doses and/or the recommended doses for future clinical studies of this drug combination. The secondary purposes of this study are to determine the safety of this drug combination and its effectiveness in treating relapsed or refractory MM, in patients who previously received 1 or more lines of anti-myeloma therapy that must have included both lenalidomide and bortezomib (either separately or in combination).	Study completed. In this trial 36 patients were evaluable for efficacy and the overall response rate was 53% (2 very good partial responses and 17 partial responses) and the clinical benefit rate was 64% (4 additional minimal responses). Ten patients achieved stable disease and 3 had progressive disease. For the 11 patients previously exposed to carfilzomib the overall response rate was 82% and the clinical benefit rate was 91%. For the 10 patients refractory to carfilzomib the overall response rate was 80% (8 partial responses) and the clinical benefit rate was 90%. For the 7 patients refractory to carfilzomib in last regimen the overall response rate and the clinical benefit rate were 86%. For the 21 patients refractory to bortezomib the overall response rate was 57% and the clinical benefit rate was 62%. For the 7 patients refractory to bortezomib in last regimen the overall response rate was 43% and the clinical benefit rate was 57%. For the 18 patients double refractory to bortezomib and lenalidomide the overall response rate	[58]

					was 56% and the clinical benefit rate was 67%.	

Supplementary table S1- Clinical trials of combination therapies with 20S CP inhibitors, involving patients with relapsed and/or refractory disease, and who were previously treated with a 20S CP inhibitor. Source: ClinicalTrials (https://clinicaltrials.gov/).

Study (code)	Title	20S CP inhibitor	Other drugs	Purpose	Observations	Ref.
NCT02103335 (continuation)	A Phase 1, Multicenter, Open-label, Dose-Escalation Combination Study of Pomalidomide, Marizomib, and Low-Dose Dexamethasone in Subjects with Relapsed and Refractory Multiple Myeloma	Marizomib	Dexamethasone and pomalidomide	The primary objective of this study is to determine the best drug dosing levels for this three-drug combination, including the highest safe doses and/or the recommended doses for future clinical studies of this drug combination. The secondary purposes of this study are to determine the safety of this drug combination and its effectiveness in treating relapsed or refractory MM, in patients who previously received 1 or more lines of anti-myeloma therapy that must have included both lenalidomide and bortezomib (either	For the 7 patients triple refractory to bortezomib, carfilzomib and lenalidomide the overall response rate was 71% and the clinical benefit rate was 87%. The median duration of response, median progression-free survival and median overall survival were not significantly different in double and triple refractory patients compared to the total study population.	[58]

				separately or in combination).		

Supplementary table S1- Clinical trials of combination therapies with 20S CP inhibitors, involving patients with relapsed and/or refractory disease, and who were previously treated with a 20S CP inhibitor. Source: ClinicalTrials (https://clinicaltrials.gov/).

Study (code)	Title	20S CP inhibitor	Other drugs	Purpose	Observations	Ref.
NCT02119468	Phase I/II Trial of MLN9708 Plus Pomalidomide and Dexamethasone for Relapsed or Relapsed Refractory Multiple Myeloma	Ixazomib	Dexamethasone and pomalidomide	The purposes of phase I are to evaluate the safety and to determine the recommended phase II dose of ixazomib combined with pomalidomide and dexamethasone, in patients with relapsed or relapsed/refractory MM and who must have had therapy with a proteasome inhibitor and lenalidomide and be refractory to lenalidomide. The purpose of phase II is to evaluate the toxicity and the efficacy of this drug combination.	This study is ongoing, but not recruiting participants. Thirty-two participants were enrolled between July 2014 and March 2016. From these participants 25 were evaluable and treated with the recommended phase II dose of ixazomib combined with pomalidomide and dexamethasone. All 25 participants were refractory to lenalidomide and previously received bortezomib therapy with 14 refractory to this inhibitor, 5 participants were previously exposed to carfilzomib and 2 were triple refractory to bortezomib, carfilzomib and lenalidomide. None of the participants were previously treated with ixazomib. For the 25 participants the overall response rate was 48% (5 very good partial responses and 7 partial responses), the clinical benefit rate was 76% (1 additional minimal response and 6 additional participants had stable	[59]

					disease), and 24% had progressive disease. The median duration of response for patients who achieved partial response or better was 9.2 months and the median progression-free survival was 8.6 months.	

Supplementary table S1- Clinical trials of combination therapies with 20S CP inhibitors, involving patients with relapsed and/or refractory disease, and who were previously treated with a 20S CP inhibitor. Source: ClinicalTrials (https://clinicaltrials.gov/).

Study (code)	Title	20S CP inhibitor	Other drugs	Purpose	Observations	Ref.
NCT02119468 (continuation)	Phase I/II Trial of MLN9708 Plus Pomalidomide and Dexamethasone for Relapsed or Relapsed Refractory Multiple Myeloma	Ixazomib	Dexamethasone and pomalidomide	The purposes of phase I are to evaluate the safety and to determine the recommended phase II dose of ixazomib combined with pomalidomide and dexamethasone, in patients with relapsed or relapsed/refractory MM and who must have had therapy with a proteasome inhibitor and lenalidomide and be refractory to lenalidomide. The purpose of phase II is to evaluate the toxicity and the efficacy of this drug combination.	In the participants refractory to bortezomib, the overall response rate was 29% (1 very good partial response and 3 partial responses), the clinical benefit rate was 71% (1 additional minimal response and 5 additional participants had stable disease), and 29% had progressive disease.	[59]

Supplementary table S1- Clinical trials of combination therapies with 20S CP inhibitors, involving patients with relapsed and/or refractory disease, and who were previously treated with a 20S CP inhibitor. Source: ClinicalTrials (https://clinicaltrials.gov/).

Study (code)	Title	20S CP inhibitor	Other drugs	Purpose	Observations	Ref.
NCT02145715	A Phase I/IIa Trial of VTD-panobinostat Treatment and Panobinostat Maintenance in Relapsed and Relapsed/Refractory Multiple Myeloma Patients	Bortezomib	Dexamethasone, panobinostat, and thalidomide	To determine the maximum tolerated dose and estimated response rates of panobinostat, administered in combination with bortezomib, thalidomide and dexamethasone, in patients with relapsed and relapsed/refractory MM.	According to ClinicalTrials.gov, the recruitment status of this study is unknown. However, results were published. Because the trial excluded those refractory to bortezomib, the potential ability of panobinostat to overcome bortezomib resistance was not evaluated. In the 46 participants evaluable, the participants with partial response or better were similar between the 33 participants with prior bortezomib exposure and the 13 participants naïve to bortezomib, showing effectiveness for participants who were previously treated with bortezomib. The overall response was 91% for the 46 participants evaluated, 91% for the participants with prior bortezomib exposure, 92% for the participants naïve to bortezomib, 88% for the 24 participants previously treated with proteasome inhibitor and immunomodulatory drugs, 95% for the 22 participants naïve to proteasome inhibitor and immunomodulatory drugs	[60,61]

					and 75% for the 8 participants treated with 2 or more previous lines of treatment including bortezomib and a proteasome inhibitor or immunomodulatory drug.	

Supplementary table S1- Clinical trials of combination therapies with 20S CP inhibitors, involving patients with relapsed and/or refractory disease, and who were previously treated with a 20S CP inhibitor. Source: ClinicalTrials (https://clinicaltrials.gov/).

Study (code)	Title	20S CP inhibitor	Other drugs	Purpose	Observations	Ref.
NCT02188537	Nelfinavir as Bortezomib-sensitizing Drug in Patients with Proteasome Inhibitor-nonresponsive Myeloma. A Multicenter Phase II Trial	Bortezomib	Dexamethasone and nelfinavir	To decide whether nelfinavir, bortezomib and dexamethasone combined have sufficient activity in proteasome inhibitor-resistant myeloma patients to merit further clinical investigation in a prospective controlled trial. To collect myeloma cell samples from proteasome inhibitor-resistant myeloma patients for the assessment of the biology of proteasome inhibitor resistance and the identification of predictive markers for response to nelfinavir-based antimyeloma therapy.	Study completed. Thirty-four bortezomib-refractory patients were enrolled. The objective response rate was 65% (17 partial responses and 5 very good partial responses). 9% of the patients achieved a minimal response, 12% achieved a stable disease and 9% had progressive disease. The median progression-free survival was 12 weeks overall and 16 weeks for patients reaching partial response or better. The median overall survival was 12 months. For the 27 patients double refractory to bortezomib and lenalidomide the objective response rate was 70%. For the 15 patients double refractory to bortezomib and pomalidomide the objective response rate was 60%. For the 13 patients triple refractory to bortezomib, lenalidomide and pomalidomide the objective response rate was 62%.	[62]

Supplementary table S1- Clinical trials of combination therapies with 20S CP inhibitors, involving patients with relapsed and/or refractory disease, and who were previously treated with a 20S CP inhibitor. Source: ClinicalTrials (https://clinicaltrials.gov/).

Study (code)	Title	20S CP inhibitor	Other drugs	Purpose	Observations	Ref.
NCT02199665	A Phase I Study of the Combination of a Selective Inhibitor of Nuclear Export (SINE), Selinexor with Carfilzomib and Dexamethasone in Patients with Relapsed or Relapsed/Refractory Multiple Myeloma	Carfilzomib	Dexamethasone and selinexor (a selective inhibitor of nuclear export XPO1/CRM1)	To evaluate the efficacy, the safety and the tolerability and to determine the maximum tolerated dose and the recommended phase II dose of the combination of selinexor, carfilzomib, and dexamethasone in relapsed and relapsed/refractory MM.	This study is currently recruiting participants. Subjects must have been treated with at least 2 prior therapies including a proteasome inhibitor (including carfilzomib) and a cereblon-binding agent.<br>Between July 2014 and September 2016, 21 participants were enrolled and had received a median of 4 prior lines of therapy, including carfilzomib and bortezomib in 95% of cases. Ninety-five percent of participants were refractory to carfilzomib and 52% to bortezomib. A phase I dose-escalation trial of twice-weekly selinexor in combination with carfilzomib and dexamethasone to determine maximum tolerated dose was performed, with doses starting of 30 mg to selinexor, 20/27 mg/m^2 to carfilzomib and a fixed-dose of 20 mg to dexamethasone (10 mg after 4 completed cycles). The majority of participants achieved disease control after 1 cycle, with a overall response rate of 38% and clinical benefit rate of 67%.	[63]

Supplementary table S1- Clinical trials of combination therapies with 20S CP inhibitors, involving patients with relapsed and/or refractory disease, and who were previously treated with a 20S CP inhibitor. Source: ClinicalTrials (https://clinicaltrials.gov/).

Study (code)	Title	20S CP inhibitor	Other drugs	Purpose	Observations	Ref.
NCT02199665 (continuation)	A Phase I Study of the Combination of a Selective Inhibitor of Nuclear Export (SINE), Selinexor with Carfilzomib and Dexamethasone in Patients with Relapsed or Relapsed/Refractory Multiple Myeloma	Carfilzomib	Dexamethasone and selinexor (a selective inhibitor of nuclear export XPO1/CRM1)	To evaluate the efficacy, the safety and the tolerability and to determine the maximum tolerated dose and the recommended phase II dose of the combination of selinexor, carfilzomib, and dexamethasone in relapsed and relapsed/refractory MM.	For all participants, the overall response rate was 48% (3 very good partial responses and 7 partial responses), 24%, 10% and 14% of the participants achieved a minimal response, stable disease and progressive disease, respectively, and 1 participant was not evaluable. In 13 participants refractory to carfilzomib in the last line of therapy the overall response rate was 62% (2 very good partial responses and 6 partial responses) and 15% of the participants achieved a minimal response. In 17 participants refractory to proteasome inhibitor and an immunomodulatory agent/exposed to bortezomib, carfilzomib, lenalidomide and pomalidomide, the overall response rate was 53% (3 very good partial responses and 6 partial responses) and 24% of the participants achieved a minimal response. For the 5 participants enrolled at dose level 1, all refractory to carfilzomib, 80% previously treated with bortezomib and 60% refractory to the last drug, the overall response rate	[63]

					and clinical benefit rate were 60% and 80%, respectively.	

Supplementary table S1- Clinical trials of combination therapies with 20S CP inhibitors, involving patients with relapsed and/or refractory disease, and who were previously treated with a 20S CP inhibitor. Source: ClinicalTrials (https://clinicaltrials.gov/).

Study (code)	Title	20S CP inhibitor	Other drugs	Purpose	Observations	Ref.
NCT02199665 (continuation)	A Phase I Study of the Combination of a Selective Inhibitor of Nuclear Export (SINE), Selinexor with Carfilzomib and Dexamethasone in Patients with Relapsed or Relapsed/Refractory Multiple Myeloma	Carfilzomib	Dexamethasone and selinexor (a selective inhibitor of nuclear export XPO1/CRM1)	To evaluate the efficacy, the safety and the tolerability and to determine the maximum tolerated dose and the recommended phase II dose of the combination of selinexor, carfilzomib, and dexamethasone in relapsed and relapsed/refractory MM.	For the 3 participants enrolled at dose level 2a, all refractory to carfilzomib and previously treated with bortezomib, and 33% refractory to bortezomib, the overall response rate and clinical benefit rate were 67% and 100%, respectively. For the 13 participants enrolled at recommended phase 2 dose, 92% refractory to carfilzomib, all previously treated with bortezomib, and 7% refractory to bortezomib, the overall response rate and clinical benefit rate were 38% and 62%, respectively. For all response-evaluable participants, the median duration of participants who achieved minimal response or better and partial response or better, were 2.9 and 3.4 months, respectively; 3.1 and 3.0 for the recommended phase 2 dose cohort, and 2.8 and 3.3 months for the carfilzomib-refractory participants. The median progression-free survival and overall survival were 3.7 and 22.4 months, respectively, for all participants and the carfilzomib-refractory cohort.	[63]

Supplementary table S1- Clinical trials of combination therapies with 20S CP inhibitors, involving patients with relapsed and/or refractory disease, and who were previously treated with a 20S CP inhibitor. Source: ClinicalTrials (https://clinicaltrials.gov/).

Study (code)	Title	20S CP inhibitor	Other drugs	Purpose	Observations	Ref.
NCT02199665 (continuation)	A Phase I Study of the Combination of a Selective Inhibitor of Nuclear Export (SINE), Selinexor with Carfilzomib and Dexamethasone in Patients with Relapsed or Relapsed/Refractory Multiple Myeloma	Carfilzomib	Dexamethasone and selinexor (a selective inhibitor of nuclear export XPO1/CRM1)	To evaluate the efficacy, the safety and the tolerability and to determine the maximum tolerated dose and the recommended phase II dose of the combination of selinexor, carfilzomib, and dexamethasone in relapsed and relapsed/refractory MM.	The researchers concluded that selinexor could be a possible subsequent therapy, mainly as a therapy to at least transiently overcome resistance, restore disease control and prepare patients for subsequent therapies, however, they did not forgo the need of more studies to better understand the application of this regimen.	[63]
NCT02235740	An Open Label, Two-part, Phase I/Randomized Phase II Study in Subjects with Relapsed/Refractory Multiple Myeloma to Determine a Dose of Afuresertib for Administration in Combination with Carfilzomib (Part 1) and to Investigate the Safety, Pharmacokinetics, and Clinical Activity of the Combination of Afuresertib with Carfilzomib Compared with Carfilzomib Alone (Part 2)	Carfilzomib	Afuresertib (AKT inhibitor)	To determine an optimal dose and to evaluate the safety, tolerability, pharmacokinetics, and clinical activity of afuresertib in combination with carfilzomib versus carfilzomib alone, in subjects with relapsed/refractory MM, who received at least 2 prior therapies including bortezomib (but excluding carfilzomib) and an immunomodulatory agent and demonstrated disease progression on or within	Study terminated.	

				60 days of completion of the last therapy.		

Supplementary table S1- Clinical trials of combination therapies with 20S CP inhibitors, involving patients with relapsed and/or refractory disease, and who were previously treated with a 20S CP inhibitor. Source: ClinicalTrials (https://clinicaltrials.gov/).

Study (code)	Title	20S CP inhibitor	Other drugs	Purpose	Observations	Ref.
NCT02269085	A Phase I/II Trial of Ibrutinib (BTK Inhibitor) in Combination with Carfilzomib in Relapse/Refractory Mantle Cell Lymphoma	Carfilzomib	Ibrutinib	To evaluate the efficacy and to determine the maximum tolerated dose of carfilzomib combined with ibrutinib in patients with patients with relapsed or refractory MCL and who received at least 2 prior lines of therapy (prior carfilzomib, ibrutinib, bortezomib, anthracycline, rituximab or stem cell transplant are acceptable).	This study has been terminated due to the slow recruitment rate of the trial.	

Supplementary table S1- Clinical trials of combination therapies with 20S CP inhibitors, involving patients with relapsed and/or refractory disease, and who were previously treated with a 20S CP inhibitor. Source: ClinicalTrials (https://clinicaltrials.gov/).

Study (code)	Title	20S CP inhibitor	Other drugs	Purpose	Observations	Ref.
NCT02332850	A Phase Ib Study of SAR650984 (Anti-CD38 mAb) in Combination with Carfilzomib for the Treatment of Relapsed or Refractory Multiple Myeloma	Carfilzomib	Isatuximab (anti-CD38 monoclonal antibody)	To determine the maximum tolerated dose of isatuximab with standard dose carfilzomib in relapsed and refractory MM, who must have had 2 prior regimens/lines of therapy with an imunomudulator and proteasome inhibitor (including carfilzomib). Furthermore, to characterize the pharmacokinetics, safety (including immunogenicity) and disease response.	This study is currently recruiting participants.<br>In 2021, results were reported including 15 patients treated in dose escalation and 18 in the expansion cohort.<br>All patients were evaluable for response and the overall response rate was 70% (3 stringent complete responses, 8 very good partial responses and 11 partial responses) and the clinical benefit rate was 85% (additional 5 minimal responses). The median progression-free survival was 10.1 months.<br>For the subgroups of patients refractory to proteasome inhibitors, the responses were consistent. The overall response rate was 72% for the patients refractory to any proteasome inhibitor, 62% for the patients with prior carfilzomib exposure and 60% for the carfilzomib-refractory patients.<br>This study supports the combination of a CD38 antibody with carfilzomib in lenalidomide-, proteasome inhibitors- and/or dual-refractory patients.	[64]

Supplementary table S1- Clinical trials of combination therapies with 20S CP inhibitors, involving patients with relapsed and/or refractory disease, and who were previously treated with a 20S CP inhibitor. Source: ClinicalTrials (https://clinicaltrials.gov/).

Study (code)	Title	20S CP inhibitor	Other drugs	Purpose	Observations	Ref.
NCT02343042	A Phase 1b/2 Study of Selinexor (KPT-330) in Combination with Backbone Treatments for Relapsed/Refractory Multiple Myeloma	Bortezomib or carfilzomib	Selinexor and dexamethasone	The purposes are to determine the maximum tolerated dose and recommended phase II dose, and to evaluate the efficacy and safety of 6 combination therapies, in participants with relapsed/refractory MM after at least one previous line of therapy, but who were not refractory to bortezomib in their most recent line of therapy. The combinations to be evaluated include:<br>-selinexor, bortezomib and dexamethasone;<br>-selinexor, pomalidomide, dexamethasone and bortezomib;<br>-selinexor, carfilzomib and dexamethasone;<br>-selinexor, pomalidomide and dexamethasone;<br>-selinexor, lenalidomide and dexamethasone;<br>-selinexor, daratumumab and dexamethasone.	This study is currently recruiting participants.<br>Results reported in 2018 support preclinical findings that selinexor re-sensitizes and overcomes resistance to proteasome inhibitors. Between 14th October 2015 and 28th February 2017, 42 participants (38 previously treated with proteasome inhibitor therapy and 21 refractory to a proteasome inhibitor) had been treated with selinexor (100 mg orally) plus bortezomib (1.3 mg/m^2 subcutaneously) and dexamethasone (20 mg). Forty participants were evaluated for response. The proteasome inhibitor refractory participants had an overall response rate of 43% (1 complete response, 4 very good partial responses and 4 partial responses) and a clinical benefit rate of 67% (5 additional minor responses), 6 participants had stable disease and 1 had progressive disease. The median progression-free survival was 6.1 months for this subgroup.	[65]

Supplementary table S1- Clinical trials of combination therapies with 20S CP inhibitors, involving patients with relapsed and/or refractory disease, and who were previously treated with a 20S CP inhibitor. Source: ClinicalTrials (https://clinicaltrials.gov/).

Study (code)	Title	20S CP inhibitor	Other drugs	Purpose	Observations	Ref.
NCT02343042 (continuation)	A Phase 1b/2 Study of Selinexor (KPT-330) in Combination with Backbone Treatments for Relapsed/Refractory Multiple Myeloma	Bortezomib or carfilzomib	Selinexor and dexamethasone	The purposes are to determine the maximum tolerated dose and recommended phase II dose, and to evaluate the efficacy and safety of 6 combination therapies, in participants with relapsed/refractory MM after at least one previous line of therapy, but who were not refractory to bortezomib in their most recent line of therapy. The combinations to be evaluated include:<br>-selinexor, bortezomib and dexamethasone;<br>-selinexor, pomalidomide, dexamethasone and bortezomib;<br>-selinexor, carfilzomib and dexamethasone;<br>-selinexor, pomalidomide and dexamethasone;<br>-selinexor, lenalidomide and dexamethasone;<br>-selinexor, daratumumab and dexamethasone.	The 19 participants relapsed or naïve to proteasome inhibitor had an overall response rate of 84% (2 complete responses, 5 very good partial responses and 9 partial responses) and a clinical benefit rate of 95% (2 additional minor responses) and 1 of the participants had stable disease. The median progression-free survival was 17.8 months for this subgroup.	[65]

Supplementary table S1- Clinical trials of combination therapies with 20S CP inhibitors, involving patients with relapsed and/or refractory disease, and who were previously treated with a 20S CP inhibitor. Source: ClinicalTrials (https://clinicaltrials.gov/).

Study (code)	Title	20S CP inhibitor	Other drugs	Purpose	Observations	Ref.
NCT02412878	A Randomized, Open-label, Phase 3 Study in Subjects with Relapsed and Refractory Multiple Myeloma Receiving Carfilzomib in Combination with Dexamethasone, Comparing Once-weekly Versus Twice-weekly Carfilzomib Dosing	Carfilzomib	Dexamethasone	To compare once-weekly carfilzomib dosing in combination with dexamethasone to twice-weekly carfilzomib dosing in combination with dexamethasone in subjects with relapsed and refractory MM, previously treated with bortezomib and an immunomodulatory agent.	Study completed. The once weekly group received 20 mg/m^2 of carfilzomib in first day of the first cycle and 70 mg/m^2 thereafter. The twice weekly group received 20 mg/m^2 of carfilzomib in the first and second days of the first cycle and 27 mg/m^2 thereafter.<br>Results from the pre-planned interim analysis included 478 patients who were evaluated for response: 240 were randomized for the once weekly group (98% previously treated with bortezomib and 46% were refractory to any previous bortezomib) and 238 were randomized for the twice weekly group (except 1 patient who was not previously treated with bortezomib and 38% who were refractory to any previous bortezomib). In the first group the overall response was 62.9% and the median duration of overall response was 15.0 months. For the second group were 40.8% and 13.8 months, respectively.	[66–68]

Supplementary table S1- Clinical trials of combination therapies with 20S CP inhibitors, involving patients with relapsed and/or refractory disease, and who were previously treated with a 20S CP inhibitor. Source: ClinicalTrials (https://clinicaltrials.gov/).

Study (code)	Title	20S CP inhibitor	Other drugs	Purpose	Observations	Ref.
NCT02412878 (continuation)	A Randomized, Open-label, Phase 3 Study in Subjects with Relapsed and Refractory Multiple Myeloma Receiving Carfilzomib in Combination with Dexamethasone, Comparing Once-weekly Versus Twice-weekly Carfilzomib Dosing	Carfilzomib	Dexamethasone	To compare once-weekly carfilzomib dosing in combination with dexamethasone to twice-weekly carfilzomib dosing in combination with dexamethasone in subjects with relapsed and refractory MM, previously treated with bortezomib and an immunomodulatory agent.	Overall, in comparison with twice weekly carfilzomib treatment, once weekly carfilzomib treatment showed a favorable benefit–risk profile with a more convenient dosing regimen for the treatment of patients with relapsed and refractory MM. Results from a subgroup of Asian participants were published in 2019, including 45 participants who were evaluated for response all previously treated with bortezomib: 30 were for the once weekly group (40% were refractory to any previous bortezomib) and 15 for the twice weekly group (40% were refractory to any previous bortezomib). In the first group the overall response was 76.7%, the median duration of overall response was 16.0 months and the median for progression-free survival was 14.9 months. For the second group the results were 53.3%, 8.4 months and 14.8 months, respectively. Overall, in comparison with twice weekly carfilzomib treatment, once weekly carfilzomib treatment showed a comparable safety profile.	[66–68]

Supplementary table S1- Clinical trials of combination therapies with 20S CP inhibitors, involving patients with relapsed and/or refractory disease, and who were previously treated with a 20S CP inhibitor. Source: ClinicalTrials (https://clinicaltrials.gov/).

Study (code)	Title	20S CP inhibitor	Other drugs	Purpose	Observations	Ref.
NCT02412878 (continuation)	A Randomized, Open-label, Phase 3 Study in Subjects with Relapsed and Refractory Multiple Myeloma Receiving Carfilzomib in Combination with Dexamethasone, Comparing Once-weekly Versus Twice-weekly Carfilzomib Dosing	Carfilzomib	Dexamethasone	To compare once-weekly carfilzomib dosing in combination with dexamethasone to twice-weekly carfilzomib dosing in combination with dexamethasone in subjects with relapsed and refractory MM, previously treated with bortezomib and an immunomodulatory agent.	In both groups, this regimen demonstrated favorable benefit–risk profile for the treatment of Asian patients with relapsed and refractory MM.	[66–68]
NCT02461888	A Randomized Phase II Trial of Cyclophosphamide and Dexamethasone in Combination with Ixazomib in Relapsed or Refractory Multiple Myeloma (RRMM) Patients Who Have Relapsed After Treatment with Thalidomide, Lenalidomide and Bortezomib	Ixazomib	Cyclophosphamide and dexamethasone	To evaluate the efficacy, the safety and toxicity of the combination of ixazomib, cyclophosphamide and dexamethasone, in patients with relapsed or refractory MM and who have relapsed after treatment with thalidomide, lenalidomide and bortezomib.	According to ClinicalTrials.gov, the recruitment status of this study is unknown.<br>The participants will either receive ixazomib with cyclophosphamide and dexamethasone or cyclophosphamide and dexamethasone alone.	

Supplementary table S1- Clinical trials of combination therapies with 20S CP inhibitors, involving patients with relapsed and/or refractory disease, and who were previously treated with a 20S CP inhibitor. Source: ClinicalTrials (https://clinicaltrials.gov/).

Study (code)	Title	20S CP inhibitor	Other drugs	Purpose	Observations	Ref.
NCT02477215	Phase I/II Study of Bendamustine and IXAZOMIB (MLN9708) Plus Dexamethasone in Relapsed/Refractory Multiple Myeloma	Ixazomib	Bendamustine and dexamethasone	In phase I, to analyze 3 doses of bendamustine 70, 80 and 90 mg/m^2 combined with fixed-doses of ixazomib 4 mg and dexamethasone 40 mg (20 mg in patients ≥75 years), when delivered together in 28-day cycles; to determine the recommended phase II dose and to assess the efficacy of these drugs combined in patients with relapsed/refractory MM, and who progressed after prior exposure to proteasome inhibitor (bortezomib, carfilzomib) and immunomodulatory agent (lenalidomide or pomalidomide or thalidomide); and are refractory/progressing to at least one of these agents. In phase II, to estimate the efficacy of this regimen.	Study completed. The participants could not be previously exposed to ixazomib. Between October 2015 and January 2018, 28 participants were enrolled: 15 in phase I and 13 in phase II. The participants received a median of 4 prior lines of therapy, in all cases including bortezomib and in 43% of the participants carfilzomib. In any prior line of therapy, 64% were bortezomib-refractory participants and 11% carfilzomib-refractory. Twenty-six participants (phase I and II) were evaluable for response and the overall response rate was 48%, the median of duration of response, progression-free survival and overall survival were 5.5, 5.2 and 23.2 months, respectively. Eighteen participants were treated in phase II dosing scheme and the overall response rate was 61%. For all dose levels, 12 participants were exposed but not refractory to proteasome inhibitors, and for these participants the median of overall response rate was 83% (2very good partial responses and 8 partial responses) and 16.5% of participants had progressive disease.	[69]

Supplementary table S1- Clinical trials of combination therapies with 20S CP inhibitors, involving patients with relapsed and/or refractory disease, and who were previously treated with a 20S CP inhibitor. Source: ClinicalTrials (https://clinicaltrials.gov/).

Study (code)	Title	20S CP inhibitor	Other drugs	Purpose	Observations	Ref.
NCT02477215 (continuation)	Phase I/II Study of Bendamustine and IXAZOMIB (MLN9708) Plus Dexamethasone in Relapsed/Refractory Multiple Myeloma	Ixazomib	Bendamustine and dexamethasone	In phase I, to analyze 3 doses of bendamustine 70, 80 and 90 mg/m^2 combined withfixed-doses of ixazomib 4 mg and dexamethasone 40 mg (20 mg in patients ≥75 years), when delivered together in 28-day cycles; to determine the recommended phase II dose and to assess the efficacy of these drugs combined in patients with relapsed/refractory MM, and who progressed after prior exposure to proteasome inhibitor (bortezomib, carfilzomib) and immunomodulatory agent (lenalidomide or pomalidomide or thalidomide); and are refractory/progressing to at least one of these agents. In phase II, to estimate the efficacy of this regimen.	For all dose levels, 3 participants were only refractory to bortezomib, and in this subgroup 1 participant achieved a partial response and 2 had stable disease. For all dose levels, 13 participants evaluated were refractory to bortezomib and carfilzomib, and in this subgroup 1 participant achieved a partial response, 8 had stable disease, 3 had progressive disease. The researchers observed that the overall response rate in their study was comparable to bortezomib-bendamustine-dexamethasone in relapsed/refractory MM patients (60.8%), including patients previously exposed to bortezomib, but not refractory to this proteasome inhibitor.	[69]

Supplementary table S1- Clinical trials of combination therapies with 20S CP inhibitors, involving patients with relapsed and/or refractory disease, and who were previously treated with a 20S CP inhibitor. Source: ClinicalTrials (https://clinicaltrials.gov/).

Study (code)	Title	20S CP inhibitor	Other drugs	Purpose	Observations	Ref.
NCT02542657	Phase I/II Study of Ixazomib in Combination with Pomalidomide, Clarithromycin and Dexamethasone (PiC-D) in Patients with Double Refractory Multiple Myeloma	Ixazomib	Clarithromycin (macrolide antibiotic, but that has anti-tumor activity), dexamethasone and pomalidomide	The purposes of phase I were to establish the maximum tolerated dose and the recommended phase II dose and to evaluate the safety and tolerability, to assess preliminary evidence of clinical activity and characterize the pharmacokinetics of ixazomib combined with pomalidomide, clarithromycin and dexamethasone, in patients with MM who are bortezomib and lenalidomide refractory. The purpose of phase II was to evaluate the safety and the efficacy of this regimen.	This study is ongoing, but not recruiting participants. The participants could not be previously exposed to ixazomib or pomalidomide.	

Supplementary table S1- Clinical trials of combination therapies with 20S CP inhibitors, involving patients with relapsed and/or refractory disease, and who were previously treated with a 20S CP inhibitor. Source: ClinicalTrials (https://clinicaltrials.gov/).

Study (code)	Title	20S CP inhibitor	Other drugs	Purpose	Observations	Ref.
NCT02568943	An Open-label, Multi-center, Expanded Treatment Protocol of Oral Panobinostat in Combination with Bortezomib and Dexamethasone in Patients with Relapsed, and Relapsed and Refractory Multiple Myeloma	Bortezomib	Dexamethasone and panobinostat	To acquire additional safety data on the use of panobinostat in combination with bortezomib and dexamethasone in patients with relapsed or relapsed and refractory MM and who require retreatment.	Expanded access was available for this intervention previously, however it is not currently available and will not be available in the future.<br>Patients previously treated with bortezomib would be eligible for the study, even if they are deemed refractory.	
NCT02628704	Phase 2, Randomized, Double-blind, Placebo-controlled, Multicenter Study of Selinexor (KPT-330), Carfilzomib, and Dexamethasone in Patients with Relapsed/Refractory Multiple Myeloma Previously Treated with a Proteasome Inhibitor and an Immunomodulatory Drug	Carfilzomib	Dexamethasone and selinexor	To compare the efficacy and assess the safety of selinexor plus carfilzomib plus low-dose dexamethasone versus placebo plus carfilzomib plus low-dose dexamethasone in patients with relapsed/refractory MM, who have received at least 2 prior therapies, including a proteasome inhibitor and an immunomodulatory agent.	This study has been withdrawn prior to enrollment.	

Supplementary table S1- Clinical trials of combination therapies with 20S CP inhibitors, involving patients with relapsed and/or refractory disease, and who were previously treated with a 20S CP inhibitor. Source: ClinicalTrials (https://clinicaltrials.gov/).

Study (code)	Title	20S CP inhibitor	Other drugs	Purpose	Observations	Ref.
NCT02718833	A Phase II Study of Elotuzumab in Combination with Pomalidomide, Bortezomib, and Dexamethasone in Relapsed and Refractory Multiple Myeloma	Bortezomib	Elotuzumab (a monoclonal antibody that targets the protein SLAMF7 which is highly common on the surface of MM cells), pomalidomide and dexamethasone	To evaluate the safety and efficacy of the combination of elotuzumab, pomalidomide, bortezomib, dexamethasone, in patients with relapsed and refractory MM.	This study is ongoing, but not recruiting participants.<br>Patients must have received at least 1 prior therapy with at least 2 cycles of lenalidomide and at least 2 cycles of a proteasome inhibitor (either in separate regimens or within the same regimen).<br>Results reported in 2018 included 33 participants. All participants had prior exposure to lenalidomide and proteasome inhibitors (94% to bortezomib and 76% to carfilzomib) and were refractory to their last line of therapy. Thirty-one participants were assessable for response and the best overall response rate was 52% (1 complete response, 4 very good partial responses and 11 partial responses) and the median progression-free survival was 9.7 months. The overall response rate was 43% for the participants previously treated with carfilzomib.	[70]

Supplementary table S1- Clinical trials of combination therapies with 20S CP inhibitors, involving patients with relapsed and/or refractory disease, and who were previously treated with a 20S CP inhibitor. Source: ClinicalTrials (https://clinicaltrials.gov/).

Study (code)	Title	20S CP inhibitor	Other drugs	Purpose	Observations	Ref.
NCT02831686	An Investigator-Initiated Phase I Study of Selinexor (KPT-330), Ixazomib, and Low Dose Dexamethasone in Patients with Relapsed and/or Refractory Multiple Myeloma	Ixazomib	Dexamethasone and selinexor	To evaluate the safety and to determine the maximum tolerated dose of selinexor combined with ixazomib and with a low dose of dexamethasone in patients with relapsed and/or refractory MM after therapy with at least one immunomodulatory drug and at least one proteasome inhibitor.	Study completed.	
NCT02939183	(INTREPID-1) A Phase 1b Study Evaluating the Safety, Tolerability, Pharmacokinetics and Efficacy of Oprozomib in Combination with Pomalidomide and Dexamethasone in Subjects with Relapsed or Refractory Multiple Myeloma	Oprozomib	Dexamethasone and pomalidomide	To evaluate 2 new formulations of oprozomib plus dexamethasone or plus dexamethasone and pomalidomide in patients with relapsed or refractory MM after at least 2 lines of therapy and with prior therapeutic treatment or regimens which must include a proteasome inhibitor and lenalidomide.	This study is ongoing, but not recruiting participants.	

Supplementary table S1- Clinical trials of combination therapies with 20S CP inhibitors, involving patients with relapsed and/or refractory disease, and who were previously treated with a 20S CP inhibitor. Source: ClinicalTrials (https://clinicaltrials.gov/).

Study (code)	Title	20S CP inhibitor	Other drugs	Purpose	Observations	Ref.
NCT03029234	An Open-label, Single-arm, Phase 3 Study of Carfilzomib in Combination with Dexamethasone in Subjects with Relapsed and Refractory Multiple Myeloma in China	Carfilzomib	Dexamethasone	To evaluate the safety, tolerability and efficacy of carfilzomib in combination with dexamethasone for the treatment of relapsed and refractory MM in China.	Study completed. Participants must have received prior treatment with bortezomib and an immunomodulatory agent and an alkylating agent or anthracycline alone or in combination with other treatments for MM. Participants could not have been previously treated with carfilzomib.<br>In this study, 126 participants were enrolled: 75.6% refractory to a proteasome inhibitor and 74% refractory to a proteasome inhibitor and an immunomodulatory drug. The authors concluded that carfilzomib combined with dexamethasone led to a clinically meaningful benefit as an additional treatment for heavily pretreated patients with relapsed and refractory MM in China.	[71]
NCT03104270	A Phase 2 Trial of the Efficacy and Safety of Elotuzumab in Combination with Pomalidomide, Carfilzomib and Dexamethasone Among High Risk Relapsed/ Refractory Multiple Myeloma Patients	Carfilzomib	Dexamethasone, elotuzumab and pomalidomide	To evaluate efficacy and safety of elotuzumab in combination with pomalidomide, carfilzomib and dexamethasone among high risk relapsed and refractory MM patients.	This study is ongoing, but not recruiting participants. The participants included had a diagnosis of high-risk MM, had previously received more than 2 lines of therapy including a lenalidomide-containing regimen and proteasome inhibitor-containing regimen and had demonstrated progressive disease while on their last regimen, but were not exposed to elotuzumab.	

Supplementary table S1- Clinical trials of combination therapies with 20S CP inhibitors, involving patients with relapsed and/or refractory disease, and who were previously treated with a 20S CP inhibitor. Source: ClinicalTrials (https://clinicaltrials.gov/).

Study (code)	Title	20S CP inhibitor	Other drugs	Purpose	Observations	Ref.
NCT03117361	Phase II Trial of Plitidepsin (Aplidin®) in Combination with Bortezomib and Dexamethasone in Multiple Myeloma Patients Double Refractory to Bortezomib and Lenalidomide	Bortezomib	Dexamethasone and plitidepsin	To evaluate the efficacy of plitidepsin in combination with bortezomib and dexamethasone in patients with MM double refractory to bortezomib and lenalidomide.	This study has been terminated due to the slow recruitment rate of the trial.	
NCT03136653	A Phase 2 Open-label, Single-arm, Multicenter Trial of MP0250 Plus Bortezomib + Dexamethasone in Patients with Refractory and Relapsed Multiple Myeloma	Bortezomib	Dexamethasone and MP0250 (a multi-DARPin, able to simultaneously neutralize the activities of vascular endothelial growth factor and hepatocyte growth factor and also to bind to human serum albumin to give an increased plasma half-life and potentially enhanced tumor penetration)	To assess the efficacy, safety, tolerability, pharmacokinetics, immunogenicity and biological activity of MP0250 in combination with bortezomib and dexamethasone in patients with refractory and relapsed MM who have received at least 2 lines of therapy (including bortezomib and an immunomodulatory agent) and have shown no response to, have progressed on the most recent treatment or have progressed within 60 days of the most recent therapy.	A phase I/II study completed.	

Supplementary table S1- Clinical trials of combination therapies with 20S CP inhibitors, involving patients with relapsed and/or refractory disease, and who were previously treated with a 20S CP inhibitor. Source: ClinicalTrials (https://clinicaltrials.gov/).

Study (code)	Title	20S CP inhibitor	Other drugs	Purpose	Observations	Ref.
NCT03155100	Phase 2 Study of Carfilzomib + Elotuzumab + Dexamethasone for Relapsed or Progressed Multiple Myeloma After 1-3 Prior Treatment Lines	Carfilzomib	Dexamethasone and elotuzumab	To assess the safety and the efficacy of carfilzomib combined with elotuzumab and dexamethasone, in patients with relapsed or progressed MM, after 1 to 3 prior treatment lines in which proteasome inhibitors (bortezomib, carfilzomib and/or ixazomib) and/or lenalidomide have been included.	This study is ongoing, but not recruiting participants. Participants refractory to bortezomib, ixazomib and/or lenalidomide were allowed, but participants refractory to carfilzomib were excluded.	

Supplementary table S1- Clinical trials of combination therapies with 20S CP inhibitors, involving patients with relapsed and/or refractory disease, and who were previously treated with a 20S CP inhibitor. Source: ClinicalTrials (https://clinicaltrials.gov/).

Study (code)	Title	20S CP inhibitor	Other drugs	Purpose	Observations	Ref.
NCT03158688	A Randomized, Open-label, Phase 3 Study Comparing Carfilzomib, Dexamethasone, and Daratumumab to Carfilzomib and Dexamethasone for the Treatment of Patients with Relapsed or Refractory Multiple Myeloma	Carfilzomib	Daratumumab and dexamethasone	To evaluate the efficacy and the global health status/ quality of carfilzomib in combination only with dexamethasone or with daratumumab and dexamethasone, in of patients with relapsed or refractory MM.	This study is ongoing, but not recruiting participants. Results reported in 2020 and 2021 included 466 participants: 312 participants treated with the triple regimen and 154 participants treated with the double regimen. The daratumumab-containing regimen significantly prolonged progression-free survival and reduced the risk of progression or death in comparison with the regimen without daratumumab. Most participants of this study (90%) were previously treated with bortezomib. The daratumumab-containing regimen significantly reduced the risk of progression or death by 36% for participants with previous proteasome inhibitor exposure. The favorable benefit-risk profile of the daratumumab-containing regimen is consistent for the participants with previous exposure or resistance to bortezomib or ixazomib.	[52,72–75]

Supplementary table S1- Clinical trials of combination therapies with 20S CP inhibitors, involving patients with relapsed and/or refractory disease, and who were previously treated with a 20S CP inhibitor. Source: ClinicalTrials (https://clinicaltrials.gov/).

Study (code)	Title	20S CP inhibitor	Other drugs	Purpose	Observations	Ref.
NCT03201250	A Phase I/II Study of the c-Met Inhibitor Cabozantinib as a Targeted Strategy to Reverse Resistance to the Proteasome Inhibitor Carfilzomib in Refractory Multiple Myeloma	Carfilzomib	Cabozantinib (inhibitor of the proto-oncogene c-MET) and dexamethasone	To evaluate the cabozantinib as a targeted strategy to reverse resistence to the carfilzomib in patients with refractory MM. To evaluate the efficacy, the safety, the tolerance and the toxicity and to determine the maximum tolerated dose of cabozantinib combined with carfilzomib and dexamethasone.	This study is currently recruiting participants. The participants must have failed carfilzomib either as a single agent as the last form of therapy, or carfilzomib in combination with dexamethasone, carfilzomib in combination with lenalidomide and dexamethasone, carfilzomib in combination with pomalidomide and dexamethasone or carfilzomib in combination with cyclophosphamide and dexamethasone.	
NCT03269552	A Response-Adapted Clinical Trial of Weekly Carfilzomib with or without Rituximab for Waldenström's Macroglobulinemia and Marginal Zone Lymphoma	Carfilzomib	Rituximab	To evaluate the efficacy, the safety and tolerability of carfilzomib with or without rituximab in treating patients with Waldenstrom macroglobulinemia or marginal zone lymphoma that is previously untreated or with relapsed/refractory disease.	A phase II that has been terminated (slow accrual). Participants who are not responding to the treatment of carfilzomib alone (after 2 courses), would also receive rituximab in the treatment.	

Supplementary table S1- Clinical trials of combination therapies with 20S CP inhibitors, involving patients with relapsed and/or refractory disease, and who were previously treated with a 20S CP inhibitor. Source: ClinicalTrials (https://clinicaltrials.gov/).

Study (code)	Title	20S CP inhibitor	Other drugs	Purpose	Observations	Ref.
NCT03323151	A Phase I/II Study of Ixazomib and Ibrutinib in Relapsed/Refractory Mantle Cell Lymphoma	Ixazomib	Ibrutinib	The purpose of phase I is to determine the maximum safe and tolerated dose/recommended phase II dose of ixazomib combined with ibrutinib in patients with relapsed or refractory MCL. The purpose of phase II is to evaluate the safety and the efficacy of this combination. In Phase II, patients will be separated into 2 groups, patients who have never received ibrutinib and patients who have received ibrutinib.	This study is ongoing, but not recruiting participants. Prior proteasome inhibitor and/or Bruton's tyrosine kinase inhibitors are allowed, but participants may not have been exposed to the combination of proteasome inhibitor and Bruton's tyrosine kinase inhibitor.	

Supplementary table S1- Clinical trials of combination therapies with 20S CP inhibitors, involving patients with relapsed and/or refractory disease, and who were previously treated with a 20S CP inhibitor. Source: ClinicalTrials (https://clinicaltrials.gov/).

Study (code)	Title	20S CP inhibitor	Other drugs	Purpose	Observations	Ref.
NCT03361306	LCI-HEM-MYE-CRD-002: A Phase II Study of Carfilzomib- Revlimid-Dexamethasone-Elotuzumab in Relapsed/Refractory Multiple Myeloma	Carfilzomib	Dexamethasone, elotuzumab and lenalidomide	To evaluate the efficacy of induction therapy comprised of 4 cycles of carfilzomib, lenalidomide, dexamethasone and elotuzumab in patients with relapsed and/or refractory MM.	This study is ongoing, but not recruiting participants. Patients refractory to bortezomib and/or lenalidomide are eligible. Patients who previously received carfilzomib are eligible provided they experienced a minimal response or better and relapsed more 60 days after completion of treatment. Post induction, all subjects will undergo disease evaluation for assessment of the primary endpoint. Maintenance therapy comprised of elotuzumab and lenalidomide will start directly after induction and continue until relapse or progression.	[76]
NCT03399539	Phase I/II Clinical Trial of Venetoclax (ABT-199) in Combination with Ixazomib and Dexamethasone for Patients with Relapsed Multiple Myeloma	Ixazomib	Dexamethasone and venetoclax	To determine the maximum tolerated dose of venetoclax in combination with ixazomib and dexamethasone in patients with relapsed MM. To evaluate the toxicity and efficacy of this combination.	This study is ongoing, but not recruiting participants. The patients that will be enrolled should have received a proteasome inhibitor (exception to ixazomib treatment) and an immunomodulatory drug.	

Supplementary table S1- Clinical trials of combination therapies with 20S CP inhibitors, involving patients with relapsed and/or refractory disease, and who were previously treated with a 20S CP inhibitor. Source: ClinicalTrials (https://clinicaltrials.gov/).

Study (code)	Title	20S CP inhibitor	Other drugs	Purpose	Observations	Ref.
NCT03492138	A Phase I/II Study of the Addition of Ixazomib to ONC201 and Dexamethasone in Relapsed and/or Refractory Multiple Myeloma	Ixazomib	Dexamethasone and ONC201 (dopamine receptor D2 antagonist that is able to activate the integrated stress response pathway and it is active against MM cells)	The purpose of phase I was to determine the recommended phase II dose of ixazomib in combination with ONC201 and dexamethasone. The purpose of phase II was to evaluate the efficacy of this combinatory therapy in patients with relapsed/refractory MM.	This study has been terminated due to the slow recruitment rate of the trial. In order to document superiority over the combination compared to the individual agents of ixazomib and ONC201 in a single arm study, initially, the participants would be treated in a run-in phase of the study with dexamethasone and ONC201 such that if there is a progression of disease after 4 weeks or less than a minimal response after 8 weeks then ixazomib would be added. If patients achieved single-agent responses with ONC201 (minimal response or better), they would continue with weekly ONC201 and dexamethasone until progression, with response assessments after each 28-day cycle. Patients who have previously been treated on another clinical trial with weekly ONC201 (625mg) with dexamethasone with progression while receiving treatment would not need to complete the run-in phase of the study. The target population included patients with symptomatic MM having progressed on 2 prior therapies including a proteasome inhibitor (including ixazomib). Proteasome inhibitor refractory patients were eligible.	

Supplementary table S1- Clinical trials of combination therapies with 20S CP inhibitors, involving patients with relapsed and/or refractory disease, and who were previously treated with a 20S CP inhibitor. Source: ClinicalTrials (https://clinicaltrials.gov/).

Study (code)	Title	20S CP inhibitor	Other drugs	Purpose	Observations	Ref.
NCT03457142	Phase II Study of Targeting CD28 in Multiple Myeloma with Abatacept (CTLA4-Ig) to Overcome Resistance to Chemotherapy	Ixazomib	Abatacept (a selective T cell co-stimulation modulator) and dexamethasone	To evaluate the efficacy and toxicity of abatacept in combination with ixazomib and dexamethasone in MM patients in first relapse (or who are primary refractory) following treatment with a bortezomib-containing regimen, compared to historical controls of ixazomib in combination with dexamethasone.	This study is currently recruiting participants. The prior treatment with ixazomib is one of the exclusion criteria's for the enrollment of the participants.	
NCT03506360	Phase 2 Trial of Pembrolizumab, Ixazomib, and Dexamethasone for Relapsed Multiple Myeloma	Ixazomib	Dexamethasone and pembrolizumab (a humanized monoclonal antibody, which binds to the programmed cell death-1, PD-1, receptor and blocks its interaction with the ligands PD-L1 and PD-L2)	To evaluate the efficacy and toxicity of pembrolizumab in combination with ixazomib and dexamethasone in patients with relapsed symptomatic MM	This study is ongoing, but not recruiting participants. The participants must have relapsed or refractory disease after treatments including 3 therapies: proteasome inhibitors (exception to ixazomib treatment), immunomodulatory imide drugs and anti-CD38 antibody.	

Supplementary table S1- Clinical trials of combination therapies with 20S CP inhibitors, involving patients with relapsed and/or refractory disease, and who were previously treated with a 20S CP inhibitor. Source: ClinicalTrials (https://clinicaltrials.gov/).

Study (code)	Title	20S CP inhibitor	Other drugs	Purpose	Observations	Ref.
NCT03605719	PD1 Blockade and Oncolytic Virus in Relapsed Multiple Myeloma	Carfilzomib	Dexamethasone, nivolumab (a human IgG4 monoclonal antibody, which binds to the programmed cell death-1, PD-1, receptor and blocks its interaction with the ligands PD-L1 and PD-L2), pelareorep (wild-type reovirus) and pomalidomide	To evaluate the safety, side effects and best dose of pelareorep when given together with dexamethasone, carfilzomib, and nivolumab with or without pomalidomide in treating participants with relapsed/refractory MM.	This phase I study is currently recruiting participants. Participants are assigned to 1 of 3 arms: dexamethasone, carfilzomib and nivolumab (arm I); dexamethasone, pelareorep, carfilzomib and nivolumab (arm II) or dexamethasone, pelareorep, carfilzomib, nivolumab and pomalidomide (arm III). In arm I patients who must be carfilzomib naïve will be enrolled. In arm II patients who must have evidence of proteasome resistance will be enrolled. In arm III patients who have evidence of proteasome inhibitor moderate resistance and pomalidomide exposition will be enrolled.	
NCT03856112	A Phase 1/2 Study of MLN9708 (Ixazomib [I]), Venetoclax (V), and Dexamethasone (D) Regimen (IVD) to Restore or Enhance Proteasome Inhibitor (PI) Sensitivity in Non-t(11;14) Relapsed/Refractory Multiple Myeloma (RRMM)	Ixazomib	Venetoclax and dexamethasone	To evaluate the safety profile, tolerability and efficacy of combination therapy with ixazomib, venetoclax and dexamethasone in treating patients with non-t(11;14) relapsed/refractory MM	withdrawn (Per CTEP, Martha Khrum this study is withdrawn. Changing status to update CT.gov). Overall response rate of this combinatory treatment would be evaluated in proteasome inhibitor-refractory cohort (excluding previous treatment with ixazomib).	

Supplementary table S1- Clinical trials of combination therapies with 20S CP inhibitors, involving patients with relapsed and/or refractory disease, and who were previously treated with a 20S CP inhibitor. Source: ClinicalTrials (https://clinicaltrials.gov/).

Study (code)	Title	20S CP inhibitor	Other drugs	Purpose	Observations	Ref.
NCT04065789	Safety, Tolerability, and Efficacy of Once Weekly Carfilzomib in Combination with Daratumumab, Lenalidomide and Dexamethasone, in Transplant-ineligible Multiple Myeloma Patients Non-responsive to a Bortezomib Based Induction	Carfilzomib	Daratumumab, lenalidomide and dexamethasone	To evaluate the safety, efficacy, the global health status, quality of life and frailty status of carfilzomib in combination with daratumumab, lenalidomide and dexamethasone, in transplant-ineligible MM patients non-responsive to a bortezomib based induction.	Study completed.<br>The participants were divided into 2 cohorts: cohort A for the participants with MM who received a bortezomib-based induction and either failed to achieve a minimal response until 4 months, and cohort B if have a progressed early disease (less 18 months). The overall response rate was 90% and 86%, respectively, to cohorts A and B.	[77]
NCT04094961	Phase I/II Study of Twice Weekly Ixazomib Plus Pomalidomide and Dexamethasone in Relapsed/or Refractory Multiple Myeloma	Ixazomib	Pomalidomide and dexamethasone	To define the appropriate dose of the investigational drug to use for further studies; to evaluate the safety and efficacy of the ixazomib combined with pomalidomide and dexamethasone, in patients with relapsed or relapsed-refractory MM.	This study is currently recruiting participants. One inclusion criterion is participants who had received at least two previous therapies or received one prior line of therapy if previously treated with an immunomodulatory drug plus a proteasome inhibitor and have demonstrated disease progression on or within 60 days of completion of the last therapy. Participants that have previously been treated with ixazomib or are refractory to pomalidomide will be excluded.	

Supplementary table S1- Clinical trials of combination therapies with 20S CP inhibitors, involving patients with relapsed and/or refractory disease, and who were previously treated with a 20S CP inhibitor. Source: ClinicalTrials (https://clinicaltrials.gov/).

Study (code)	Title	20S CP inhibitor	Other drugs	Purpose	Observations	Ref.
NCT04119336	A Phase II Study of Nivolumab in Combination with Ixazomib, Cyclophosphamide, and Dexamethasone in Relapsed and Refractory Multiple Myeloma	Ixazomib	Nivolumab, cyclophosphamide, and dexamethasone	To assess the effectiveness and safety of the combination of nivolumab with ixazomib, cyclophosphamide, and dexamethasone in relapsed and refractory MM.	This study is ongoing, but not recruiting participants. Patients must have received at least three prior lines of therapy, including an immunomodulatory drug, a proteasome inhibitor (excluding ixazomib), and anti-CD38 monoclonal antibody.	
NCT04163107	Combined Carfilzomib and Hydroxychloroquine in Patients with Relapsed/Refractory Multiple Myeloma - a Phase 1 Trial	Carfilzomib	Hydroxychloro-quine and dexamethasone	To determine a maximum tolerated dose of this combination and to study tolerability of the addiction of hydroxychloroquine to a standard regimen of carfilzomib and dexamethasone in patients with relapsed or progressive disease of MM.	Pre-clinical studies have shown that the combination of carfilzomib and the autophagy inhibitor hydroxychloroquine increases myeloma cell death and that hydroxychloroquine is able to reverse MM cell resistance to carfilzomib.<br>This study is ongoing, but not recruiting participants. Participants must have received at least two prior therapies including bortezomib and an immunomodulatory agent (may include autologous bone marrow transplantation), however, they must not be refractory to carfilzomib.	

Supplementary table S1- Clinical trials of combination therapies with 20S CP inhibitors, involving patients with relapsed and/or refractory disease, and who were previously treated with a 20S CP inhibitor. Source: ClinicalTrials (https://clinicaltrials.gov/).

Study (code)	Title	20S CP inhibitor	Other drugs	Purpose	Observations	Ref.
NCT04176718	A Phase II Study of Daratumumab with Weekly Carfilzomib, Pomalidomide, and Dexamethasone in Relapsed and Refractory Multiple Myeloma	Carfilzomib	Daratumumab, pomalidomide, and dexamethasone	To evaluate the toxicity and efficacy of the daratumumab, carfilzomib, pomalidomide, and dexamethasone combination in patients with relapsed and refractory MM, who have received at least one prior therapy and who have had previous treatment with both lenalidomide and a proteasome inhibitor (either in separate regimens or within the same regimen).	This study is currently recruiting participants. The participants treated in the last line of therapy with the combination of carfilzomib, pomalidomide, and dexamethasone will be excluded. Prior treatment with carfilzomib or pomalidomide is permitted (as different lines of treatment but not in the same combination).	
NCT04392648	A Phase 1 Open-label Study to Evaluate the Safety, Tolerability and Efficacy of Intravenous TAK-573 as Part of Combination Therapy in Patients with Relapsed or Refractory Multiple Myeloma	Bortezomib	TAK-573 (an anti-CD38 attenuated IFNα fusion protein) and dexamethasone	To determine the safety, tolerability, efficacy and recommended phase II dose of TAK-573 in combination with dexamethasone and bortezomib, in patients with relapsed and refractory MM, who have received at least 2 prior therapies, including treatment with lenalidomide and a proteasome inhibitor.	This study has been withdrawn prior to enrollment.	

Supplementary table S1- Clinical trials of combination therapies with 20S CP inhibitors, involving patients with relapsed and/or refractory disease, and who were previously treated with a 20S CP inhibitor. Source: ClinicalTrials (https://clinicaltrials.gov/).

Study (code)	Title	20S CP inhibitor	Other drugs	Purpose	Observations	Ref.
NCT04414475	A Phase 2b, Open-label, Multi-arm Clinical Trial of Selinexor Plus Low-dose Dexamethasone (Sd) in Patients with Penta-refractory Multiple Myeloma or Selinexor and Bortezomib Plus Low-dose Dexamethasone (SVd) in Patients with Triple-class Refractory Multiple Myeloma	Bortezomib	Selinexor and dexamethasone	To compare the efficacy, safety and tolerability with the combination regimens: selinexor plus bortezomib and low-dose dexamethasone and selinexor plus low-dose dexamethasone in patients with refractory MM, including the previous treatment with PI.	This study is currently recruiting participants.	
NCT04661137	A Phase 2B Study of Selinexor (KPT-330), in Combination with Carfilzomib, Daratumumab or Pomalidomide in Patients with Multiple Myeloma Relapsing on Current Therapy	Carfilzomib	Selinexor	To compare the efficacy and safety of selinexor plus carfilzomib in patients with MM, and refractory to or disease progression while on a carfilzomib-containing regimen.	This study is currently recruiting participants.	

Supplementary table S1- Clinical trials of combination therapies with 20S CP inhibitors, involving patients with relapsed and/or refractory disease, and who were previously treated with a 20S CP inhibitor. Source: ClinicalTrials (https://clinicaltrials.gov/).

Study (code)	Title	20S CP inhibitor	Other drugs	Purpose	Observations	Ref.
NCT04764942	Phase 1/2 Trial of Selinexor in Combination with Pomalidomide and Dexamethasone ± Carfilzomib for Patients with Proteasome-Inhibitor and Immunomodulatory Drug Refractory Multiple Myeloma (SCOPE)	Carfilzomib	Selinexor, pomalidomide and dexamethasone	To compare the efficacy, safety and tolerability with the combination regimens: selinexor plus pomalidomide and dexamethasone and selinexor plus carfilzomib, pomalidomide and dexamethasone, in patients with relapsed and/or refractory MM. To assess the overall-related quality of life of the participants.	This study is currently recruiting participants. The participants that will be included in the carfilzomib regimen must have progressive disease and be exposed to up to 2 prior lines of therapy, including a proteasome inhibitor and lenalidomide.	
NCT04790474	A Single-arm, Multisite, Prospective Study of Ixazomib-pomalidomide-dexamethasone as Second or Third-line Combination Treatment for Patients with Relapsed and Refractory Multiple Myeloma (RRMM) Previously Treated with Daratumumab, Lenalidomide and Bortezomib (IPoD-790 Study)	Ixazomib	Pomalidomide and dexamethasone	To evaluate the safety, tolerability and efficacy of ixazomib plus pomalidomide and dexamethasone as a second or third-line combination treatment for patients with relapsed and/or refractory MM who progressed after receiving bortezomib, lenalidomide and daratumumab during first and second lines	This phase II study is currently recruiting participants.	

Supplementary table S1- Clinical trials of combination therapies with 20S CP inhibitors, involving patients with relapsed and/or refractory disease, and who were previously treated with a 20S CP inhibitor. Source: ClinicalTrials (https://clinicaltrials.gov/).

Study (code)	Title	20S CP inhibitor	Other drugs	Purpose	Observations	Ref.
NCT04813653	A Single-arm, Prospective Study of Cyclosporine in Combination with Carfilzomib and Dexamethasone in Patients with Relapsed Multiple Myeloma Refractory to Carfilzomib with High Expression of the Peptidylprolyl Isomerase A (PPIA) Gene in Myeloma Cells	Carfilzomib	Cyclosporine and dexamethasone	To evaluate the safety, tolerability and efficacy of cyclosporine in combination with carfilzomib and dexamethasone in patients with relapsed and refractory MM who have received at least 2 prior lines of therapy including carfilzomib, and were non-responsive or refractory to carfilzomib, and with high expression of the Peptidylprolyl Isomerase A (PPIA) gene in myeloma cells.	This phase I study is currently recruiting participants.	
NCT04956302	A Phase I Study of Panobinostat in Combination with Daratumumab, Bortezomib, and Dexamethasone in Patients with Relapsed/Refractory Multiple Myeloma	Bortezomib	Panobinostat, daratumumab and dexamethasone	To evaluate the safety, tolerability and efficacy of bortezomib plus panobinostat, daratumumab and dexamethasone in relapsed/refractory MM of patients who have previously received one line of therapy including lenalidomide or cyclophosphamide, a proteasome inhibitor, with	This study is currently recruiting participants.	

				or without autologous stem cell transplant.		

Supplementary table S1- Clinical trials of combination therapies with 20S CP inhibitors, involving patients with relapsed and/or refractory disease, and who were previously treated with a 20S CP inhibitor. Source: ClinicalTrials (https://clinicaltrials.gov/).

Study (code)	Title	20S CP inhibitor	Other drugs	Purpose	Observations	Ref.
NCT05050305	A Phase 2 Study with Safety Run-In of Marizomib, Pomalidomide, and Dexamethasone for Relapsed and Refractory Multiple Myeloma and CNS Myeloma	Marizomib	Pomalidomide and dexamethasone	To evaluate the safety and efficacy of marizomib combined with pomalidomide and dexamethasone, in participants with relapsed and relapsed/refractory MM and central nervous system myeloma. To determine the maximum tolerated dose of this combination.	This study is currently recruiting participants. Participants must have received at least one or more previous lines of therapy including an immunomodulatory drug and a proteasome inhibitor (exception marizomib) and have demonstrated disease progression on or within 60 days of completion of the last therapy.	
NCT05060627	An Open Label, Multicenter, Phase I/II Study of Belantamab Mafodotin in Combination with Kd for the Treatment of Relapsed Myeloma Patients, Refractory to Lenalidomide	Carfilzomib	Belantamab mafodotin and dexamethasone	To evaluate the safety and efficacy of belantamab mafodotin combined with carfilzomib and dexamethasone, in participants with relapsed myeloma patients, refractory to lenalidomide. To determine the maximum tolerated dose of this combination.	This study is currently recruiting participants. Participants can have received prior treatment with proteasome inhibitors. Participants with prior bortezomib treatment are eligible regardless of refractory status. Prior carfilzomib treatment is allowed, provided that the patients achieve at least a partial response to prior carfilzomib and that there is a treatment free interval of at least 6 months.	

Supplementary table S1- Clinical trials of combination therapies with 20S CP inhibitors, involving patients with relapsed and/or refractory disease, and who were previously treated with a 20S CP inhibitor. Source: ClinicalTrials (https://clinicaltrials.gov/).

Study (code)	Title	20S CP inhibitor	Other drugs	Purpose	Observations	Ref.
NCT05137054	Phase 1b Study of REGN5458 (Anti-BCMA x Anti-CD3 Bispecific Antibody) Plus Other Cancer Treatments for Patients with Relapsed/Refractory Multiple Myeloma	Bortezomib or carfilzomib	REGN5458 and dexamethasone	To evaluate the safety and tolerability, and determine the recommended dose of REGN5458 for the expansion portion in combination with each one of the following regimens:<br>-daratumumab plus dexamethasone,<br>-carfilzomib plus dexamethasone,<br>-lenalidomide plus dexamethasone,<br>-bortezomib plus dexamethasone.<br>To evaluate the preliminary efficacy, pharmacokinetic properties and to assess the immunogenicity of these regimens, in patients with relapsed/refractory MM.	This study is not yet recruiting. Participants must have progression of disease following at least 3 lines of therapy or least 2 lines of therapy and prior exposure to at least one anti-CD38 antibody, one immunomodulatory drug and one proteasome inhibitor or double-refractory to a proteasome inhibitor and an immunomodulatory drug, or to their combination.<br>For the regimen that includes carfilzomib, prior treatment with carfilzomib is allowed if previously tolerated at the approved full dose. However, participants cannot be carfilzomib refractory.<br>For the bortezomib-containing regimen, prior treatment with bortezomib is allowed if previously tolerated at the approved full dose. However, participants cannot be bortezomib refractory.	

1. Holkova, B.; Kmieciak, M.; Perkins, E.B.; Bose, P.; Baz, R.C.; Roodman, G.D.; Stuart, R.K.; Ramakrishnan, V.; Wan, W.; Peer, C.J.; et al. Phase I trial of bortezomib (PS-341; NSC 681239) and "nonhybrid" (bolus) infusion schedule of alvocidib (flavopiridol; NSC 649890) in patients with recurrent or refractory indolent B-cell neoplasms. *Clin. Cancer Res.* **2014**, *20*, 5652–62, doi:10.1158/1078-0432.CCR-14-0805.

2. Siegel, D.S.; Krishnan, A.; Lonial, S.; Chatta, G.; Alsina, M.; Jagannath, S.; Richardson, P.; Hohl, R.J.; Lust, J.A.; Bensinger, W.; et al. Phase II trial of SCIO-469 as monotherapy (M) or in combination with bortezomib (MB) in relapsed refractory multiple myeloma (MM). *Blood* **2006**, *108*, 1022A-1022A.

3. Weber, D.M.; Graef, T.; Hussein, M.; Sobecks, R.M.; Schiller, G.J.; Lupinacci, L.; Hardwick, J.S.; Jagannath, S. Phase I Trial of Vorinostat Combined with Bortezomib for the Treatment of Relapsing and/or Refractory Multiple Myeloma. *Clin. Lymphoma Myeloma Leuk.* **2012**, *12*, 319–324, doi:10.1016/j.clml.2012.07.007.

4. Richardson, P.G.; Weller, E.; Jagannath, S.; Avigan, D.E.; Alsina, M.; Schlossman, R.L.; Mazumder, A.; Munshi, N.C.; Ghobrial, I.M.; Doss, D.; et al. Multicenter, phase I, dose-escalation trial of lenalidomide plus bortezomib for relapsed and relapsed/refractory multiple myeloma. *J. Clin. Oncol.* **2009**, *27*, 5713–9, doi:10.1200/JCO.2009.22.2679.

5. Gerecitano, J.; Portlock, C.; Hamlin, P.; Moskowitz, C.H.; Noy, A.; Straus, D.; Schulman, P.; Dumitrescu, O.; Sarasohn, D.; Pappanicholaou, J.; et al. Phase I trial of weekly and twice-weekly bortezomib with rituximab, cyclophosphamide, and prednisone in relapsed or refractory non-Hodgkin lymphoma. *Clin. Cancer Res.* **2011**, *17*, 2493–501, doi:10.1158/1078-0432.CCR-10-1498.

6. Badros, A.; Burger, A.M.; Philip, S.; Niesvizky, R.; Kolla, S.S.; Goloubeva, O.; Harris, C.; Zweibel, J.; Wright, J.J.; Espinoza-Delgado, I.; et al. Phase I Study Of Vorinostat in Combination with Bortezomib for Relapsed and Refractory Multiple Myeloma. *Clin Cancer Res. Clin Cancer Res. August* **2009**, *15*, 5250–5257, doi:10.1158/1078-0432.CCR-08-2850.

7. Berenson, J.R.; Yellin, O.; Patel, R.; Duvivier, H.; Nassir, Y.; Mapes, R.; Abaya, C.D.; Swift, R.A. A phase I study of samarium lexidronam/bortezomib combination therapy for the treatment of relapsed or refractory multiple myeloma. *Clin. Cancer Res.* **2009**, *15*, 1069–75, doi:10.1158/1078-0432.CCR-08-1261.

8. Campbell, R.A.; Gordon, M.S.; Betancourt, O.; Juarez, J.; Donate, F.; Mazar, A.; Berenson, J.R. *ATN-224, an orally available small molecule inhibitor of SOD1, inhibits multiple signaling pathways associated with myeloma progression and has antitumor activity in a murine model of refractory myeloma growth*; Waverly Press, 2006; Vol. 66;.

9. Berenson, J.R.; Ervin, T.; Boccia, R. V.; Batuman, O.; Swift, R.; Abaya, C.D.; Mazar, A.P.; Gordon, G.S.; Reich, S.D.; Callahan, J.A. Results from the Phase I Portion of a Phase I/II Study of ATN-224 and Bortezomib for Patients with Multiple Myeloma Relapsed from or Refractory to Bortezomib. *Blood* **2008**, *112*, 5185–5185, doi:10.1182/blood.V112.11.5185.5185.

10. Richardson, P.G.; Xie, W.; Jagannath, S.; Jakubowiak, A.; Lonial, S.; Raje, N.S.; Alsina, M.; Ghobrial, I.M.; Schlossman, R.L.; Munshi, N.C.; et al. A phase 2 trial of lenalidomide, bortezomib, and dexamethasone in patients with relapsed and relapsed/refractory myeloma. *Blood* **2014**, *123*, 1461–9, doi:10.1182/blood-2013-07-517276.

11. Richardson, P.G.; Wolf, J.; Jakubowiak, A.; Zonder, J.; Lonial, S.; Irwin, D.; Densmore, J.; Krishnan, A.; Raje, N.; Bar, M.; et al. Perifosine plus bortezomib and dexamethasone in patients with relapsed/refractory multiple myeloma previously treated with bortezomib: results of a multicenter phase I/II trial. *J. Clin. Oncol.* **2011**, *29*, 4243–9, doi:10.1200/JCO.2010.33.9788.

12. Goy, A.; Hernandez-Ilzaliturri, F.J.; Kahl, B.; Ford, P.; Protomastro, E.; Berger, M. A phase I/II study of the pan Bcl-2 inhibitor obatoclax mesylate plus bortezomib for relapsed or refractory mantle cell

lymphoma. *Leuk. Lymphoma* **2014**, *55*, 2761–8, doi:10.3109/10428194.2014.907891.

13. Moosmann, P.; Heizmann, M.; Kotrubczik, N.; Wernli, M.; Bargetzi, M. Weekly treatment with a combination of bortezomib and bendamustine in relapsed or refractory indolent non-Hodgkin lymphoma. *Leuk. Lymphoma* **2010**, *51*, 149–152, doi:10.3109/10428190903275602.

14. Harrison, S.J.; Quach, H.; Link, E.; Seymour, J.F.; Ritchie, D.S.; Ruell, S.; Dean, J.; Januszewicz, H.; Johnstone, R.; Neeson, P.; et al. A high rate of durable responses with romidepsin, bortezomib, and dexamethasone in relapsed or refractory multiple myeloma. *Blood* **2011**, *118*, 6274–6283, doi:10.1182/blood-2011-03-339879.

15. Ghobrial, I.M.; Weller, E.; Vij, R.; Munshi, N.C.; Banwait, R.; Bagshaw, M.; Schlossman, R.; Leduc, R.; Chuma, S.; Kunsman, J.; et al. Weekly bortezomib in combination with temsirolimus in relapsed or relapsed and refractory multiple myeloma: a multicentre, phase 1/2, open-label, dose-escalation study. *Lancet Oncol.* **2011**, *12*, 263–272, doi:10.1016/S1470-2045(11)70028-6.

16. Richardson, P.G.; Badros, A.Z.; Jagannath, S.; Tarantolo, S.; Wolf, J.L.; Albitar, M.; Berman, D.; Messina, M.; Anderson, K.C. Tanespimycin with bortezomib: activity in relapsed/refractory patients with multiple myeloma. *Br. J. Haematol.* **2010**, *150*, no-no, doi:10.1111/j.1365-2141.2010.08264.x.

17. San-Miguel, J.F.; Richardson, P.G.; Günther, A.; Sezer, O.; Siegel, D.; Bladé, J.; LeBlanc, R.; Sutherland, H.; Sopala, M.; Mishra, K.K.; et al. Phase Ib study of panobinostat and bortezomib in relapsed or relapsed and refractory multiple myeloma. *J. Clin. Oncol.* **2013**, *31*, 3696–703, doi:10.1200/JCO.2012.46.7068.

18. Wang, M.; Martin, T.; Bensinger, W.; Alsina, M.; Siegel, D.S.; Kavalerchik, E.; Huang, M.; Orlowski, R.Z.; Niesvizky, R. Phase 2 dose-expansion study (PX-171-006) of carfilzomib, lenalidomide, and low-dose dexamethasone in relapsed or progressive multiple myeloma. *Blood* **2013**, *122*, 3122–8, doi:10.1182/blood-2013-07-511170.

19. Niesvizky, R.; Martin, T.G.; Bensinger, W.I.; Alsina, M.; Siegel, D.S.; Kunkel, L.A.; Wong, A.F.; Lee, S.; Orlowski, R.Z.; Wang, M.; et al. Phase Ib dose-escalation study (PX-171-006) of carfilzomib, lenalidomide, and low-dose dexamethasone in relapsed or progressive multiple myeloma. *Clin. Cancer Res.* **2013**, *19*, 2248–56, doi:10.1158/1078-0432.CCR-12-3352.

20. Siegel, D.S.; Dimopoulos, M.; Jagannath, S.; Goldschmidt, H.; Durrant, S.; Kaufman, J.L.; Leleu, X.; Nagler, A.; Offner, F.; Graef, T.; et al. VANTAGE 095: An International, Multicenter, Open-Label Study of Vorinostat (MK-0683) in Combination with Bortezomib in Patients with Relapsed and Refractory Multiple Myeloma. *Clin. Lymphoma Myeloma Leuk.* **2016**, *16*, 329-334.e1, doi:10.1016/j.clml.2016.02.042.

21. Lonial, S.; Kaufman, J.; Tighiouart, M.; Nooka, A.; Langston, A.A.; Heffner, L.T.; Torre, C.; McMillan, S.; Renfroe, H.; Harvey, R.D.; et al. A Phase I/II Trial Combining High-Dose Melphalan and Autologous Transplant with Bortezomib for Multiple Myeloma: A Dose- and Schedule-Finding Study. *Clin. Cancer Res.* **2010**, *16*, 5079–5086, doi:10.1158/1078-0432.CCR-10-1662.

22. Ogawa, Y.; Ogura, M.; Tobinai, K.; Ando, K.; Suzuki, T.; Watanabe, T.; Ohmachi, K.; Uchida, T.; Hanson, M.E.; Tanaka, Y.; et al. A phase I study of vorinostat combined with bortezomib in Japanese patients with relapsed or refractory multiple myeloma. *Int. J. Hematol.* **2016**, *103*, 25–33, doi:10.1007/s12185-015-1897-7.

23. Richardson, P.G.; Nagler, A.; Ben-Yehuda, D.; Badros, A.; Hari, P.N.; Hajek, R.; Spicka, I.; Kaya, H.; LeBlanc, R.; Yoon, S.; et al. Randomized, placebo-controlled, phase 3 study of perifosine combined with bortezomib and dexamethasone in patients with relapsed, refractory multiple myeloma previously treated with bortezomib. *eJHaem* **2020**, *1*, 94–102, doi:10.1002/jha2.4.

24. Richardson, P.G.; Schlossman, R.L.; Alsina, M.; Weber, D.M.; Coutre, S.E.; Gasparetto, C.;

Mukhopadhyay, S.; Ondovik, M.S.; Khan, M.; Paley, C.S.; et al. PANORAMA 2: panobinostat in combination with bortezomib and dexamethasone in patients with relapsed and bortezomib-refractory myeloma. *Blood* **2013**, *122*, 2331–7, doi:10.1182/blood-2013-01-481325.

25. Dunn, T.J.; Dinner, S.; Price, E.; Coutré, S.E.; Gotlib, J.; Hao, Y.; Berube, C.; Medeiros, B.C.; Liedtke, M. A phase 1, open-label, dose-escalation study of pralatrexate in combination with bortezomib in patients with relapsed/refractory multiple myeloma. *Br. J. Haematol.* **2016**, *173*, 253–259, doi:10.1111/bjh.13946.

26. Ghobrial, I.M.; Redd, R.; Armand, P.; Banwait, R.; Boswell, E.; Chuma, S.; Huynh, D.; Sacco, A.; Roccaro, A.M.; Perilla-Glen, A.; et al. Phase I/II trial of everolimus in combination with bortezomib and rituximab (RVR) in relapsed/refractory Waldenstrom macroglobulinemia. *Leukemia* **2015**, *29*, 2338–2346, doi:10.1038/leu.2015.164.

27. Berenson, J.R.; Yellin, O.; Kazamel, T.; Hilger, J.D.; Chen, C.-S.; Cartmell, A.; Woliver, T.; Flam, M.; Bravin, E.; Nassir, Y.; et al. A phase 2 study of pegylated liposomal doxorubicin, bortezomib, dexamethasone and lenalidomide for patients with relapsed/refractory multiple myeloma. *Leukemia* **2012**, *26*, 1675–1680, doi:10.1038/leu.2012.51.

28. Driessen, C.; Kraus, M.; Joerger, M.; Rosing, H.; Bader, J.; Hitz, F.; Berset, C.; Xyrafas, A.; Hawle, H.; Berthod, G.; et al. Treatment with the HIV protease inhibitor nelfinavir triggers the unfolded protein response and may overcome proteasome inhibitor resistance of multiple myeloma in combination with bortezomib: a phase I trial (SAKK 65/08). *Haematologica* **2016**, *101*, 346–55, doi:10.3324/haematol.2015.135780.

29. Ludwig, H.; Kasparu, H.; Leitgeb, C.; Rauch, E.; Linkesch, W.; Zojer, N.; Greil, R.; Seebacher, A.; Pour, L.; Weißmann, A.; et al. Bendamustine-bortezomib-dexamethasone is an active and well-tolerated regimen in patients with relapsed or refractory multiple myeloma. *Blood* **2014**, *123*, 985–91, doi:10.1182/blood-2013-08-521468.

30. Raje, N.; Hari, P.N.; Landau, H.; Richardson, P.G.; Rosenblatt, J.; Couture, N.; Lyons, J.F.; Langford, G.; Yule, M. A Phase I/II Open-Label Multicenter Study Of The Cyclin Kinase Inhibitor AT7519M Alone and In Combination with Bortezomib In Patients with Previously Treated Multiple Myeloma. *Blood* **2013**, *122*, 1976–1976, doi:10.1182/blood.V122.21.1976.1976.

31. Hofmeister, C.C.; Berdeja, J.G.; Vesole, D.H.; Suvannasankha, A.; Parrott, T.; Rafat, A. 3052 TG02, an Oral CDK9-Inhibitor, in Combination with Carfilzomib Demonstrated Objective Responses in Carfilzomib Refractory Multiple Myeloma Patients. *Blood* **2015**, *126*, 3052.

32. Roboz, G.J.; Khoury, H.J.; Shammo, J.M.; Syto, M.; Burrows, F.; Zaknoen, S.L.; Jabbour, E. Phase I dose escalation study of TG02 in patients with advanced hematologic malignancies. Available online: https://meetinglibrary.asco.org/record/69732/abstract (accessed on Oct 16, 2018).

33. Chari, A.; Htut, M.; Zonder, J. a.; Fay, J.W.; Jakubowiak, A.J.; Levy, J.B.; Lau, K.; Burt, S.M.; Tunquist, B.J.; Hilder, B.W.; et al. A phase 1 dose-escalation study of filanesib plus bortezomib and dexamethasone in patients with recurrent/refractory multiple myeloma. *Cancer* **2016**, *122*, 3327–3335, doi:10.1002/cncr.30174.

34. Fenske, T.S.; Shah, N.M.; Kim, K.M.; Saha, S.; Zhang, C.; Baim, A.E.; Farnen, J.P.; Onitilo, A.A.; Blank, J.H.; Ahuja, H.; et al. A phase 2 study of weekly temsirolimus and bortezomib for relapsed or refractory B-cell non-Hodgkin lymphoma: A Wisconsin Oncology Network study. *Cancer* **2015**, *121*, 3465–3471, doi:10.1002/cncr.29502.

35. Vesole, D.H.; Bilotti, E.; Richter, J.R.; McNeill, A.; McBride, L.; Raucci, L.; Anand, P.; Bednarz, U.; Ivanovski, K.; Smith, J.; et al. Phase I study of carfilzomib, lenalidomide, vorinostat, and dexamethasone in patients with relapsed and/or refractory multiple myeloma. *Br. J. Haematol.* **2015**, *171*, 52–59, doi:10.1111/bjh.13517.

36. Manasanch, E.E.; Shah, J.J.; Lee, H.C.; Weber, D.M.; Thomas, S.K.; Amini, B.; Olsem, J.; Crumpton, B.; Morphey, A.; Berkova, Z.; et al. Phase I/Ib study of carfilzomib and panobinostat with or without dexamethasone in patients with relapsed/refractory multiple myeloma. *Haematologica* **2019**, haematol.2019.225375, doi:10.3324/haematol.2019.225375.

37. Lee, H.C.; Shah, J.J.; Feng, L.; Manasanch, E.E.; Lu, R.; Morphey, A.; Crumpton, B.; Patel, K.K.; Wang, M.L.; Alexanian, R.; et al. A phase 1 study of filanesib, carfilzomib, and dexamethasone in patients with relapsed and/or refractory multiple myeloma. *Blood Cancer J.* **2019**, *9*, 80, doi:10.1038/s41408-019-0240-6.

38. Montanari, F.; Lu, M.; Marcus, S.; Saran, A.; Malankar, A.; Mazumder, A. A Phase II Trial of Chloroquine in Combination with Bortezomib and Cyclophosphamide in Patients with Relapsed and Refractory Multiple Myeloma. *Blood* **2014**, *124*, 5775–5775, doi:10.1182/blood.V124.21.5775.5775.

39. Berdeja, J.G.; Hart, L.L.; Mace, J.R.; Arrowsmith, E.R.; Essell, J.H.; Owera, R.S.; Hainsworth, J.D.; Flinn, I.W. Phase I/II study of the combination of panobinostat and carfilzomib in patients with relapsed/refractory multiple myeloma. *Haematologica* **2015**, *100*, 670–6, doi:10.3324/haematol.2014.119735.

40. Berdeja, J.G.; Gregory, T.B.; Faber, E.A.; Matous, J. V.; Hart, L.L.; Mace, J.R.; Peyton, J.D.; Essell, J.H.; Arrowsmith, E.R.; Flinn, I.W. A Phase I/II Study of the Combination of Panobinostat (PAN) and Carfilzomib (CFZ) in Patients (pts) with Relapsed or Relapsed/Refractory Multiple Myeloma (MM): Comparison of Two Expansion Cohorts. *Blood* **2015**, *126*, 1825–1825, doi:10.1002/ajh.26088.

41. Berdeja, J.G.; Gregory, T.K.; Faber, E.A.; Hart, L.L.; Mace, J.R.; Arrowsmith, E.R.; Flinn, I.W.; Matous, J. V. A phase I/II study of the combination of panobinostat and carfilzomib in patients with relapsed or relapsed/refractory multiple myeloma: Final analysis of second dose-expansion cohort. *Am. J. Hematol.* **2021**, *96*, 428–435, doi:10.1002/ajh.26088.

42. Laubach, J.P.; Liu, C.-J.; Raje, N.S.; Yee, A.J.; Armand, P.; Schlossman, R.L.; Rosenblatt, J.; Hedlund, J.; Martin, M.G.; Reynolds, C.H.; et al. A Phase 1/2 Study of evofosfamide, A Hypoxia-Activated Prodrug with or without Bortezomib in Subjects with Relapsed/Refractory Multiple Myeloma. *Clin. Cancer Res.* **2018**, doi:10.1158/1078-0432.CCR-18-1325.

43. Moreau, P.; Chanan-Khan, A.; Roberts, A.W.; Agarwal, A.B.; Facon, T.; Kumar, S.; Touzeau, C.; Punnoose, E.A.; Cordero, J.; Munasinghe, W.; et al. Promising efficacy and acceptable safety of venetoclax plus bortezomib and dexamethasone in relapsed/refractory MM. *Blood* **2017**, *130*, 2392–2400, doi:10.1182/blood-2017-06-788323.

44. Vesole, D.H.; Richter, J.; Biran, N.; McBride, L.; Anand, P.; Huang, M.; Kumeli, A.-Z.; Klippel, Z.; Iskander, K.; Siegel, D.S. Carfilzomib as salvage therapy in Waldenstrom macroglobulinemia: a case series. *Leuk. Lymphoma* **2018**, *59*, 259–261, doi:10.1080/10428194.2017.1321749.

45. Ailawadhi, S.; Sexton, R.; Lentzsch, S.; Abidi, M.H.; Voorhees, P.M.; Cohen, A.D.; Rohren, E.M.; Heitner, S.; Kelly, K.; Mackler, N.J.; et al. Low-dose versus High-dose Carfilzomib with Dexamethasone (S1304) in Patients with Relapsed-Refractory Multiple Myeloma. *Clin. Cancer Res.* **2020**, *26*, 3969–3978, doi:10.1158/1078-0432.CCR-19-1997.

46. Chari, A.; Larson, S.; Holkova, B.; Cornell, R.F.; Gasparetto, C.; Karanes, C.; Matous, J. V.; Niesvizky, R.; Valent, J.; Lunning, M.; et al. Phase 1 trial of ibrutinib and carfilzomib combination therapy for relapsed or relapsed and refractory multiple myeloma. *Leuk. Lymphoma* **2018**, 1–7, doi:10.1080/10428194.2018.1443337.

47. Chari, A.; Cornell, R.F.; Gasparetto, C.; Karanes, C.; Matous, J. V.; Niesvizky, R.; Lunning, M.; Usmani, S.Z.; Anderson, L.D.; Chhabra, S.; et al. Final analysis of a phase 1/2b study of ibrutinib combined with carfilzomib/dexamethasone in patients with relapsed/refractory multiple myeloma. *Hematol. Oncol.* **2020**, hon.2723, doi:10.1002/hon.2723.

48. Laubach, J.P.; Tuchman, S.A.; Rosenblatt, J.M.; Mitsiades, C.S.; Colson, K.; Masone, K.; Warren, D.; Redd, R.A.; Grayson, D.; Richardson, P.G. Phase 1 open-label study of panobinostat, lenalidomide, bortezomib + dexamethasone in relapsed and relapsed/refractory multiple myeloma. *Blood Cancer J.* **2021**, *11*, 20, doi:10.1038/s41408-021-00407-5.

49. Zonder, J.A.; Usmani, S.; Scott, E.C.; Hofmeister, C.C.; Lendvai, N.; Berdeja, J.G.; Anderson, L.D.; Hari, P.; Singhal, S.; Orloff, G.; et al. Phase 2 Study of Carfilzomib with or without Filanesib in Patients with Advanced Multiple Myeloma (MM). *Am. Soc. Hematol. Annu. Meet. 2015* **2015**.

50. Shah, J.; Usmani, S.; Stadtmauer, E.A.; Rifkin, R.M.; Berenson, J.R.; Berdeja, J.G.; Lyons, R.M.; Klippel, Z.; Chang, Y.-L.; Niesvizky, R. Oprozomib, pomalidomide, and Dexamethasone in Patients with Relapsed and/or Refractory Multiple Myeloma. *Clin. Lymphoma Myeloma Leuk.* **2019**, *19*, 570-578.e1, doi:10.1016/j.clml.2019.05.017.

51. Chari, A.; Martinez-Lopez, J.; Mateos, M.-V.; Bladé, J.; Benboubker, L.; Oriol, A.; Arnulf, B.; Rodriguez-Otero, P.; Pineiro, L.; Jakubowiak, A.; et al. Daratumumab plus carfilzomib and dexamethasone in patients with relapsed or refractory multiple myeloma. *Blood* **2019**, *134*, 421–431, doi:10.1182/blood.2019000722.

52. Leleu, X.; Beksac, M.; Chou, T.; Dimopoulos, M.; Yoon, S.-S.; Prince, H.M.; Pour, L.; Shelekhova, T.; Chari, A.; Khurana, M.; et al. Efficacy and safety of weekly carfilzomib (70 mg/m 2), dexamethasone, and daratumumab (KdD70) is comparable to twice-weekly KdD56 while being a more convenient dosing option: a cross-study comparison of the CANDOR and EQUULEUS studies. *Leuk. Lymphoma* **2021**, *62*, 358–367, doi:10.1080/10428194.2020.1832672.

53. Li, B.; Chen, P.; Ge, X.; Zhang, X.; Fu, J. Granulocyte-Colony Stimulating Factor (G-CSF) in Combination with Bortezomib, Cyclophosphamide and Dexamethasone in Patients with Multiple Myeloma. *Blood* **2014**, *124*, doi:10.1182/blood.V124.21.5768.5768.

54. Gay, F.; Günther, A.; Offidani, M.; Engelhardt, M.; Salvini, M.; Montefusco, V.; Patriarca, F.; Aquino, S.; Pönisch, W.; Spada, S.; et al. Carfilzomib, bendamustine, and dexamethasone in patients with advanced multiple myeloma: The EMN09 phase 1/2 study of the European Myeloma Network. *Cancer* **2021**, *127*, 3413–3421, doi:10.1002/cncr.33647.

55. Lee, H.C.; Feng, L.; Oriabure, O.; Graham, V.; Chen, W.; Badillo, M.; Lu, R.; Lee, H.J.; Jain, P.; Manasanch, E.E.; et al. A phase one trial of carfilzomib, bendamustine, and dexamethasone in relapsed and/or refractory multiple myeloma. *Am. J. Hematol.* **2021**, *96*, doi:10.1002/ajh.26178.

56. Ocio, E.M.; Mateos, M.-V.; Prosper, F.; Martin, J.; Rocafiguera, A.O.; Jarque, I.; Iglesias, R.; Motlloo, C.; Sole, M.; Rodriguez-Otero, P.; et al. Phase I study of plitidepsin in combination with bortezomib and dexamethasone in patients with relapsed and/or refractory multiple myeloma. *J. Clin. Oncol.* **2016**, *34*, 8006, doi:10.1200/JCO.2016.34.15_suppl.8006 Journal of Clinical Oncology 34, no. 15_suppl (May 2016) 8006-8006.

57. Sborov, D.W.; Viola, D.; Dona, A.A.; Nooka, A.K.; Kaufman, J.L.; Lonial, S.; Boise, L.; Dhodapkar, M. V.; Joseph, N.; Pichiorri, F.; et al. Oncolytic virus pelareorep plus carfilzomib phase I trial in carfilzomib-refractory patients (NCI 9603): Responses with cytokine storm. *J. Clin. Oncol.* **2020**, *38*, doi:0.1200/JCO.2020.38.15_suppl.8535.

58. Spencer, A.; Harrison, S.; Zonder, J.; Badros, A.; Laubach, J.; Bergin, K.; Khot, A.; Zimmerman, T.; Chauhan, D.; Levin, N.; et al. A phase 1 clinical trial evaluating marizomib, pomalidomide and low-dose dexamethasone in relapsed and refractory multiple myeloma (NPI-0052-107): final study results. *Br. J. Haematol.* **2018**, *180*, 41–51, doi:10.1111/bjh.14987.

59. Krishnan, A.; Kapoor, P.; Palmer, J.M.; Tsai, N.-C.; Kumar, S.; Lonial, S.; Htut, M.; Karanes, C.; Nathwani, N.; Rosenzweig, M.; et al. Phase I/II trial of the oral regimen ixazomib, pomalidomide, and dexamethasone in relapsed/refractory multiple myeloma. *Leukemia* **2017**,

doi:10.1038/leu.2017.352.

60. Popat, R.; Brown, S.R.; Flanagan, L.; Hall, A.; Gregory, W.; Kishore, B.; Streetly, M.; Oakervee, H.; Yong, K.; Cook, G.; et al. Bortezomib, thalidomide, dexamethasone, and panobinostat for patients with relapsed multiple myeloma (MUK-six): a multicentre, open-label, phase 1/2 trial. *Lancet Haematol.* **2016**, *3*, e572–e580, doi:10.1016/S2352-3026(16)30165-X.

61. Popat, R.; Brown, S.R.; Flanagan, L.; Hall, A.; Gregory, W.; Kishore, B.; Streetly, M.; Oakervee, H.; Yong, K.; Cook, G.; et al. Extended follow-up and the feasibility of Panobinostat maintenance for patients with Relapsed Multiple Myeloma treated with Bortezomib, Thalidomide, Dexamethasone plus Panobinostat (MUK six open label, multi-centre phase I/II Clinical Trial). *Br. J. Haematol.* **2018**, doi:10.1111/bjh.15551.

62. Driessen, C.; Müller, R.; Novak, U.; Cantoni, N.; Betticher, D.; Mach, N.; Rüfer, A.; Mey, U.; Samaras, P.; Ribi, K.; et al. Promising activity of nelfinavir-bortezomib-dexamethasone (NeVd) in proteasome inhibitor-refractory multiple myeloma. *Blood* **2018**, *41*, blood-2018-05-851170, doi:10.1182/blood-2018-05-851170.

63. Jakubowiak, A.J.; Jasielec, J.K.; Rosenbaum, C.A.; Cole, C.E.; Chari, A.; Mikhael, J.; Nam, J.; McIver, A.; Severson, E.; Stephens, L.A.; et al. Phase 1 study of selinexor plus carfilzomib and dexamethasone for the treatment of relapsed/refractory multiple myeloma. *Br. J. Haematol.* **2019**, *186*, 549–560, doi:10.1111/bjh.15969.

64. Martin, T.G.; Shah, N.; Richter, J.; Vesole, D.H.; Wong, S.W.; Huang, C.; Madduri, D.; Jagannath, S.; Siegel, D.S.; Biran, N.; et al. Phase 1b trial of isatuximab, an anti-CD38 monoclonal antibody, in combination with carfilzomib as treatment of relapsed/refractory multiple myeloma. *Cancer* **2021**, *127*, 1816–1826, doi:10.1002/cncr.33448.

65. Bahlis, N.J.; Sutherland, H.; White, D.; Sebag, M.; Lentzsch, S.; Kotb, R.; Venner, C.P.; Gasparetto, C.; Del Col, A.; Neri, P.; et al. Selinexor plus low-dose bortezomib and dexamethasone for patients with relapsed or refractory multiple myeloma. *Blood* **2018**, *132*, 2546–2554, doi:10.1182/blood-2018-06-858852.

66. Moreau, P.; Mateos, M.V.; Berenson, J.R.; Weisel, K.; Lazzaro, A.; Song, K.; Dimopoulos, M. a.; Huang, M.; Zahlten-Kumeli, A.; Stewart, a. K. Once weekly versus twice weekly carfilzomib dosing in patients with relapsed and refractory multiple myeloma (A.R.R.O.W.): interim analysis results of a randomised, phase 3 study. *Lancet Oncol.* **2018**, *19*, 953–964, doi:10.1016/S1470-2045(18)30354-1.

67. Dimopoulos, M.A.; Moreau, P.; Iida, S.; Huang, S.-Y.; Takezako, N.; Chng, W.J.; Zahlten-Kumeli, A.; Sersch, M.A.; Li, J.; Huang, M.; et al. Outcomes for Asian patients with multiple myeloma receiving once- or twice-weekly carfilzomib-based therapy: a subgroup analysis of the randomized phase 3 ENDEAVOR and A.R.R.O.W. Trials. *Int. J. Hematol.* **2019**, *110*, 466–473, doi:10.1007/s12185-019-02704-z.

68. Moreau, P.; Kumar, S.; Boccia, R.; Iida, S.; Goldschmidt, H.; Cocks, K.; Trigg, A.; Zahlten-Kumeli, A.; Yucel, E.; Panjabi, S.S.; et al. Convenience, satisfaction, health-related quality of life of once-weekly 70 mg/m2 vs. twice-weekly 27 mg/m2 carfilzomib (randomized A.R.R.O.W. study). *Leukemia* **2019**, *33*, 2934–2946, doi:10.1038/s41375-019-0480-2.

69. Dhakal, B.; D'Souza, A.; Hamadani, M.; Arce-Lara, C.; Schroeder, K.; Chhabra, S.; Shah, N.N.; Gauger, K.; Keaton, T.; Pasquini, M.; et al. Phase I/II trial of bendamustine, ixazomib, and dexamethasone in relapsed/refractory multiple myeloma. *Blood Cancer J.* **2019**, *9*, 56, doi:10.1038/s41408-019-0219-3.

70. Yee, A.J.; Laubach, J.; Campagnaro, E.L.; Lipe, B.; Nadeem, O.; Cole, C.; O'Donnell, E.; Schlossman, R.L.; Bianchi, G.; Branagan, A.R.; et al. A phase II study of elotuzumab in combination with pomalidomide, bortezomib, and dexamethasone in relapsed and refractory multiple myeloma. *J. Clin. Oncol.* **2018**, *36*, 8012–8012, doi:10.1200/JCO.2018.36.15_suppl.8012.

71. Du, J.; Fang, B.; Li, J.; Jin, J.; Wang, S.; Zou, D.; Cai, Z.; Wang, H.; Hu, J.; Li, W.; et al. A study of carfilzomib and dexamethasone in patients with relapsed and refractory multiple myeloma in China. *Int. J. Hematol.* **2021**, *113*, 422–429, doi:10.1007/s12185-020-03044-z.

72. Siegel, D.; Weisel, K.; Zahlten-Kumeli, A.; Medhekar, R.; Ding, B.; Leleu, X. Health-related quality of life outcomes from the CANDOR study in patients with relapsed or refractory multiple myeloma. *Leuk. Lymphoma* **2021**, *62*, 3002–3010, doi:10.1080/10428194.2021.1941927.

73. Suzuki, K.; Min, C.-K.; Kim, K.; Lee, J.-J.; Shibayama, H.; Ko, P.-S.; Huang, S.-Y.; Li, S.-S.; Ding, B.; Khurana, M.; et al. Carfilzomib, dexamethasone, and daratumumab in Asian patients with relapsed or refractory multiple myeloma: post hoc subgroup analysis of the phase 3 CANDOR trial. *Int. J. Hematol.* **2021**, *114*, 653–663, doi:10.1007/s12185-021-03204-9.

74. Dimopoulos, M.; Quach, H.; Mateos, M.-V.; Landgren, O.; Leleu, X.; Siegel, D.; Weisel, K.; Yang, H.; Klippel, Z.; Zahlten-Kumeli, A.; et al. Carfilzomib, dexamethasone, and daratumumab versus carfilzomib and dexamethasone for patients with relapsed or refractory multiple myeloma (CANDOR): results from a randomised, multicentre, open-label, phase 3 study. *Lancet* **2020**, *396*, 186–197, doi:10.1016/S0140-6736(20)30734-0.

75. Quach, H.; Nooka, A.; Samoylova, O.; Venner, C.P.; Kim, K.; Facon, T.; Spencer, A.; Usmani, S.Z.; Grosicki, S.; Suzuki, K.; et al. Carfilzomib, dexamethasone and daratumumab in relapsed or refractory multiple myeloma: results of the phase III study CANDOR by prior lines of therapy. *Br. J. Haematol.* **2021**, *194*, 784–788, doi:10.1111/bjh.17541.

76. Bhutani, M.; Robinson, M.; Atrash, S.; Foureau, D.M.; Rigby, K.; Friend, R.; Paul, B.; Symanowski, J.T.; Druhan, L.J.; Norek, S.; et al. Phase II Trial of Elotuzumab in Combination with Carfilzomib, Lenalidomide and Dexamethasone for the Treatment of Relapsed/Refractory Multiple Myeloma (RRMM) after One Prior Line of Therapy. *Blood* **2021**, *138*, 2739–2739, doi:10.1182/blood-2021-148508.

77. Cohen, Y.C.; Zada, M.; Wang, S.-Y.; Bornstein, C.; David, E.; Moshe, A.; Li, B.; Shlomi-Loubaton, S.; Gatt, M.E.; Gur, C.; et al. Identification of resistance pathways and therapeutic targets in relapsed multiple myeloma patients through single-cell sequencing. *Nat. Med.* **2021**, *27*, 491–503, doi:10.1038/s41591-021-01232-w.
